# Intracranial angioplasty with a self-expandable stent for intracranial atherosclerotic stenosis: Systematic review and meta-analysis

**DOI:** 10.3389/fneur.2022.1074228

**Published:** 2023-01-09

**Authors:** Cai Zhong, Shijian Chen, Jian Zhang, Shuguang Luo, Ziming Ye, Yayuan Liu, Linlin Pang, Zimei Dong, Chao Qin

**Affiliations:** Department of Neurology, The First Affiliated Hospital of Guangxi Medical University, Nanning, Guangxi, China

**Keywords:** intracranial arteriosclerosis, angioplasty, stents, systematic review, meta-analysis

## Abstract

**Background:**

Intracranial angioplasty with a self-expandable stent (SES) is an important endovascular therapy for symptomatic intracranial arterial stenosis. We sought to update the evaluation of the perioperative safety and long-term outcomes of self-expandable stent for the treatment of symptomatic intracranial arterial stenosis.

**Methods:**

We comprehensively searched the published literature from each database through Sept 16, 2022, for the PubMed, EMBASE, Web of Science, Cochrane, and Clinical Trials databases. The characteristics of the studies and patients, perioperative complications, and long-term outcomes were extracted. The pooled outcomes and 95% confidence intervals (CIs) were estimated by Stata Statistical Software 14.0.

**Results:**

A total of 4,632 patients from 58 studies were included. The pooled rate of perioperative stroke or death was 6.32% (95% CI 5.04-7.72%); ischemic stroke beyond 30 days through 1 year was 2.72% (95% CI 1.41–4.38%). Perioperative complications differed between the 2014-2022 and 2005-2013 subgroups, as did long-term outcomes between the off-label SES and Wingspan subgroups.

**Conclusion:**

The perioperative complications of intracranial angioplasty with SES have been reduced, but the risk of perioperative stroke or death is still higher than that of aggressive medical therapy, and additional studies are needed to determine whether it has better long-term outcomes than aggressive medical therapy. Perioperative complications varied between the 2014-2022 and 2005-2013 subgroups, as did long-term outcomes between the off-label SES and Wingspan subgroups. Given the high level of heterogeneity observed between the included studies, these results should be interpreted with caution and additional studies are needed.

**Systematic review registration:**

https://www.crd.york.ac.uk/prospero/, identifier: CRD42022316066.

## Introduction

Intracranial atherosclerotic stenosis (ICAS) is one of the most important causes of stroke worldwide and is associated with a high risk of recurrent stroke (1, 2). In the USA, ICAS is present in 7–10% of patients with cerebrovascular disease (3). In China, stroke is the most common cause of death. The CICAS study showed that the prevalence of ICAS was up to 46.6% in patients with ischemic cerebrovascular disease, and patients with ICAS had more severe stroke symptoms and longer hospital stays than those without ICAS (4). Treatment for ICAS includes dual antiplatelet treatment, active management of risk factors, and endovascular therapy (2, 5). Aggressive medical therapy (AMT) is the primary treatment for ICAS (6), which can effectively reduce the recurrence of stroke. However, for some symptomatic ICAS patients with severe stenosis (70–99%), the stroke recurrence rate after receiving AMT remains high (7–9). Percutaneous transluminal angioplasty and stenting (PTAS) has been considered an effective alternative treatment for severe ICAS (5, 10).

The Stenting Versus Aggressive Medical Therapy for Intracranial Arterial Stenosis (SAMMPRIS) trial was a randomized trial comparing AMT alone with AMT plus PTAS with Wingspan stents (7). In 2011, the study found that AMT was superior to PTAS due to the high rates of perioperative complications of PTAS. The 30-day incidence of stroke or death was 14.7% in the stenting group vs. 5.8% in the AMT group. The poor results of SAMMPRIS have raised concerns about the safety of intracranial stenting, but some scholars believe that the design of this trial needs to be improved in areas such as patient enrollment, device selection, physician experience, and antiplatelet therapy testing (11, 12). In later years, some studies suggest that appropriate patient selection and the extensive surgical experience of the surgeons may have contributed to the low perioperative complication rates in PTAS with Wingspan stents (13–15). Currently, the Wingspan stent is the only on-label self-expandable stent (SES) for the treatment of ICAS. Meanwhile, some studies have sought to select other off-label SESs for the treatment of symptomatic ICAS, such as Enterprise, Neuroform EZ, LVIS, and Solitaire AB, which was originally designed to treat wide-necked aneurysms. The results showed that intracranial angioplasty with off-label SES for symptomatic ICAS also has fewer perioperative complications (16–18). It appears that the perioperative safety of PTAS with SES has improved and that PTAS with SES may be an effective alternative treatment for symptomatic ICAS. However, in 2022, another randomized clinical trial China Angioplasty and Stenting for Symptomatic Intracranial Severe Stenosis (CASSISS) (19) found no significant difference between the wingspan group and the AMT group in the risk of stroke or death within 30 days or stroke beyond 30 days through 1 year (wingspan: 8.0% [14/176] vs. AMT: 7.2% [13/181]). The results do not support the addition of PTAS to medical therapy for the treatment of patients with symptomatic severe ICAS.

Therefore, is PTCAS with SES (including the Wingspan stent and off-label SES) safe and effective for the treatment of ICAS across the world? Are the results different between Wingspan stents and off-label SESs? The answers to these questions are still unclear, and it is necessary to verify the safety and effectiveness of SES for the treatment of ICAS. Here, we conducted a systematic review and meta-analysis to determine the perioperative complications and long-term outcomes beyond 30 days of stenting with SES for treating symptomatic ICAS, and to further explore the differences between Wingspan stents and off-label SESs.

## Methods

### Search strategy

This systematic review followed the Preferred Reporting Items for Systematic Reviews and Meta-Analyses (PRISMA) (20). The study was registered in the International Prospective Register of Systematic Reviews (PROSPERO, CRD42022316066). We searched the published literature from the PubMed, EMBASE, Web of Science, Cochrane, and Clinical Trials databases. The following keywords were used: “self expandable stent” “self expandable stents” “self expanding stent” “self expanding stents” “neuroform atlas” “neuroform ez” “enterprise stent” “wingspan” “lvis stent” “solitaire ab” “percutaneous transluminal angioplasty and stenting” “intracranial arteriosclerosis” “intracranial atherosclerotic stenosis” “intracranial atherosclerotic stenosis” “intracranial stenosis” “intracranial stenosis” “intracranial artery stenosis” “intracranial arterial stenosis” “intracranial atheromatous disease” “intracranial atherosclerotic disease” “vertebrobasilar artery” “internal carotid artery” “carotid artery, internal” “basilar artery” “vertebral artery” “middle cerebral artery” “aneurysm” “aneurysms” “coil” “coiling,” keywords were used in both “AND,” “OR” and “NOT” combinations, as described in Supplementary Table 1. The end date of the search was Sept 16, 2022.

### Criteria for considering studies for this review

We included studies that met the following criteria:

1. Patients with symptomatic ICAS (the degree of stenosis was between 50 and 99%) and at least one major intracranial artery (intracranial internal carotid artery, intracranial vertebral artery, middle cerebral artery or basilar artery) was stenotic.
2. PTAS with SES.
3. The study reported one of the following outcomes:
  3.1 Perioperative complications (≤ 30 days after PTAS): transient ischemic attack (TIA), hemorrhagic or ischemic stroke, stroke, death, stroke or death rate.
  3.2 Long-term outcomes (>30 days after PTAS): The rates of TIA beyond 30 days, ischemic stroke beyond 30 days, ischemic stroke or TIA beyond 30 days, death beyond 30 days, ischemic stroke or death beyond 30 days, in-stent restenosis (degree of restenosis ≥50%, ISR), and ischemic stroke beyond 30 days through 1 year.
4. Randomized controlled trials (RCTs), cohort study, case-control study, case series report with sample size >10.

1. We excluded studies based on the following criteria:
2. Patients with symptomatic ICAS related to nonatherosclerotic factors: arterial dissection, moyamoya disease, radiation-induced vasculopathy, etc. Patients with acute occlusion of the cerebral artery.
3. PTAS without SES, or impossible to differentiate SES from non-SES, impossible to differentiate intracranial from extracranial artery stenosis.
4. Case series report with sample size ≤ 10, case report, conference abstracts without full text, duplicate studies, non-English articles.

### Trial selection

Two reviewers (CZ and SJC) independently completed the preliminary screening of the title and abstract of all articles retrieved from the literature search. The assessment of the full text articles was conducted by two reviewers (CZ and SJC), and those that met the eligibility criteria were included. When multiple studies were reported from the same study population, the study with the largest sample size was retained. Disagreements between the two reviewers were resolved by discussion; otherwise, a third reviewer (JZ) was consulted.

### Data extraction

Two review authors (CZ and SJC) independently extracted the data from each eligible study: (1) characteristics of the study: publication time, study location, study design, stent variety; (2) patients: number of patients and lesions, gender, mean age, clinical symptoms, preprocedural and postprocedural mean stenosis rate, technical success rate and mean angiographic or clinic follow-up time; (3) perioperative complications: TIA, hemorrhagic or ischemic stroke, stroke, death, stroke or death rate; and (4) long-term outcomes: the rates of TIA beyond 30 days, ischemic stroke beyond 30 days, ischemic stroke or TIA beyond 30 days, death beyond 30 days, ischemic stroke or death beyond 30 days, ISR, ischemic stroke beyond 30 days through 1 year.

### Quality assessment

Two investigators (CZ and SJC) independently assessed the quality of the included literature, and any conflicts that could not be resolved by discussion were referred to a third author (JZ). RCTs were assessed with items in the Cochrane Handbook (21), while the non-RCTs (cohort studies and case–control studies) were assessed with the Newcastle–Ottawa Scale (22).

### Statistical analysis

All statistical analyses were performed using Stata Statistical Software (version 14.0, Stata Corp, College Station, Texas, USA). The pooled outcomes and 95% confidence interval (CI) of each group were estimated using the metaprop module of STATA (23). Study heterogeneity was evaluated using the Cochrane Q test and I^2^ statistics. Heterogeneity was considered present if the Q test was significant (*P* < 0.10). I^2^ values of 0–25%, 25–50%, 51–75%, and > 75% indicated light, low, moderate, and high heterogeneity, respectively (24). The random-effects model was applied refer to the unavoidable heterogeneity among the included studies. When heterogeneity was present, sensitivity analysis and subgroup analyses were used to explore the source of the heterogeneity. Meta-regression analysis was also conducted to determine factors associated with heterogeneity. *P* < 0.05 were considered statistically significant. Publication bias was assessed by a funnel plot, and asymmetric funnel plots suggested publication bias. The Egger and Begg tests were used to evaluate the publication bias.

## Results

### Search results

**Figure 1:**
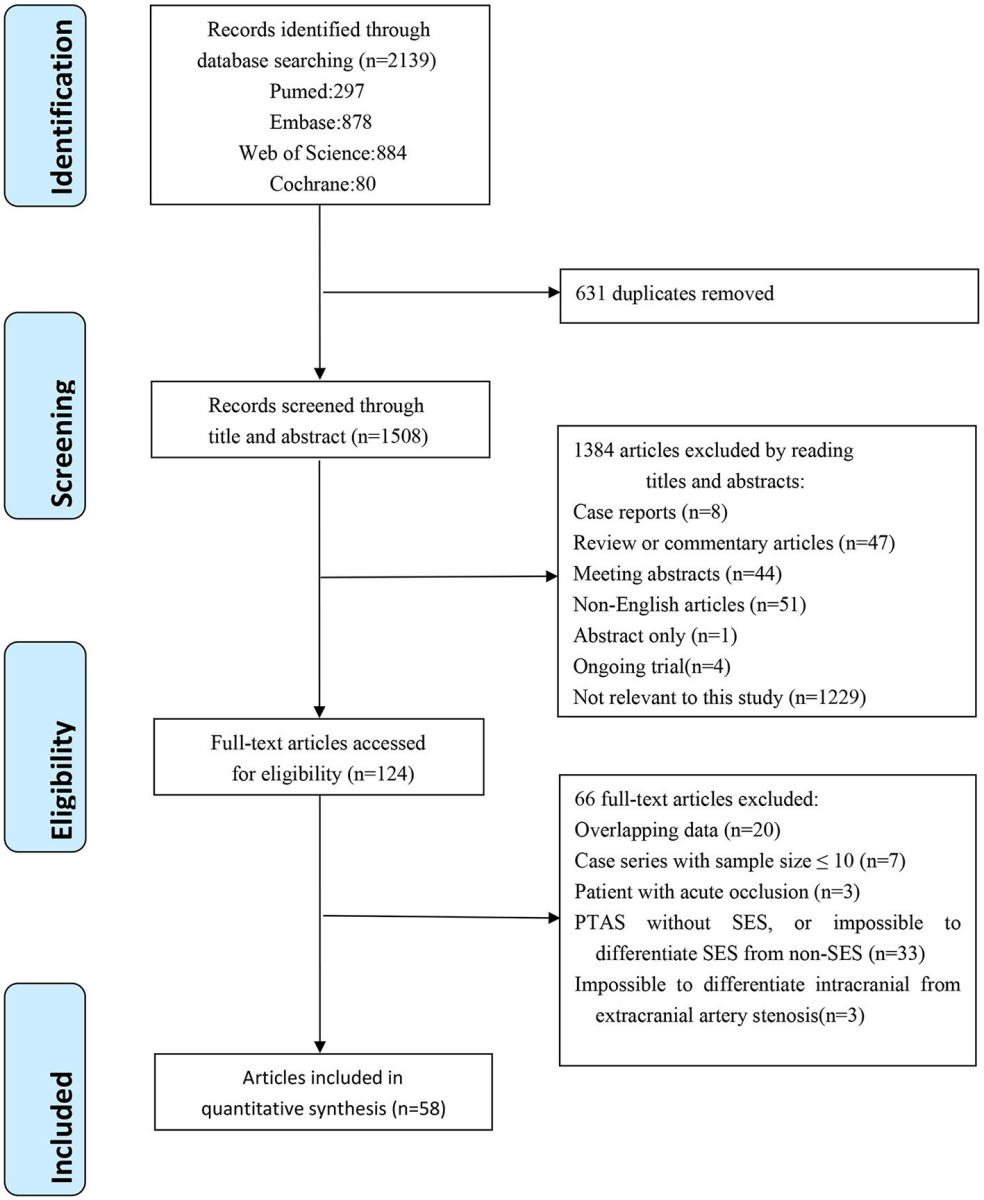
Flowchart of study selection.

The flow diagram of the literature selection process is shown in Figure 1. A total of 2,139 references and abstracts were identified from the electronic database. A total of 631 duplicated articles were removed, 1,384 articles were excluded after the screening of titles and abstracts, 66 articles were excluded after the full-text evaluation, and 58 articles were eligible according to the inclusion criteria.

### Characteristics of included studies

A total of 58 studies with 4362 patients were included in this systematic review. The main characteristics of the included studies are shown in Table 1; Additional details are given in Supplementary Table 2. The publication time of the included studies was between 2005 and 2022. More studies were conducted in east country than in west country (37 vs. 21). Forty-one studies were case series studies, 14 were cohort studies, and three studies, Chimowitz et al. (7), Gao et al. (19), Qureshi et al. (25), were RCTs. Five other RCTs, Coward et al. (26), Miao et al. (27), Compter et al. (28), Zaidat et al. (9), Markus et al. (29), were excluded because we were unable to distinguish SES from non-SES, or intracranial from extracranial artery stenosis. Thirty-six studies used the wingspan stent, 18 studies used the off-label SES (Enterprise, Neuroform EZ, Solitaire AB, LVIS), and four studies used multiple varieties of SES. The mean age of the patients enrolled in all studies ranged from 51.7 to 70.5 years, and the technical success rates ranged from 89 to 100%. The preprocedural mean stenosis ranged from 63 to 92.0%, and the postprocedural mean stenosis ranged from 8.8 to 38.0%. Mean angiographic or clinical follow-up time ranged from 1 to 60 months, with 36 studies having mean follow-up of more than or equal to 12 months.

**Table 1:** Main characteristics of the 58 included studies.

**Study characteristics**	**Category**	**Number of studies**
**Publication time (2005–2022)**		
2005–2013	1	24
2014–2022	2	34
**Study location**		
West country	1	21
East country	2	37
**Study design**		
RCTs or cohort studies	1	17
Case series studies	2	41
**Stent variety**		
Wingspan	1	36
Off-label SES	2	18
SES(wingspan + off-label SES)	3	4
**Mean age (year, range 51.7–70.5)**		
≤ 60	1	24
>60	2	34
**Mean preprocedural stenosis, %(range 65.4–92)**		
≤ 80	1	26
>80	2	30
NA	3	2
**Mean follow-up time (month)**		
≥12	1	36
< 12	2	20
NA	3	2

### Assessment of study quality

Two review authors (CZ and SJC) independently evaluated the quality of each RCT and cohort study based on the Cochrane Handbook or the Newcastle–Ottawa Scale (NOS). Three RCTs were included in our research, and the Cochrane Handbook score implied that they had a low risk of bias. Fourteen cohort studies in our research were evaluated according to the NOS scale; 3 studies scored 8 stars, and 11 studies scored 9 stars. All cohort studies had more than 8 stars and were of high quality. Since there was no control group in the case series studies, no quality evaluation was performed. The specific evaluation details are shown in Supplementary Figure 1.

### Perioperative complications

The summary of perioperative complications after PTAS for patients with symptomatic ICAS is shown in Supplementary Table 3. The pooled rate of each outcome was as follows (Table 2): perioperative TIA, 2.20% (95% CI 1.21–3.39%), with little heterogeneity (I^2^ 20.37%); perioperative hemorrhagic stroke, 1.66% (95% CI 1.15–2.24%), with little heterogeneity (I^2^ 19.45%); perioperative ischemic stroke, 3.34% (95% CI 2.40–4.39%), with moderate heterogeneity (I^2^ 56.75%); perioperative stroke, 6.13% (95% CI 4.86–7.51%), with moderate heterogeneity (I^2^ 62.55%); perioperative death, 0.41% (95% CI 0.18–0.70%), without heterogeneity (I^2^ 0.00%); and perioperative stroke or death, 6.32% (95% CI 5.04–7.72%), with moderate heterogeneity (I^2^ 62.83%). The forest plots of the perioperative complications are shown in Supplementary Figure 2.

**Table 2:** Pooled rate of perioperative complications and long-term outcomes.

**Outcomes**	**Number of studies**	**Size of outcomes**	**Pooled rate (95% CI)**	**Heterogeneity**	**I^2^ statistics**	**Q test**
Perioperative TIA	18	1,262	2.20% (1.21–3.39%)	Little	I^2^ 20.37%	*p* = 0.211
Perioperative hemorrhagic stroke	55	4,360	1.66% (1.15–2.24%)	Little	I^2^ 19.45%	*P* = 0.110
Perioperative ischemic stroke	55	4,360	3.34% (2.40–4.39%)	Moderate	I^2^ 56.71%	*P* = 0.000
Perioperative stroke	57	4,591	6.13% (4.86–7.51%)	Moderate	I^2^ 62.55%	*P* = 0.000
Perioperative death	58	4,632	0.41% (0.18–0.70%)	Without	I^2^ 0.00%	*p* = 0.714
**Perioperative stroke or death**	**58**	**4,632**	**6.32% (5.04–7.72%)**	**Moderate**	**I**^**2**^ **62.83%**	***P*** **=** **0.000**
TIA beyond 30 days	39	2,773	1.18% (0.41–2.21%)	Moderate	I^2^ 63.54%	*P* = 0.000
Ischemic stroke beyond 30 days	43	3,059	2.55% (1.68–3.55%)	Low	I^2^ 42.62%	*p* = 0.002
Ischemic stroke or TIA beyond 30 days	46	3,405	4.23% (2.97–5.65%)	Moderate	I^2^ 66.67%	*p* = 0.000
Death beyond 30 days	37	2,869	0.48% (0.17–0.90%)	Little	I^2^ 4.27%	*p* = 0.396
Ischemic stroke or death beyond 30 days	35	2,778	3.43% (2.20–4.87%)	Moderate	I^2^ 62.01%	*p* = 0.000
ISR	43	2,262	13.33% (10.25–16.70%)	High	I^2^ 76.85%	*p* = 0.000
**Ischemic stroke beyond 30 days through 1 year**	**13**	**1,853**	**2.72% (1.41–4.38%)**	**Moderate**	**I**^**2**^ **66.57%**	***p*** **=** **0.000**

Perioperative stroke or death and ischemic stroke beyond 30 days through 1 year are two important safe and effective indicators of PTAS.

### Long-term outcomes

The summary of long-term outcomes is shown in Supplementary Table 3. The pooled rate of each outcome was as follows (Table 2): TIA beyond 30 days, 1.18% (95% CI 0.41–2.21%), with moderate heterogeneity (I^2^ 63.54%); ischemic stroke beyond 30 days, 2.55% (95% CI 1.68–3.55%), with low heterogeneity (I^2^ 42.62%); ischemic stroke or TIA beyond 30 days, 4.23% (95% CI 2.97–5.65%), with moderate heterogeneity (I^2^ 66.67%); death beyond 30 days, 0.48% (95% CI 0.17–0.90%), with little heterogeneity (I^2^ 4.27%); ischemic stroke or death beyond 30 days, 3.43% (95% CI 2.20–4.87%), with moderate heterogeneity (I^2^ 62.01%); ISR, 13.33% (95% CI 10.25–16.70%), with high heterogeneity (I^2^ 76.85%); ischemic stroke beyond 30 days through 1 year, 2.72% (95% CI 1.41–4.38%), with moderate heterogeneity (I^2^ 66.57%). The forest plots of the long-term outcomes beyond 30 days are shown in Supplementary Figure 2.

### Subgroup analyses

First, subgroup analyses were performed by publication time, namely, 2005–2013 (most of the studies were conducted before the SAMMPRIS trial) or 2014–2022 (most of the studies were conducted after the SAMMPRIS trial) subgroup. Results showed that the 2014–2022 subgroup had lower perioperative complications than the 2005–2013 subgroup, and there was no significant difference in pooled rates of long-term outcomes (Table 3). Second, subgroup analyses were conducted by stent variety, namely, Wingspan or off-label SES subgroup. Results showed that the off-label SES subgroup had better long-term outcomes than the Wingspan subgroup in terms of long-term ischemic stroke beyond 30 days, ischemic stroke or TIA beyond 30 days, ischemic stroke or death beyond 30 days, and ISR (Table 4).

**Table 3:** Summary of subgroup analyses based on publication time.

**Outcomes**	**Subgroup**	**Number of studies**	**Size of subgroup**	**Pooled rate (95% CI)**	**Heterogeneity between subgroups (*P* value)**
Perioperative TIA	2005–2013	8	548	2.43% (1.13–4.08%)	*P* = 0.874
	2014–2022	7	394	1.96% (0.52–4.05%)	
Perioperative hemorrhagic stroke	2005–2013	20	1,299	2.05% (1.13–3.16%)	***P*** **=** **0.046**
	2014–2022	32	2,666	1.24% (0.72–1.87%)	
Perioperative ischemic stroke	2005–2013	22	1,504	4.67% (2.90–6.76%)	***P*** **=** **0.023**
	2014–2022	33	2,856	2.60% (1.68–3.66%)	
Perioperative stroke	2005–2013	24	1,735	8.47% (6.03–11.23%)	***P*** **=** **0.003**
	2014–2022	33	2,856	4.69% (3.56–5.93%)	
Perioperative death	2005–2013	22	1,530	0.69% (0.21–1.35%)	***P*** **=** **0.021**
	2014–2022	32	2,603	0.18% (0.01–0.50%)	
Perioperative stroke or death	2005–2013	24	1,735	8.89% (6.42–11.68%)	***P*** **=** **0.001**
	2014–2022	34	2,897	4.77% (3.65–6.01%)	
TIA beyond 30 days	2005–2013	16	1,066	1.29% (0.13–3.19%)	*P* = 0.846
	2014–2022	23	1,707	1.11% (0.21–2.45%)	
Ischemic stroke beyond 30 days	2005–2013	17	1,073	1.74% (0.51–3.44%)	*P* = 0.332
	2014–2022	24	1,736	3.03% (1.92–4.33%)	
Ischemic stroke or TIA beyond 30 days	2005–2013	20	1,331	4.20% (2.31–6.51%)	*P* = 0.885
	2014–2022	26	2,074	4.25% (2.61–6.20%)	
Death beyond 30 days	2005–2013	15	1,011	0.02% (0.00–0.42%)	*p* = 0.052
	2014–2022	19	1,567	0.95% (0.46–1.57%)	
Ischemic stroke or death beyond 30 days	2005–2013	15	1,011	1.95% (0.43–4.18%)	*P* = 0.129
	2014–2022	29	1,767	4.55% (2.92–6.46%)	
ISR	2005–2013	18	789	16.98% (11.44–23.26%)	*P* = 0.076
	2014–2022	25	1,373	11.17% (7.71–15.11%)	
Ischemic stroke beyond 30 days through 1 year	2005–2013	5	535	3.01% (0.31–7.64%)	*P* = 0.679
	2014–2022	8	1,318	2.35% (1.25–3.73%)	

The 2014–2022 subgroup had lower perioperative complications than the 2005–2013 subgroup in terms of perioperative hemorrhagic stroke, perioperative ischemic stroke, perioperative stroke, perioperative death, and perioperative stroke or death (*P* value < 0.05).

**Table 4:** Summary of subgroup analyses based on stent variety.

**Outcomes**	**Subgroup**	**Number of studies**	**Size of subgroup**	**Pooled rate (95% CI)**	**Heterogeneity between subgroups (*P* value)**
Perioperative TIA	Wingspan	11	704	2.39% (1.21–3.86%)	*P* = 0.536
	Off-label SES	5	314	1.96% (0.52–4.05%)	
Perioperative hemorrhagic stroke	Wingspan	34	2,832	1.75% (1.20–2.38%)	*P* = 0.051
	Off-label SES	18	1,133	0.89% (0.29–1.71%)	
Perioperative ischemic stroke	Wingspan	34	2,832	3.26% (2.13–4.57%)	*P* = 0.547
	Off-label SES	18	1,133	2.81% (1.52–4.39%)	
Perioperative stroke	Wingspan	36	3,063	6.49% (4.87–8.28%)	*P* = 0.063
	Off-label SES	18	1,133	4.34% (2.84–6.08%)	
Perioperative death	Wingspan	36	3,063	0.41% (0.14–0.79%)	*P* = 0.230
	Off-label SES	18	1,070	0.12% (0.00–0.63%)	
Perioperative stroke or death	Wingspan	36	3,063	6.71% (5.05–8.55%)	*P* = 0.051
	Off-label SES	18	1,133	4.38% (2.85–6.17%)	
TIA beyond 30 days	Wingspan	24	1,918	1.56% (0.43–3.18%)	*P* = 0.289
	Off-label SES	14	665	0.54% (0.00–1.86%)	
Ischemic stroke beyond 30 days	Wingspan	28	2,204	3.67% (2.82–4.61%)	***P*** **=** **0.002**
	Off-label SES	14	665	1.24% (0.35–2.48%)	
Ischemic stroke or TIA beyond 30 days	Wingspan	29	2,335	5.32% (3.59–7.29%)	***P*** **=** **0.018**
	Off-label SES	15	839	2.29% (0.85–4.20%)	
Death beyond 30 days	Wingspan	25	2,130	0.68% (0.27–1.21%)	*p* = 0.179
	Off-label SES	10	508	0.10% (0.00–0.90%)	
Ischemic stroke or death beyond 30 days	Wingspan	24	2,080	4.35% (2.76–6.23%)	***P*** **=** **0.028**
	Off-label SES	10	508	1.66% (0.42–3.44%)	
ISR	Wingspan	26	1,289	15.73% (11.31–20.66%)	***P*** **=** **0.036**
	Off-label SES	15	780	8.84% (4.80–13.79%)	

The off-label SES subgroup had better long-term outcomes than the Wingspan subgroup in terms of long-term ischemic stroke beyond 30 days, ischemic stroke or TIA beyond 30 days, ischemic stroke or death beyond 30 days, and ISR (*P* value < 0.05).

### Meta-regression analyses

The results of univariate and multivariate meta-regression analyses indicated that differences in publication time, stent variety, study location, study design, mean age, and preprocedural stenosis, did not account for the observed heterogeneity in this review (Tables 5, 6, Supplementary Table 4).

**Table 5:** Univariate meta-regression analyses based on publication time.

**Outcomes**	**Number of studies**	**Size of outcomes**	**Exp(b)**	**(95% CI)**	***p* value**
Perioperative TIA	15	942	1.004	0.8710–1.1573	0.953
Perioperative hemorrhagic stroke	51	3,957	0.9886	0.9237–1.0580	0.735
Perioperative ischemic stroke	50	3,821	0.9793	0.9146–1.0487	0.542
Perioperative stroke	52	4,052	0.9643	0.9037–1.0289	0.266
perioperative death	54	4,133	0.991	0.9292–1.0570	0.780
Perioperative stroke or death	58	4,632	0.9527	0.8966–1.0122	0.115
TIA beyond 30 days	37	2523	1.0011	0.9227–1.0861	0.979
Ischemic stroke beyond 30 days	41	2,809	1.0083	0.9324–1.0904	0.831
Ischemic stroke or TIA beyond 30 days	43	3,114	0.9998	0.9295–1.0754	0.996
Death beyond 30 days	34	2,578	1.0126	0.9330–1.0991	0.757
Ischemic stroke or death beyond 30 days	33	2,528	1.0217	0.9408–1.1096	0.599
ISR	40	2,009	0.9412	0.8585–1.0319	0.19
Ischemic stroke beyond 30 days through 1 year	13	1,853	0.9746	0.8708–1.0908	0.625

Univariate meta-regression analysis was adjusted by publication time (2005–2013 subgroup or 2014–2022 subgroup).

**Table 6:** Univariate meta-regression analyses based on stent variety.

**Outcomes**	**Number of studies**	**Size of outcomes**	**Exp(b)**	**(95% CI)**	***p* value**
Perioperative TIA	15	942	0.9981	0.8494–1.1728	0.980
perioperative hemorrhagic stroke	51	3,957	0.9887	0.9214–1.0608	0.746
Perioperative ischemic stroke	50	3,821	0.9917	0.9209–1.0678	0.821
Perioperative stroke	52	4,052	0.9774	0.9084–1.0516	0.533
Perioperative death	54	4,133	0.9932	0.9252–1.0663	0.848
Perioperative stroke or death	58	4,632	0.9733	0.9091–1.0420	0.430
TIA beyond 30 days	37	2,523	0.9804	0.8922–1.0772	0.672
Ischemic stroke beyond 30 days	41	2,809	0.9738	0.8878–1.0680	0.564
Ischemic stroke or TIA beyond 30 days	43	3,114	0.9622	0.8853–1.0457	0.355
Death beyond 30 days	34	2,578	0.9864	0.8877–1.0960	0.793
Ischemic stroke or death beyond 30 days	33	2,528	0.9636	0.8669–1.0710	0.480
ISR	40	2,009	0.9412	0.8570–1.0337	0.198

Univariate meta-regression analysis was adjusted by stent variety (wingspan subgroup or off-label SES subgroup).

### Publication bias

Publication bias was assessed by funnel plots and Egger's or Begg's tests. As shown in Supplementary Figure 3, both funnel plots were symmetric, and the results of Egger's or Begg's tests (*p* > 0.05) indicated that there was no publication bias.

### Sensitivity analyses

We performed a leave-one-out sensitivity analysis in each group with moderate or high heterogeneity by using Stata 14.0. For all groups with moderate or high heterogeneity, the direction and magnitude of the pooled rate did not change obviously with the removal of each study, implying that the conclusions of the meta-analysis were stable. The diagrams of the sensitivity analyses are shown in Supplementary Figure 4.

## Discussion

This systematic review and meta-analysis included 58 studies and 4,362 patients from 9 countries and assessed the perioperative complications and long-term outcomes of PTAS with SES in treating patients with symptomatic ICAS. The review showed that (1) the pooled rate of perioperative stroke or death was 6.32%; the pooled rate of ischemic stroke beyond 30 days through 1 year was 2.72%; the pooled rate of ischemic stroke or TIA beyond 30 days was 4.23%, the pooled rate of ISR was 13.33% (Table 2). and (2) Perioperative complications differed between the 2014–2022 and 2005–2013 subgroups, as did long-term outcomes between the off-label SES and Wingspan subgroups (Tables 3, 4).

The Wingspan stent is the only on-label SES for the treatment of symptomatic ICAS, having been approved by the FDA in 2005 (30). The approval was based on the initial trial conducted by Bose et al., which showed that the 30-day perioperative stroke or death rate after stenting with Wingspan was 4.5% (11). Two subsequent multicentre studies were conducted in the United States, and the results indicated that the perioperative stroke or death rates were 6.4 and 9.3%, respectively (31, 32). The long-term results were reported in two manuscripts. Wolfe et al. (33) reported that the stroke or death rate with a 14-month follow-up time was 10%, and Fiorella et al. (34) showed that the stroke or death rate with a 12-month follow-up time was 15.7%. Compared to the poor outcomes of the WASID trial, which showed that the stroke or death rate with a 12-month follow-up time was 22.1% in the aspirin group and 21.8% in the warfarin group (35), PTAS with Wingspan stents may be a potentially effective treatment for symptomatic ICAS.

However, the first RCT, SAMMPRIS, demonstrated a higher stroke or death rate in the Wingspan group than in the AMT group (7), increasing anxiety about the perioperative safety of PTAS. In response to the high perioperative complication rates, researchers have sought to improve the stenting safety by selecting appropriate patients, engaging experienced physicians, or using off-label SES. The results showed that the perioperative stroke or death rate of PTAS with SES ranged from 1.5 to 12.5%, and the ischemic stroke or death rate beyond 30 days ranged from 0 to 9.0% (14–18). However, most studies were case series or cohort studies and lack RCT. The CASSISS (19) trial is the latest RCT conducted in China. Results showed that the risk of stroke or death within 30 days was 5.1% in the wingspan group vs. 2.2% in the AMT group, with no statistical difference between the two groups. In our systematic review, the pooled rate of perioperative stroke or death with SES was 6.32%, and subgroup analyses indicated that the perioperative complications of stenting have reduced in the past 17 years (Table 3). However, compared to the results of the SAMMPRIS and CASSISS trials, the pooled rate of perioperative stroke or death with SES in our review is still higher than that with AMT in both RCTs (6.32 vs. 5.8%, 6.32 vs. 2.2%, respectively), suggesting that the safety of PTAS with SES still needs to be improved. The rate of ischemic stroke beyond 30 days through 1 year is an important indicator of the long-term outcomes for PTAS. In our systematic review, the pooled rate of ischemic stroke beyond 30 days through 1 year with SES was 2.72%, compared to 6.4% with AMT in the SAMMPRIS trial and 5.0% in the CASSISS trial. It appears that PTAS with SES may have a lower risk of stroke beyond 30 days through 1 year, but it is ambiguous whether stenting with SES may have better long-term outcomes than AMT in treating symptomatic ICAS, given the varying study designs and other confounding factors, and additional studies are needed to determine.

Off-label SES is more flexible than Wingspan and exerts a lower outward radial force (18), which may contribute to the reduction of ISR and improve the long-term effects of stenting. Subgroup analyses of our review showed that the pooled rates of ischemic stroke or TIA beyond 30 days, and ISR were different between the off-label SES and Wingspan subgroup (2.29 vs. 5.32%; 8.84 vs. 15.73%, Table 4). Further studies are needed to determine whether stenting with off-label SES may have better long-term outcomes than stenting with Wingspan in treating symptomatic ICAS.

Estimates of perioperative complications and long-term outcomes for PTAS with SES from different countries were pooled in this meta-analysis and, as expected, high heterogeneity between studies was found. We performed sensitivity analyses, subgroup analyses, univariate meta-regression analyses and multivariate meta-regression analyses to explore the sources of apparent heterogeneity among the included studies (Supplementary Figure 4, Supplementary Table 4, Tables 4–6). Multivariate meta-regression analyses included the following covariates: publication time, study location, study design, stent variety, mean age, and preprocedural stenosis. Subgroup analyses showed that the 2014-2022 subgroup had lower perioperative complications than the 2005–2013 subgroup, and that the off-label SES subgroup had better long-term outcomes than the Wingspan subgroup. These suggest that publication time and stent variety may be related to the significant heterogeneity. Meanwhile, we speculate that the heterogeneity among the included studies could be explained by factors such as differences in patient inclusion criteria, surgeon's experience, device selection, perioperative management, and patient long-term management (13, 14, 36). In the future, more RCTs and well-designed studies are needed.

## Limitations

First, this systematic review included RCTs, cohort studies and case series studies. Most studies (71%) were prospective or retrospective case series studies and lacked appropriate control groups for comparison with the stenting groups, and only three RCTs were included, which is a major shortcoming of our systematic review. Second, the sample size of the included studies varied widely, ranging from 8 to 433, with 76% of the studies having a sample size of fewer than 100. Third, the follow-up time of each study varied, which may confuse the evaluation of long-term results. Last, the publication time span of the included studies was wide, which might be partially responsible for the heterogeneity in the results.

## Conclusion

This systematic review provides a summary of perioperative and long-term outcomes of PTAS with SES in the treatment of symptomatic ICAS. The results suggest that the perioperative complications of PTAS with SES have been reduced, but the risk of perioperative stroke or death is still higher than that of AMT, and additional studies are needed to determine whether it has better long-term outcomes than AMT. Perioperative complications varied between the 2014–2022 and 2005–2013 subgroups, as did long-term outcomes between the off-label SES and Wingspan subgroups. Given the high level of heterogeneity observed between the included studies, these results should be interpreted with caution and additional studies are needed.

## Data availability statement

The original contributions presented in the study are included in the article/Supplementary material, further inquiries can be directed to the corresponding author.

## Author contributions

CQ: guarantor of integrity of entire study. CZ, SC, JZ, and CQ: study concepts and study design. CZ, YL, and LP: data extraction and analysis. CZ: manuscript drafting. CZ, SC, and JZ: statistical analysis. All authors: data acquisition, manuscript revision for important intellectual content, approval of final version of submitted manuscript, agrees to ensure any questions related to the work are appropriately resolved, and manuscript editing.
