# Supplementary material for: Intracranial angioplasty with a self-expandable stent for intracranial atherosclerotic stenosis: Systematic review and meta-analysis
Source: Front Neurol. 2023 Jan 9;13:1074228. doi: 10.3389/fneur.2022.1074228 (PMC9870250; doi:10.3389/fneur.2022.1074228)
Supplement: Supplementary file 1 [file Data_Sheet_1.doc]

**Table 1. Search strategies** （End date Sept 16, 2022)

| Database | Step | Search algorithm | Items found |
| --- | --- | --- | --- |
| Pubmed | #1  #2  #3  #4  #5 | "self expandable stent"[Title/Abstract] OR "self expandable stents "[Title/Abstract] "self expanding stent"[Title/Abstract] OR "self expanding stents "[Title/Abstract] OR "neuroform atlas"[Title/Abstract] OR "neuroform ez"[Title/Abstract] OR "enterprise stent"[Title/Abstract] OR "wingspan"[Title/Abstract] OR "lvis stent"[Title/Abstract] OR "solitaire ab"[Title/Abstract] OR "percutaneous transluminal angioplasty and stenting"[Title/Abstract]  "intracranial arteriosclerosis"[MeSH Terms] OR "intracranial atherosclerotic stenosis"[Title/Abstract] OR "intracranial atherosclerotic stenoses"[Title/Abstract] OR "intracranial stenosis"[Title/Abstract] OR "intracranial stenoses"[Title/Abstract] OR "intracranial artery stenosis"[Title/Abstract] OR "intracranial arterial stenosis"[Title/Abstract] OR "intracranial atheromatous disease"[Title/Abstract] OR "intracranial atherosclerotic disease"[Title/Abstract] OR "vertebrobasilar artery"[Title/Abstract] OR "internal carotid artery"[Title/Abstract] OR "carotid artery, internal"[MeSH Terms] OR "basilar artery"[MeSH Terms] OR "vertebral artery"[MeSH Terms] OR "middle cerebral artery"[MeSH Terms]  "aneurysm"[Title] OR "aneurysms"[Title] OR "coil"[Title] OR "coiling"[Title]  #1 AND #2  #4 NOT #3 | 1379  60913  93371  354  297 |
| Embase | #1  #2  #3  #4  #5 | ‘self expandable stent’:ab,ti OR ‘self expandable stents’:ab,ti OR ‘self expanding stent’/exp OR ‘self expanding stents’:ab,ti OR 'wingspan':ab,ti OR 'enterprise stent':ab,ti OR 'neuroform ez':ab,ti OR 'neuroform atlas':ab,ti OR 'lvis stent':ab,ti OR 'solitaire ab':ab,ti OR 'percutaneous transluminal angioplasty and stenting':ab,ti  'intracranial arteriosclerosis':ab,ti OR 'intracranial atherosclerotic stenosis':ab,ti OR 'intracranial atherosclerotic stenoses':ab,ti OR 'intracranial stenosis':ab,ti OR 'intracranial stenoses':ab,ti OR 'intracranial artery stenosis':ab,ti OR 'intracranial arterial stenosis':ab,ti OR 'intracranial atheromatous disease':ab,ti OR 'intracranial atherosclerotic disease':ab,ti OR 'vertebrobasilar artery':ab,ti OR 'internal carotid artery':ab,ti OR 'internal carotid artery'/exp OR 'basilar artery'/exp OR 'vertebral artery'/exp OR 'middle cerebral artery'/exp  aneurysm:ti OR aneurysms:ti OR coil:ti OR coiling:ti  #1 AND #2  #4 NOT #3 | 13126  99137  111310  1209  878 |
| Web of science | #1  #2  #3  #4  #5 | TS= (intracranial arteriosclerosis OR intracranial atherosclerotic stenosis OR intracranial atherosclerotic stenoses OR intracranial stenosis OR intracranial stenoses OR intracranial artery stenosis OR intracranial arterial stenosis OR intracranial atheromatous disease OR intracranial atherosclerotic disease OR vertebrobasilar artery OR internal carotid artery OR carotid artery, internal OR basilar artery OR vertebral artery OR middle cerebral artery)  TS= (self expanding stents OR self expanding stent OR self expandable stents OR self expandable stent OR wingspan OR Enterprise stent OR neuroform ez OR neuroform atlas OR lvis stent OR solitaire ab OR percutaneous transluminal angioplasty and stenting)  TI=(aneurysm or aneurysms or coil or coiling)  #1 AND #2  #4 NOT #3 | 77538  10798  101312  1158  884 |
| Cochrane | #1  #2  #3  #4  #5  #6  #7  #8  #9  #10  #11 | (self expandable stent):ab,ti,kw OR (self expandable stents):ab,ti,kw OR (self expanding stent):ab,ti,kw OR (self expanding stents):ab,ti,kw OR (neuroform atlas):ab,ti,kw OR (neuroform ez):ab,ti,kw OR (enterprise stent):ab,ti,kw OR (wingspan):ab,ti,kw OR (lvis stent):ab,ti,kw OR (solitaire ab):ab,ti,kw OR (percutaneous transluminal angioplasty and stenting):ab,ti,kw  MeSH descriptor: [intracranial arteriosclerosis] explode all trees  MeSH descriptor: [carotid artery, internal] explode all trees  MeSH descriptor: [basilar artery] explode all trees  MeSH descriptor: [vertebral artery] explode all trees  MeSH descriptor: [middle cerebral artery] explode all trees  (intracranial atherosclerotic stenosis):ab,ti,kw OR (intracranial atherosclerotic stenoses):ab,ti,kw OR (intracranial stenosis):ab,ti,kw OR (intracranial stenoses):ab,ti,kw OR (intracranial artery stenosis):ab,ti,kw OR (intracranial arterial stenosis):ab,ti,kw OR (ntracranial atherosclerotic disease):ab,ti,kw OR (intracranial atheromatous disease):ab,ti,kw OR (vertebrobasilar artery):ab,ti,kw OR (internal carotid artery):ab,ti,kw  #2 OR #3 OR #4 OR #5 OR #6 OR #7  #1 AND #8  (aneurysm):ti OR (aneurysms):ti OR (coil):ti OR (coiling):ti  #9 NOT #10 | 1253  488  214  32  71  273  1933  2643  84  2316  80 |

**Table S2 Characteristics of included studies**

| **Characteristics of included studies** | | | | | | | | | | | | | |
| --- | --- | --- | --- | --- | --- | --- | --- | --- | --- | --- | --- | --- | --- |
| **Study** | **Publication time** | **Study location** | **Study design** | **Stent variety** | **Patients/lesions** | **Male/female  ratio** | **Age (year) (mean ± SD)** | **Stroke/TIA ratio** | **Lesion site  AC/PC** | **Preprocedural stenosis rate (%) (mean ± SD)** | **Postprocedural stenosis rate (%)(mean ± SD** | **Technical success rate** | **Mean follow-up time (months）** |
| Henkes2005 | 2005 | Germany | Case series | Wingspan | 15/15 | 10/5 | 64 | 3/12 | 89/120 | 72 | 38 | 100% | NA |
| Bose2007 | 2007 | USA | Case series | Wingspan | 45/45 | 33/12 | 66 | 42/3 | 25/19 | 74.9±9.8 | 31.9±13.6 | 97.8% | 12 |
| Fiorella2007 | 2007 | USA | Case series | Wingspan | 78/82 | 45/33 | 63.6 | 48/28 | 20/10 | 74.6±13.9 | 27.2±16.7 | 98.8% | 1 |
| Zaidat2008 | 2008 | USA | Case series | Wingspan | 129/129 | 71/58 | 64.2 ± 12.4 | 79/37 | 14/48 | 82% ± 9 | 20% ± 16 | 96.70% | 6 |
| Wolfe2009 | 2009 | USA | Case series | Wingspan | 51/51 | 30/21 | 63 | 40/9 | 7/13 | 73 | 21 | 98% | 14.6 |
| Costalat2010 | 2010 | France | Cohort study | Wingspan | 19/19 | 14/5 | 61.8±14.5 | 12/7 | 54/16 | 71.4 | NA | 100% | 15.8 |
| Al-Ali2011 | 2011 | USA | Cohort study | Wingspan | 65/73 | 37/28 | 66.1 | 42/23 | 29/39 | 78 | NA | 94.40% | 26 |
| Chimowitz2011 | 2011 | USA | RCT | Wingspan | 224/224 | 127/97 | 61.0±10.7 | 142/82 | 0/67 | 80±7 | NA | 93.30% | 11.9 |
| Costalat2011 | 2011 | France | Case series | Wingspan | 60/63 | 40/20 | 65.35 ± 9 | 45/15 | 54/76 | 80.2% ± 12.2 | NA | 95.2% | 12 |
| Fiorella2011 | 2011 | USA | Case series | Wingspan | 158/168 | 95/63 | 62.7 | 90/NA | 47/58 | 75.2% | NA | NA | 14.2 |
| Guo2011 | 2011 | China | Case series | Wingspan | 53/53 | 34/19 | 58±18.5 | NA | 25/21 | 76.5 ± 15.4 | 18.2 ± 11.3 | 98.10% | 6 |
| Jiang2011 | 2011 | China | Case series | Wingspan | 100/105 | 87/13 | 53.2±9.2 | 75/30 | 26/46 | 79.0± 7.6 | 25.1±12.3 | 99.10% | 21.4 |
| Li2011 | 2011 | China | Case series | Wingspan | 47/48 | 34/13 | 51.74 ±10.92 | 15/32 | 77/0 | 74.27 ±10.46 | 15.44 ± 11.16 | 100% | 12.92 |
| Yue2011 | 2011 | China | Cohort study | Wingspan | 28/28 | 22/6 | 55.1 ± 10.1 | 16/12 | 113/0 | 83.2% ± 9.4 | 14.4% ± 14.6 | 100% | 14.5 |
| Kurre2012 | 2012 | Germany | Cohort study | SES | 151/155 | 105/46 | 69 | 102/49 | 3/7 | 81 | NA | 98.70% | 1 |
| Lee2012 | 2012 | South Korea | Case series | Wingspan | 19/19 | 12/7 | 65 | 11/8 | 22/9 | 63 | NA | 100% | 7 |
| Li2012 | 2012 | China | Case series | Wingspan | 30/31 | 25/5 | 59.00±8.07 | 18/12 | 35/0 | 82.28±8.02 | 11.18±7.28 | 100% | 17.81 |
| Vajda 2012 | 2012 | Germany | Case series | Enterprise | 189/209 | 132/57 | 64 | 93/83 | 29/15 | 65.4±0.8 | 25.1±1.0 | 99.5% | 6.9 |
| Gandini2013 | 2013 | Italy | Case series | Wingspan | 21/21 | 12/9 | 70.5 | 14/7 | 17/15 | 84 | 17 | 100% | 19.5 |
| Qureshi2013 | 2013 | USA | RCT | Wingspan | 8/8 | 6/2 | 63.88 | 5/3 | 5/3 | 80.25 | 25 | 100% | 8.1 |
| Rohde2013 | 2013 | Germany | Cohort study | SES | 54/54 | 34/20 | 64 ±9 | 37/17 | 6/9 | 85± 10 | 19 ± 14 | 96.30% | 1 |
| Samaniego2013 | 2013 | USA | Case series | Wingspan | 45/46 | 28/17 | 64.9 | 32/13 | 23/22 | Mean>75 | NA | NA | 10.6 |
| Shin2013 | 2013 | South Korea | Case series | Wingspan | 77/79 | 43/44 | 61.8 ± 10.2 | NA | 55/24 | 79.9 ± 8.4 | 25 ± 14.3 | 97.50% | 18.9 |
| Zhang2013 | 2013 | China | Case series | Wingspan | 61/61 | 48/13 | 57.8±9.5 | 34/27 | 54/28 | 76.8±6.0 | 12.0%±11.5 | 98.40% | 23.5 |
| Yu2014 | 2014 | China | Case series | Wingspan | 95/95 | 68/27 | 64.7±10.6 | 41/54 | 76/53 | 75.5±10.3 | NA | 93.70% | 38.9 |
| Feng 2015 | 2015 | China | Case series | Enterprise | 44/44 | 32/12 | 60:45 ± 9:07 | 26/18 | 29/22 | 79:32 ± 8:18 | 14.9 ± 12.3 | 100% | 25.6 |
| Li2015 | 2015 | China | Case series | Wingspan | 433/433 | 299/134 | 57.3 ± 11.6 | 203/230 | NA | 82.3 ± 7.6 | 16.6 ± 6.6 | 99.10% | 29.6 |
| Miao2015 | 2015 | China | Cohort study | Wingspan | 38/38 | 33/5 | 56 | 19/19 | NA | 82.11 | NA | 100% | 1 |
| Yin2015 | 2015 | China | Cohort study | Wingspan | 48/48 | 36/12 | 59.2±10.7 | 32/16 | 36/12 | 81.0±8.0 | NA | 100.00% | 32.5 |
| Duan2016 | 2016 | China | Case series | Solitaire AB | 44/44 | 31/13 | 62.50±11.72 | 19/25 | 53/0 | 80.32±8.11 | 15.00±12.94 | 100% | 25.5 |
| Gao2016 | 2016 | China | Case series | Wingspan | 100/100 | 73/27 | 56 | NA | 60/45 | 82.7 ± 8.9 | 13.5 ±110.2 | 100% | NA |
| Lee 2016 | 2016 | China | Case series | Enterprise | 24/30 | 20/4 | 61.8±10.3 | NA | 48/0 | 81±11.3 | 18±6.8 | 100% | 15.8 |
| Wang 2016 | 2016 | China | Case series | Enterprise | 60/62 | 42/18 | 56.8±8.0 | 22/38 | 17/2 | 76.3±12.7 | 22.8± 4.8 | 100% | 6.2 |
| Yeo2016 | 2016 | Singapore | Case series | Wingspan | 50/50 | 11/39 | 64.8±11.5 | 32/18 | 0/31 | 76.5±13.1 | 19.8±13.8 | 100% | 6 |
| Zhao2016 | 2016 | China | Case series | Wingspan | 278/278 | 228/50 | 60.1 ± 10.7 | 143/135 | 19/2 | 82.5 ± 7.9 | 9.0 ± 3.2 | 99.60% | 21.4 |
| Dong 2018 | 2018 | China | Case series | Enterprise | 20/20 | 15/5 | 57.20±9.25 | 20/0 | NA | 77.45±8.44 | 24.89±16.61 | 100% | 13.2 |
| Du 2018 | 2018 | China | Case series | Neuroform ez | 45/46 | 29/16 | 65±10.8 | 29/16 | 61/0 | 80.7±7.3 | 20.7±13.7 | 100% | 8.8 |
| Gruber2018 | 2018 | Switzerland | Cohort study | Wingspan | 11/11 | 5/6 | 67 | NA | 77/18 | 80 | 10 | 100% | 10 |
| Huang 2019 | 2019 | China | Case series | Enterprise | 68/70 | 46/22 | 59.43±9.74 | 65/3 | 254/179 | NA | NA | 100% | 1 |
| Li2019 | 2019 | China | Cohort study | Wingspan | 55/55 | 39/16 | 54.4 ± 10.4 | 33/22 | 55/45 | 85.4 ± 7.73 | 8.82 ± 7.76 | 100% | 12 |
| Salik 2019 | 2019 | Turkey | Case series | Enterprise | 68/68 | 56/12 | 62 ± 7 | 61/7 | 26/24 | 92 ± 6 | 12 ± 10 | 99% | 22 |
| Xu 2019 | 2019 | China | Case series | Neuroform ez | 71/72 | 54/17 | 58.9±8.2 | 52/19 | 278/0 | 84.2%±9.1 | 16.9%±10.2 | 100% | 1 |
| Zhang2019 | 2019 | China | Cohort study | Wingspan | 69/69 | 58/11 | 58.67 ± 9.52 | 39/30 | 102/55 | 84.17 ± 7.47 | 13.39 ± 8.64 | 100% | 12 |
| Alexander2020 | 2020 | USA | Case series | Wingspan | 152/157 | 81/71 | 61.89±10.52 | 152/0 | 76/19 | 83.18±8.26 | 28.34±16.90 | 100% | 12 |
| Cao2020 | 2021 | China | Case series | Solitaire AB | 32/32 | 25/7 | 57.34± 8.47 | 17/15 | 9/10 | Mean>80 | 13.44±10.66 | 100% | 24.1 |
| Li 2020 | 2020 | China | Case series | Enterprise | 67/67 | 57/10 | 57 ± 8 | NA | 46/27 | 82 ± 9 | 17 ± 10 | 100% | 56 |
| Meyer2020 | 2020 | Germany | Case series | Acclino®(flex) | 76/76 | 51/25 | 69 | 54/22 | 42/34 | NA | NA | 100.00% | 3.6 |
| Park2020 | 2020 | South Korea | Cohort study | Wingspan | 95/95 | 64/31 | 65.5±8.9 | 53/42 | 8/30 | 76.8±6.1 | 7.5±13.4 | 98.90% | 34.9 |
| Tian2020 | 2020 | China | Cohort study | Wingspan | 51/51 | 46/5 | 65 | 20/31 | 28/0 | 77 | 10 | 100% | 40 |
| Cui 2021 | 2021 | China | Case series | Enterprise | 130/130 | 86/44 | 59.2±8.5 | 79/51 | 0/69 | 82.9±8.9 | 15.1±8.4 | 100% | 27.2 |
| Shen2021 | 2021 | China | Cohort study | SES | 41/41 | 21/20 | 60.3 | NA | 55/0 | 86.0±6.2 | 13.5±7.8 | 100% | 12 |
| Sun 2021 | 2021 | China | Case series | Enterprise | 104/105 | 60/44 | 58.61 ± 9.32 | 83/21 | 0/51 | 87.13 ±7.80 | 27:31 ± 8:89 | 100% | 1 |
| Tang2021 | 2021 | China | Case series | Lvis | 31/31 | 21/10 | 58±9.7 | 17/14 | 6/5 | 85.6 ± 9.4 | 11.2 ± 11.8 | 100% | 15 |
| Wang2021 | 2021 | China | Case series | Lvis | 35/35 | 18/17 | 58.0±9.4 | NA | 6/2 | 78.9% ± 4.7 | NA | 97.1% | 8.5 |
| Zhou 2021 | 2021 | China | Cohort study | SES | 190/190 | 141/49 | >57.85 | NA | 137/87 | >84.5 | <14.81 | 100.00% | 8.8 |
| Ari2022 | 2022 | Turkey | Case series | Enterprise | 25/25 | 15/10 | 61.6 ± 8.19 | 15/10 | 6/19 | 86.4±7 | 23.8 ± 8.81 | 100.00% | 18 |
| Gao2022 | 2022 | China | RCT | Wingspan | 176/176 | 128/48 | 56.7 | 89/87 | 80/96 | 78.5 | NA | 89.00% | 36 |
| Park2022 | 2022 | South Korea | Case series | Wingspan | 71/73 | 51/22 | 62.7±11.6 | 49/24 | 46/27 | 79.4 ± 14.3 | NA | 98.63% | 60 |

**Table S3 Perioperative complications and long-term outcomes beyond 30 days**

| **Study** | **Perioperative complications** | | | | | | | | **Long-term outcomes beyond 30 days** | | | | | | **Ischaemic stroke beyond 30 days through 1year** |
| --- | --- | --- | --- | --- | --- | --- | --- | --- | --- | --- | --- | --- | --- | --- | --- |
|  | **TIA** | **Hemorrhagic stroke** | **Ischaemic stroke** | **stroke** | **Death** | **Stroke or death** | **Stroke lacation** | | **TIA** | **Ischaemic stroke** | **Ischaemic stroke or TIA** | **Death** | **Ischaemiac stroke or death** | **ISR** |  |
|  |  |  |  |  |  |  | **AC** | **PC** |  |  |  |  |  |  |  |
| Henkes2005 | NA | 0/15 | 1/15 | 1/15 | 0/15 | 1/15 | 1/6 | 0/9 | NA | NA | NA | NA | NA | NA | NA |
| Bose2007 | NA | 1/45 | 1/45 | 2/45 | 1/45 | 2/45 | 1/23 | 1/22 | 0/43 | 2/43 | 2/43 | 0/43 | 2/43 | 3/40 | NA |
| Fiorella2007 | 1/78 | 3/78 | 2/78 | 5/78 | 4/78 | 5/78 | 2/54 | 3/28 | NA | NA | NA | NA | NA | NA | NA |
| Zaidat2008 | 2/129 | 5/129 | 6/129 | 11/129 | 4/129 | 12/129 | NA | NA | 0/129 | 4/129 | 4/129 | 0/129 | 4/129 | 13/52 | NA |
| Wolfe2009 | 1/51 | 0/51 | 2/51 | 2/51 | 2/51 | 4/51 | NA | NA | 0/51 | 1/51 | 1/51 | 0/51 | 1/51 | 7/29 | NA |
| Costalat2010 | NA | 1/19 | 5/19 | 6/19 | 0/19 | 6/19 | 2/9 | 4/10 | 0/19 | 0/19 | 0/19 | NA | NA | 2/19 | NA |
| Al-Ali2011 | NA | NA | NA | 18/73 | 0/73 | 18/73 | 11/46 | 7/27 | NA | NA | 5/56 | NA | NA | 8/60 | NA |
| Chimowitz2011 | NA | 10/224 | 23/224 | 33/224 | 5/224 | 33/224 | NA | NA | 0/224 | 13/224 | 13/224 | 2/224 | 15/224 | NA | 13/224 |
| Costalat2011 | NA | 4/60 | 8/60 | 12/60 | 1/60 | 13/60 | NA | NA | 1/50 | 0/50 | 1/50 | 0/50 | 1/50 | 11/50 | 0/50 |
| Fiorella2011 | NA | NA | NA | 9/158 | 4/158 | 9/158 | NA | NA | 9/110 | 13/110 | 22/110 | 3/110 | 16/110 | NA | 13/110 |
| Guo2011 | NA | 1/53 | 2/53 | 3/53 | 0/53 | 3/53 | 3/53 | NA | 0/53 | 0/53 | 0/53 | 0/53 | 0/53 | 0/53 | NA |
| Jiang2011 | 7/100 | 2/100 | 3/100 | 5/100 | 0/100 | 5/100 | NA | NA | 6/100 | 4/100 | 10/100 | 1/100 | 5/100 | 12/45 | 2/100 |
| Li2011 | 2/47 | 0/47 | 1/47 | 1/47 | 0/47 | 1/47 | 1/47 | NA | 1/43 | 1/43 | 2/43 | 0/43 | 1/43 | 11/43 | NA |
| Yue2011 | NA | 1/28 | 1/28 | 2/28 | 0/28 | 2/28 | 2/28 | NA | NA | NA | 3/28 | NA | NA | 9/26 | NA |
| Kurre2012 | NA | 7/151 | 13/151 | 20/151 | 6/151 | 20/151 | NA | NA | NA | NA | NA | NA | NA | NA | NA |
| Lee2012 | NA | 2/19 | 0/19 | 2/19 | 0/19 | 2/19 | NA | NA | 0/16 | 0/16 | 0/16 | 0/16 | 0/16 | 1/16 | NA |
| Li2012 | NA | 0/30 | 3/30 | 3/30 | 0/30 | 3/30 | NA | 3/30 | 2/27 | 0/27 | 2/27 | 0/27 | 0/27 | 2/19 | NA |
| Vajda 2012 | NA | 6/189 | 10/189 | 16/189 | 2/189 | 17/189 | NA | NA | NA | NA | 4/174 | NA | NA | 43/174 | NA |
| Gandini2013 | 1/21 | 0/21 | 0/21 | 0/21 | 0/21 | 0/21 | 0/19 | 0/2 | 0/21 | 0/21 | 0/21 | 0/21 | 0/21 | 0/21 | NA |
| Qureshi2013 | NA | 0/8 | 0/8 | 0/8 | 0/8 | 0/8 | 0/5 | 0/3 | NA | 0/7 | NA | 0/7 | 0/7 | 3/7 | NA |
| Rohde2013 | 3/54 | 4/54 | 10/54 | 14/54 | 1/54 | 14/54 | 8/30 | 6/24 | NA | NA | NA | NA | NA | NA | NA |
| Samaniego2013 | 1/45 | 3/45 | 0/45 | 3/45 | 2/45 | 4/45 | NA | NA | 2/43 | 1/43 | 3/43 | NA | NA | 8/21 | NA |
| Shin2013 | 1/77 | 0/77 | 3/77 | 3/77 | 0/77 | 3/77 | 3/55 | 0/24 | 3/76 | 0/76 | 3/76 | 0/76 | 0/76 | 17/69 | NA |
| Zhang2013 | NA | 2/61 | 1/61 | 3/61 | 0/61 | 3/61 | 3/61 | NA | 3/61 | 0/61 | 3/61 | 0/61 | 0/61 | 7/45 | 0/51 |
| Yu2014 | 3/95 | 4/95 | 3/95 | 7/95 | 4/95 | 7/95 | NA | NA | 0/89 | 2/89 | 2/89 | 6/89 | 8/89 | NA | 0/95 |
| Feng 2015 | NA | 1/44 | 3/44 | 4/44 | 0/44 | 4/44 | 2/25 | 2/19 | 0/44 | 2/44 | 2/44 | 0/44 | 2/44 | 3/44 | NA |
| Li2015 | NA | 8/433 | 21/433 | 29/433 | 3/433 | 29/433 | 15/254 | 14/179 | 17/365 | 20/365 | 37/365 | 8/365 | 28/365 | 62/208 | 12/433 |
| Miao2015 | NA | 1/38 | 2/38 | 3/38 | 0/38 | 3/38 | NA | NA | NA | NA | NA | NA | NA | NA | NA |
| Yin2015 | NA | 2/48 | 2/48 | 4/48 | 0/48 | 4/48 | NA | NA | NA | 6/48 | NA | 1/48 | 7/48 | NA | NA |
| Duan2016 | NA | 0/44 | 4/44 | 4/44 | 0/44 | 4/44 | NA | NA | 0/44 | 2/44 | 2/44 | 0/44 | 2/44 | 5/44 | NA |
| Gao2016 | NA | 0/100 | 2/100 | 2/100 | 0/100 | 2/100 | 0/55 | 2/45 | NA | NA | NA | NA | NA | NA | NA |
| Lee 2016 | NA | 1/24 | 2/24 | 3/24 | 1/24 | 3/24 | 2/20 | 1/10 | 0/24 | 0/24 | 0/24 | NA | NA | 1/20 | NA |
| Wang 2016 | NA | 0/60 | 3/60 | 3/60 | 0/60 | 3/60 | NA | NA | 3/45 | 2/45 | 5/45 | NA | NA | 6/45 | NA |
| Yeo2016 | 1/50 | 0/50 | 6/50 | 6/50 | 0/50 | 6/50 | NA | NA | NA | NA | 3/50 | 0/50 | NA | 1/50 | NA |
| Zhao2016 | NA | 8/278 | 4/278 | 12/278 | 2/278 | 12/278 | 12/278 | NA | NA | 8/278 | NA | NA | NA | NA | 4/278 |
| Dong 2018 | NA | 0/20 | 1/20 | 1/20 | 0/20 | 1/20 | NA | NA | 0/20 | 0/20 | 0/20 | NA | NA | 3/20 | NA |
| Du 2018 | NA | 0/45 | 1/45 | 1/45 | 0/45 | 1/45 | 1/25 | 0/21 | 0/45 | 0/45 | 0/45 | 0/45 | 0/45 | 0/33 | NA |
| Gruber2018 | 2/11 | 0/11 | 0/11 | 0/11 | 0/11 | 0/11 | 0/6 | 0/5 | 4/11 | 1/11 | 5/11 | NA | NA | 6/11 | NA |
| Huang 2019 | NA | 2/68 | 1/68 | 3/68 | 0/68 | 3/68 | 1/54 | 2/16 | NA | NA | NA | NA | NA | NA | NA |
| Li2019 | NA | 2/55 | 0/55 | 2/55 | 0/55 | 2/55 | 2/55 | NA | 1/49 | 2/49 | 3/49 | 0/49 | 2/49 | 3/49 | 2/49 |
| Salik 2019 | 0/68 | 1/68 | 0/68 | 1/68 | 0/68 | 1/68 | 1/29 | 0/39 | 0/68 | 0/68 | 0/68 | NA | NA | 2/60 | NA |
| Xu 2019 | 3/71 | 0/71 | 0/71 | 0/71 | 0/71 | 0/71 | 0/54 | 0/17 | NA | NA | NA | NA | NA | NA | NA |
| Zhang2019 | NA | 1/69 | 1/69 | 2/69 | 0/69 | 2/69 | NA | 1/69 | 2/63 | 3/63 | 5/63 | 2/63 | 5/63 | 7/63 | 3/63 |
| Alexander2020 | NA | 2/152 | 2/152 | 4/152 | 2/152 | 4/152 | NA | NA | 0/129 | 7/129 | 7/129 | 0/129 | 7/129 | 18/102 | 7/129 |
| Cao2020 | 2/32 | 0/32 | 1/32 | 1/32 | 0/32 | 1/32 | 0/17 | 1/15 | 0/32 | 1/32 | 1/32 | 0/32 | 1/32 | 0/26 | NA |
| Li 2020 | 2/67 | 0/67 | 1/67 | 1/67 | 0/67 | 1/67 | NA | NA | 1/67 | 0/67 | 1/67 | 0/67 | 0/67 | 4/55 | NA |
| Meyer2020 | 1/76 | 0/76 | 5/76 | 5/76 | 2/76 | 5/76 | NA | NA | 4/60 | 1/60 | 5/60 | 1/60 | 1/60 | 15/60 | NA |
| Park2020 | NA | 6/95 | 11/95 | 17/95 | 0/95 | 17/95 | NA | NA | 7/95 | 5/95 | 12/95 | 5/95 | 10/95 | 11/80 | 2/95 |
| Tian2020 | NA | 0/51 | 1/51 | 1/51 | 0/51 | 1/51 | NA | 1/51 | 0/51 | 4/51 | 4/51 | NA | NA | 5/38 | NA |
| Cui 2021 | NA | 3/130 | 2/130 | 5/130 | 1/130 | 5/130 | 3/54 | 2/76 | 0/125 | 5/125 | 5/125 | 1/125 | 6/125 | 17/118 | NA |
| Shen2021 | NA | NA | NA | NA | 0/41 | 4/41 | 4/41 | NA | NA | 3/41 | NA | 0/41 | NA | 9/41 | 3/41 |
| Sun 2021 | NA | 3/104 | 4/104 | 7/104 | 1/104 | 7/104 | 3/47 | 4/58 | NA | NA | NA | NA | NA | NA | NA |
| Tang2021 | NA | 0/31 | 1/31 | 1/31 | 0/31 | 1/31 | 1/22 | 0/9 | 0/31 | 0/31 | 0/31 | 0/31 | 0/31 | 2/21 | NA |
| Wang2021 | NA | 1/35 | 0/35 | 1/35 | 0/35 | 1/35 | 1/35 | NA | 0/35 | 0/35 | 0/35 | 0/35 | 0/35 | 1/35 | NA |
| Zhou 2021 | 2/190 | 7/190 | 2/190 | 9/190 | 1/190 | 9/190 | 9/190 | NA | 3/190 | 3/190 | 6/190 | 1/190 | 4/190 | 30/152 | NA |
| Ari2022 | NA | 0/25 | 2/25 | 2/25 | 0/25 | 2/25 | 1/6 | 1/19 | 2/25 | 1/25 | 3/25 | 0/25 | 1/25 | 2/25 | NA |
| Gao2022 | NA | 4/176 | 5/176 | 9/176 | 2/176 | 9/176 | NA | NA | NA | 10/168 | NA | 5/160 | 15/160 | NA | 5/176 |
| Park2022 | NA | 4/71 | 2/71 | 6/71 | 2/71 | 6/71 | NA | NA | NA | 6/71 | NA | 1/71 | 6/71 | 10/73 | NA |

**Table S4 Multivariate meta-regression analyses**

| **Table S4a Multivariate meta-regression analyses of perioperative TIA** | | | | | |
| --- | --- | --- | --- | --- | --- |
| **Covariates** | **Number of studies** | **Size of outcomes** | **exp(b)** | **(95% CI)** | **P value** |
| Publication time | 15 | 942 | 1.0147 | 0.7338-1.4031 | 0.920 |
| Study location | 15 | 942 | 0.9953 | 0.7329-1.3516 | 0.972 |
| Study design | 15 | 942 | 0.8611 | 0.3988-1.8594 | 0.666 |
| Stent variety | 15 | 942 | 0.9648 | 0.6287-1.4805 | 0.852 |
| Mean age | 15 | 942 | 0.9535 | 0.6981-1.3022 | 0.734 |
| Preprocedual stenosis rate | 15 | 942 | 1.0024 | 0.7842-1.2813 | 0.983 |

| **Table S4b Multivariate meta-regression analyses of perioperative Haemorrhagic stroke** | | | | | |
| --- | --- | --- | --- | --- | --- |
| **Covariates** | **Number of studies** | **Size of outcomes** | **exp(b)** | **(95% CI)** | **p value** |
| Publication time | 51 | 3957 | 0.9919 | 0.9100-1.0811 | 0.849 |
| Study location | 51 | 3957 | 1.0048 | 0.8977-1.1246 | 0.932 |
| Study design | 51 | 3957 | 0.9909 | 0.8855-1.1089 | 0.871 |
| Stent variety | 51 | 3957 | 0.9936 | 0.9085-1.0867 | 0.886 |
| Mean age | 51 | 3957 | 1.0117 | 0.9300-1.1006 | 0.783 |
| Preprocedual stenosis rate | 51 | 3957 | 0.9990 | 0.9199-1.0848 | 0.980 |

| **Table S4c Multivariate meta-regression analyses of perioperative Ischaemiac stroke** | | | | | |
| --- | --- | --- | --- | --- | --- |
| **Covariates** | **Number of studies** | **Size of outcomes** | **exp(b)** | **(95% CI)** | **p value** |
| Publication time | 50 | 3821 | 0.9763 | 0.8844-1.0777 | 0.627 |
| Study location | 50 | 3821 | 1.0103 | 0.8986-1.1358 | 0.861 |
| Study design | 50 | 3821 | 0.9707 | 0.8919-1.0564 | 0.482 |
| Stent variety | 50 | 3821 | 1.0069 | 0.9276-1.0929 | 0.867 |
| Mean age | 50 | 3821 | 1.0198 | 0.9344-1.1129 | 0.654 |
| Preprocedual stenosis rate | 50 | 3821 | 1.0078 | 0.9342-1.0871 | 0.838 |

| **Table S4d Multivariate meta-regression analyses of perioperative stroke** | | | | | |
| --- | --- | --- | --- | --- | --- |
| **Covariates** | **Number of studies** | **Size of outcomes** | **exp(b)** | **(95% CI)** | **p value** |
| Publication time | 52 | 4052 | 0.9668 | 0.8760-1.0670 | 0.494 |
| Study location | 52 | 4052 | 1.0115 | 0.9030-1.1331 | 0.840 |
| Study design | 52 | 4052 | 0.9482 | 0.8744-1.0282 | 0.192 |
| Stent variety | 52 | 4052 | 1.0047 | 0.9265-1.0895 | 0.908 |
| Mean age | 52 | 4052 | 1.0335 | 0.9474-1.1274 | 0.449 |
| Preprocedual stenosis rate | 52 | 4052 | 1.0061 | 0.9350-1.0826 | 0.868 |

| **Table S4e Multivariate meta-regression analyses of perioperative death** | | | | | |
| --- | --- | --- | --- | --- | --- |
| **Covariates** | **Number of studies** | **Size of outcomes** | **exp(b)** | **(95% CI)** | **p value** |
| Publication time | 54 | 4133 | 1.0044 | 0.9144-1.1032 | 0.926 |
| Study location | 54 | 4133 | 0.9879 | 0.8894-1.0974 | 0.817 |
| Study design | 54 | 4133 | 0.9969 | 0.8937-1.1119 | 0.954 |
| Stent variety | 54 | 4133 | 0.9929 | 0.9189-1.0728 | 0.854 |
| Mean age | 54 | 4133 | 1.0054 | 0.9253-1.0925 | 0.896 |
| Preprocedual stenosis rate | 54 | 4133 | 0.9941 | 0.9264-1.0667 | 0.866 |

| **Table S4f Multivariate meta-regression analyses of perioperative stroke or death** | | | | | |
| --- | --- | --- | --- | --- | --- |
| **Covariates** | **Number of studies** | **Size of outcomes** | **exp(b)** | **(95% CI)** | **p value** |
| Publication time | 58 | 4632 | 0.9625 | 0.8793-1.0537 | 0.401 |
| Study location | 58 | 4632 | 1.0057 | 0.9043-1.1184 | 0.916 |
| Study design | 58 | 4632 | 0.952 | 0.8869-1.0220 | 0.170 |
| Stent variety | 58 | 4632 | 1.0069 | 0.9306-1.0894 | 0.862 |
| Mean age | 58 | 4632 | 1.0356 | 0.9534-1.1249 | 0.400 |
| Preprocedual stenosis rate | 58 | 4632 | 1.0053 | 0.9412-1.0738 | 0.873 |

| **Table S4g Multivariate meta-regression analyses of TIA beyond 30 days** | | | | | |
| --- | --- | --- | --- | --- | --- |
| **Covariates** | **Number of studies** | **Size of outcomes** | **exp(b)** | **(95% CI)** | **p value** |
| Publication time | 33 | 2528 | 1.0547 | 0.9392-1.1844 | 0.354 |
| Study location | 33 | 2528 | 0.9663 | 0.8208-1.1376 | 0.669 |
| Study design | 33 | 2528 | 0.9791 | 0.8867-1.0812 | 0.665 |
| Stent variety | 33 | 2528 | 0.9603 | 0.8477-1.0880 | 0.511 |
| Mean age | 33 | 2528 | 0.9939 | 0.8806-1.1218 | 0.918 |
| Preprocedual stenosis rate | 33 | 2528 | 0.9779 | 0.8808-1.0857 | 0.664 |

| **Table S4h Multivariate meta-regression analyses of Ischaemiac stroke beyond 30 days** | | | | | |
| --- | --- | --- | --- | --- | --- |
| **Covariates** | **Number of studies** | **Size of outcomes** | **exp(b)** | **(95% CI)** | **p value** |
| Publication time | 41 | 2809 | 1.0316 | 0.9249-1.1507 | 0.566 |
| Study location | 41 | 2809 | 0.9742 | 0.8464-1.1212 | 0.707 |
| Study design | 41 | 2809 | 0.9869 | 0.8983-1.0843 | 0.778 |
| Stent variety | 41 | 2809 | 0.9701 | 0.8686-1.0835 | 0.580 |
| Mean age | 41 | 2809 | 0.9922 | 0.8891-1.1072 | 0.885 |
| Preprocedual stenosis rate | 41 | 2809 | 0.9912 | 0.9040-1.0867 | 0.846 |

| **Table S4i Multivariate meta-regression analyses of Ischaemiac stroke or TIA beyond 30 days** | | | | | |  |
| --- | --- | --- | --- | --- | --- | --- |
| **Covariates** | **Number of studies** | **Size of outcomes** | **exp(b)** | **(95% CI)** | **p value** | |
| Publication time | 43 | 3114 | 1.0480 | 0.9383-1.1705 | 0.395 | |
| Study location | 43 | 3114 | 0.9489 | 0.8389-1.0734 | 0.394 |  |
| Study design | 43 | 3114 | 0.9782 | 0.8880-1.0776 | 0.647 |  |
| Stent variety | 43 | 3114 | 0.9523 | 0.8680-1.0449 | 0.293 |  |
| Mean age | 43 | 3114 | 0.9542 | 0.8639-1.0539 | 0.345 |  |
| Preprocedual stenosis rate | 43 | 3114 | 0.9599 | 0.8799-1.0472 | 0.347 |  |

| **Table S4j Multivariate meta-regression analyses of death beyond 30 days** | | | | | |
| --- | --- | --- | --- | --- | --- |
| **Covariates** | **Number of studies** | **Size of outcomes** | **exp(b)** | **(95% CI)** | **p value** |
| Publication time | 34 | 2578 | 1.0182 | 0.9078-1.1420 | 0.750 |
| Study location | 34 | 2578 | 1.0047 | 0.8545-1.1813 | 0.953 |
| Study design | 34 | 2578 | 0.9914 | 0.8985-1.0938 | 0.858 |
| Stent variety | 34 | 2578 | 0.9826 | 0.8684-1.1117 | 0.772 |
| Mean age | 34 | 2578 | 1.0043 | 0.8922-1.1303 | 0.942 |
| Preprocedual stenosis rate | 34 | 2578 | 0.9913 | 0.8932-1.1001 | 0.864 |

| **Table S4k Multivariate meta-regression analyses of Ischaemiac stroke or death beyond 30 days** | | | | | |
| --- | --- | --- | --- | --- | --- |
| **Covariates** | **Number of studies** | **Size of outcomes** | **exp(b)** | **(95% CI)** | **p value** |
| Publication time | 33 | 2528 | 1.0547 | 0.9392-1.1844 | 0.354 |
| Study location | 33 | 2528 | 0.9663 | 0.8208-1.1376 | 0.669 |
| Study design | 33 | 2528 | 0.9791 | 0.8867-1.0812 | 0.665 |
| Stent variety | 33 | 2528 | 0.9603 | 0.8477-1.0880 | 0.511 |
| Mean age | 33 | 2528 | 0.9939 | 0.8806-1.1218 | 0.918 |
| Preprocedual stenosis rate | 33 | 2528 | 0.9779 | 0.8808-1.0857 | 0.664 |

| **Table S4l Multivariate meta-regression analyses of ISR** | | | | | |
| --- | --- | --- | --- | --- | --- |
| **Covariates** | **Number of studies** | **Size of outcomes** | **exp(b)** | **(95% CI)** | **p value** |
| Publication time | 40 | 2009 | 0.9691 | 0.8492-1.1059 | 0.632 |
| Study location | 40 | 2009 | 0.9438 | 0.8107-1.0988 | 0.444 |
| Study design | 40 | 2009 | 1.0288 | 0.9037-1.1712 | 0.659 |
| Stent variety | 40 | 2009 | 0.9378 | 0.8426-1.0438 | 0.231 |
| Mean age | 40 | 2009 | 0.9513 | 0.8342-1.0847 | 0.444 |
| Preprocedual stenosis rate | 40 | 2009 | 1.0019 | 0.8914-1.1261 | 0.974 |

| **Table S4m Multivariate meta-regression analyses of death beyond 30 days through 1 year** | | | | | |
| --- | --- | --- | --- | --- | --- |
| **Covariates** | **Number of studies** | **Size of outcomes** | **exp(b)** | **(95% CI)** | **p value** |
| Publication time | 13 | 1853 | 1.0103 | 0.8490-1.2024 | 0.893 |
| Study location | 34 | 1853 | 0.9408 | 0.7617-1.1622 | 0.517 |
| Study design | 34 | 1853 | 0.9983 | 0.8844-1.1270 | 0.975 |
| Mean age | 34 | 1853 | 0.9835 | 0.8548-1.1315 | 0.787 |
| Preprocedual stenosis rate | 34 | 1853 | 0.9886 | 0.8670-1.1274 | 0.843 |

**Figure S1 Assessment of study quality**

**Figure S1a Risk of bias summary of RCTs**

**
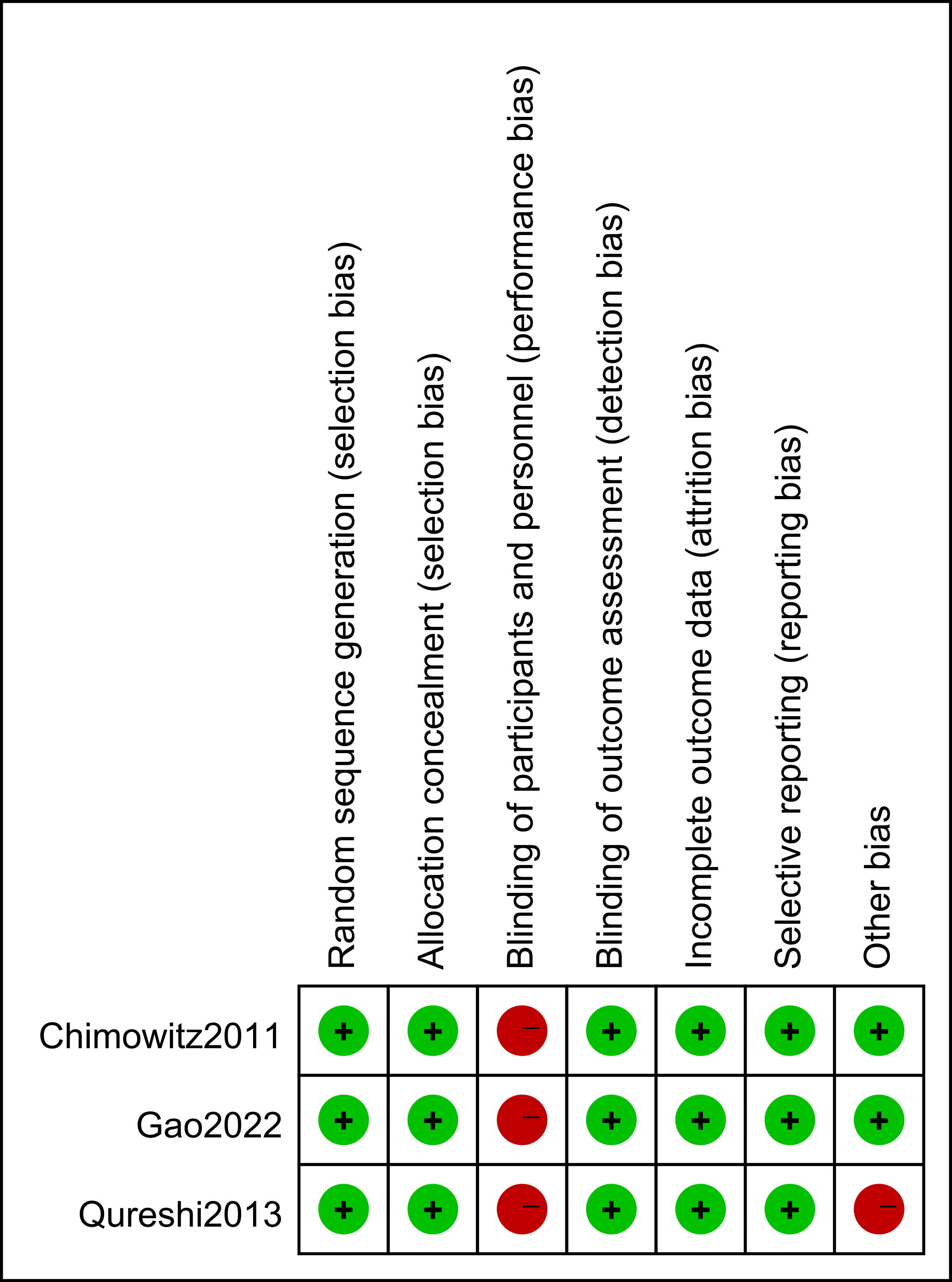
**

**Figure S1b Risk of bias graph of RCTs**

**
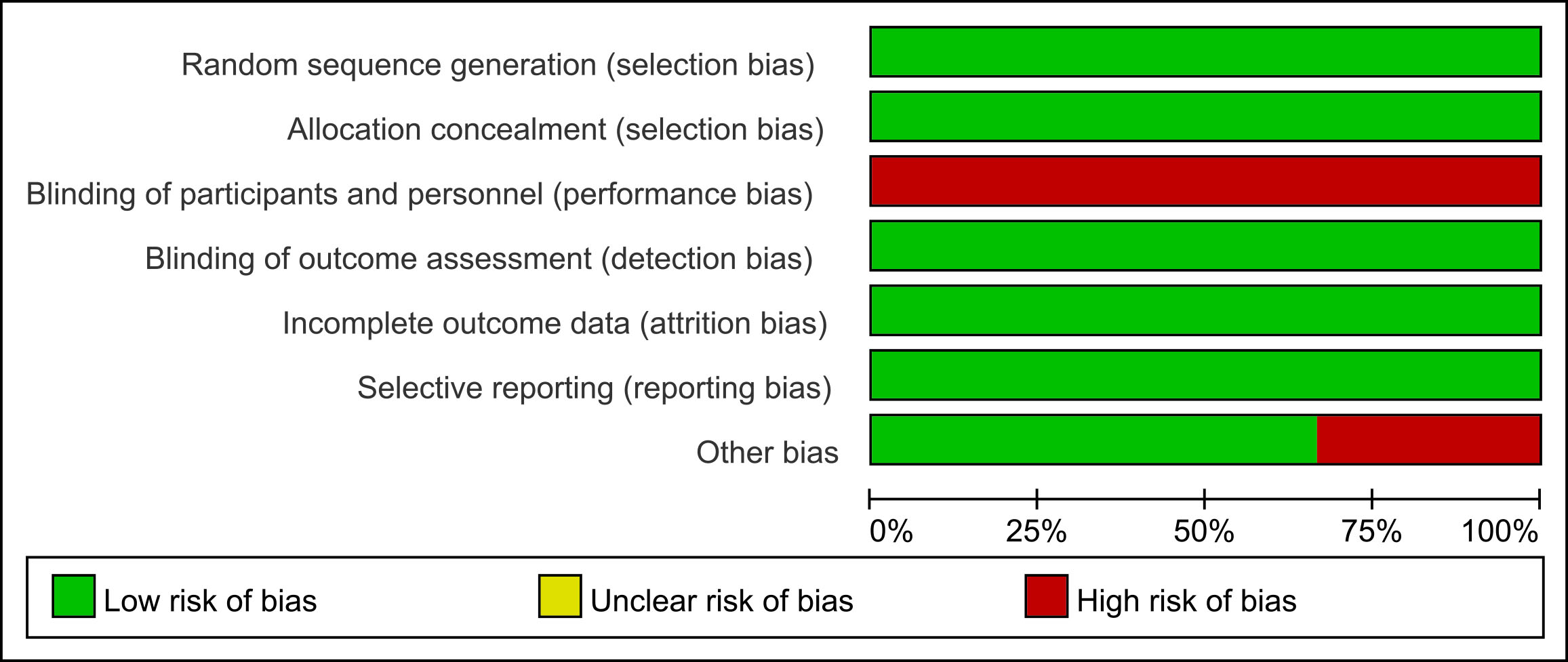
**

**Figure S1c NOS scale of cohort studies**

**
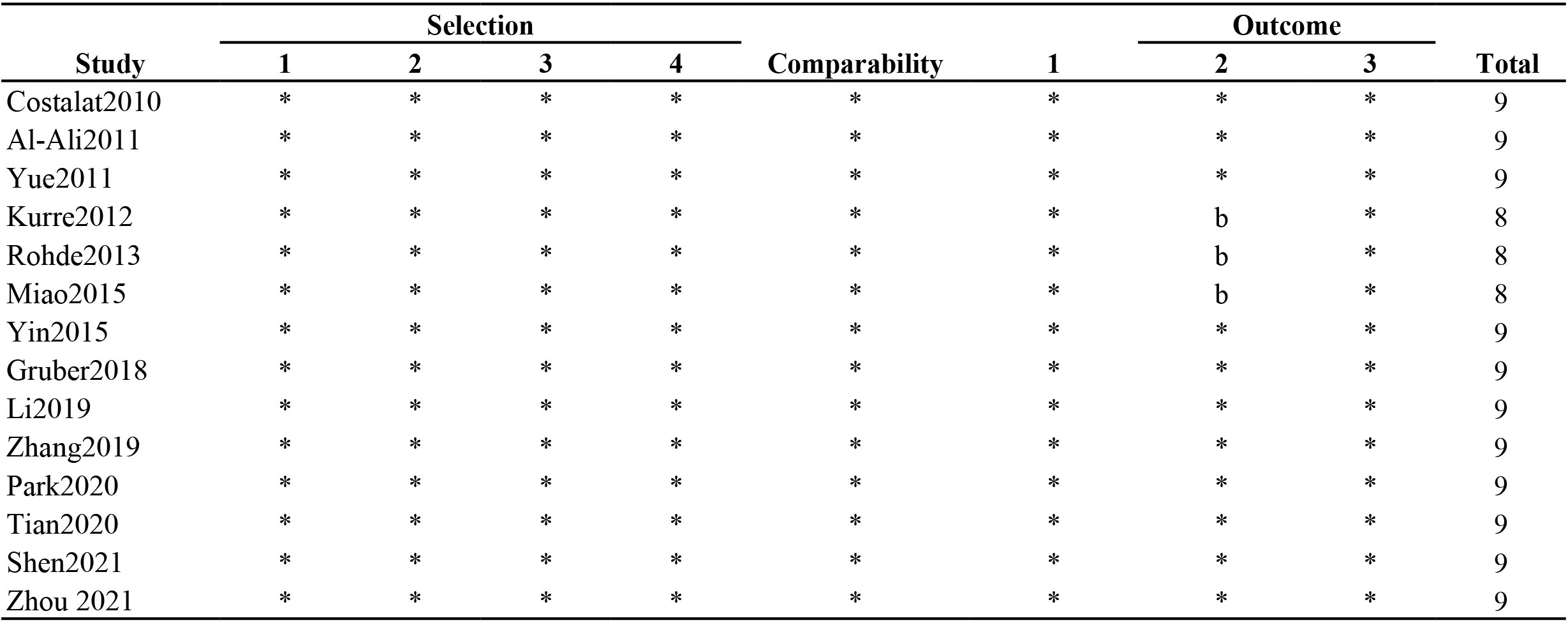
**

**Figure S2 The forest plots of each group**

**Figure S2a Perioperative TIA Figure S2b Perioperative Haemorrhagic stroke**

**
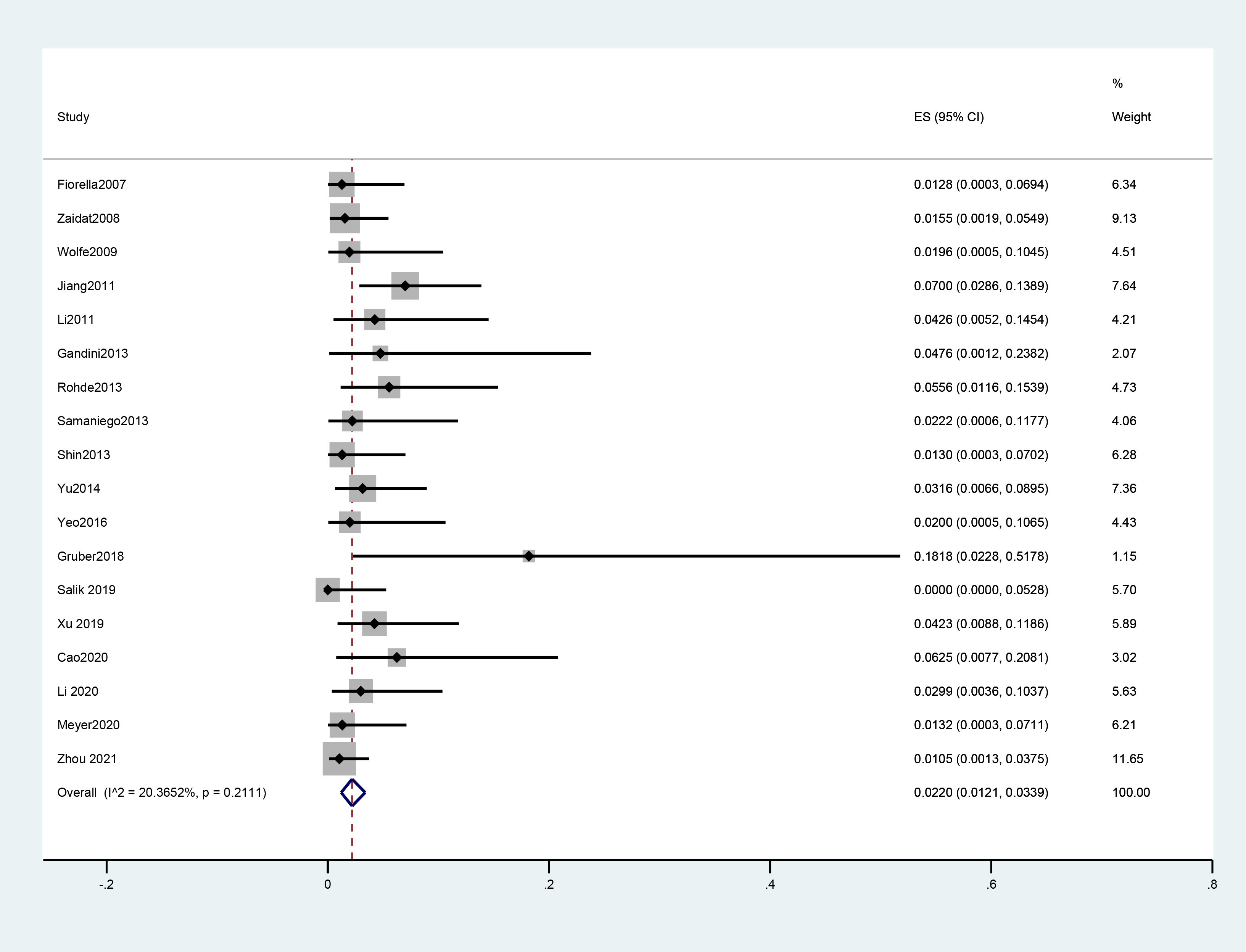

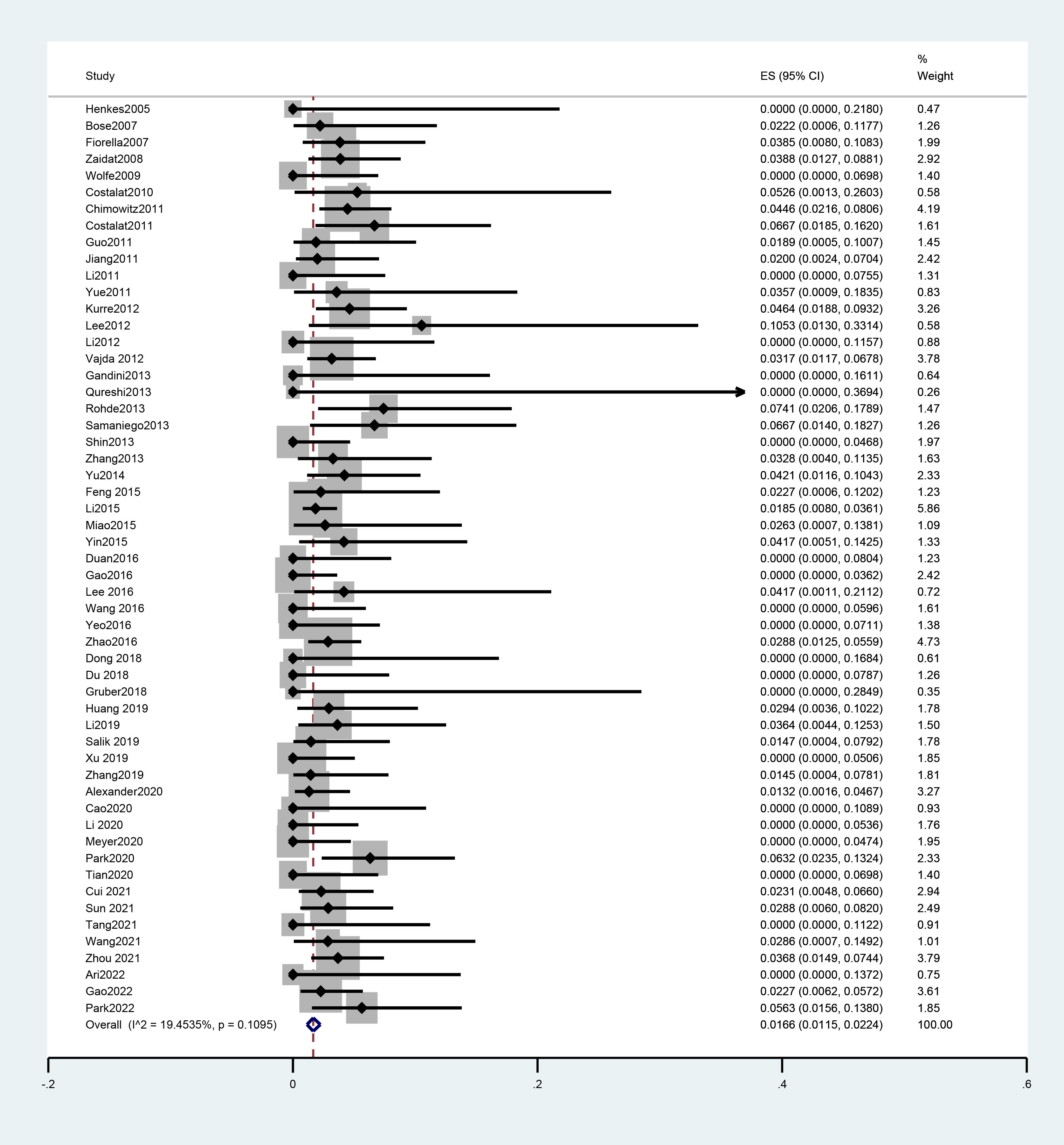
**

**Figure S2c Perioperative Ischaemiac stroke Figure S2d Perioperative stroke**

**
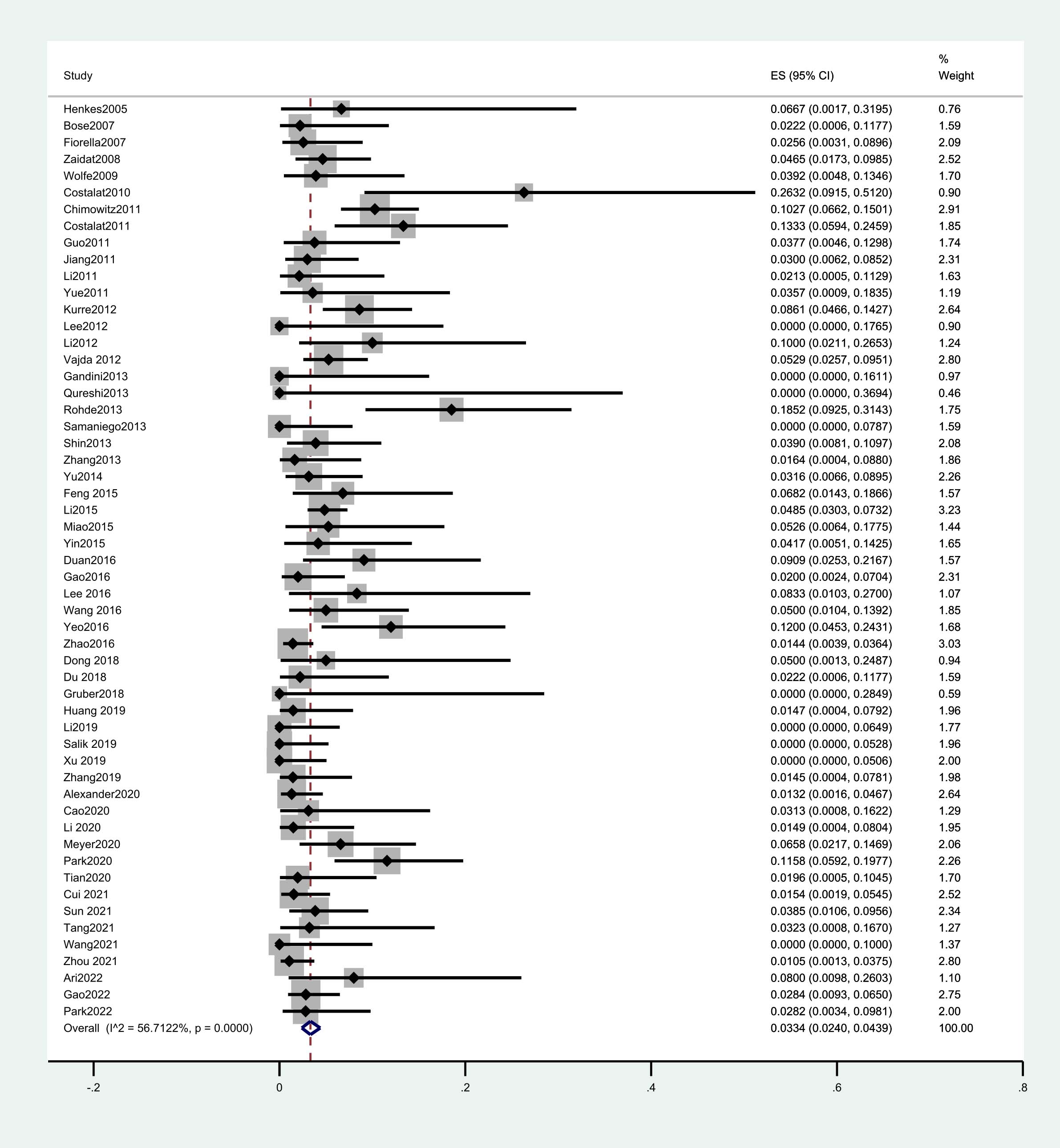

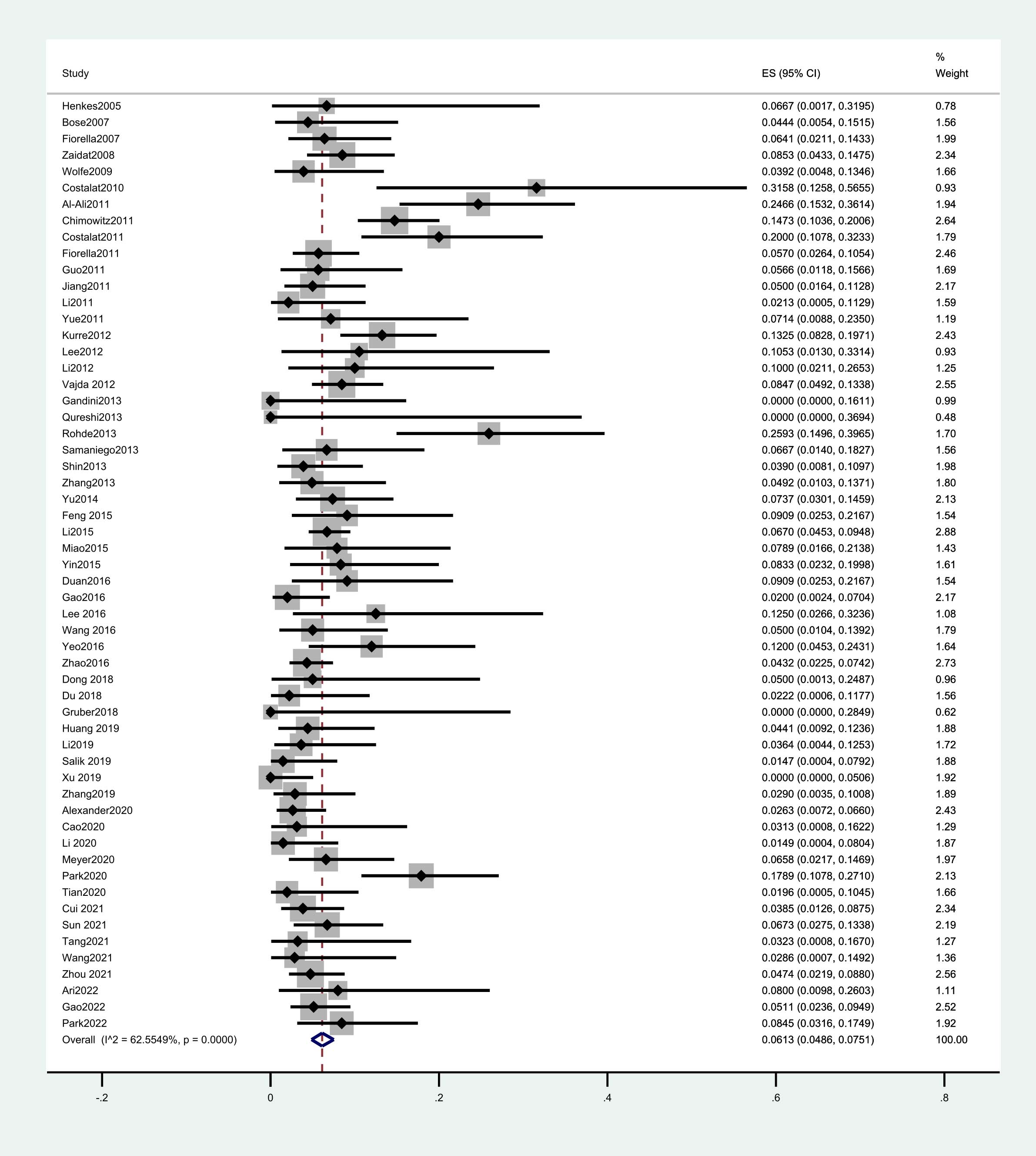
**

**Figure S2e Perioperative death Figure S2f Perioperative stroke or death**

**
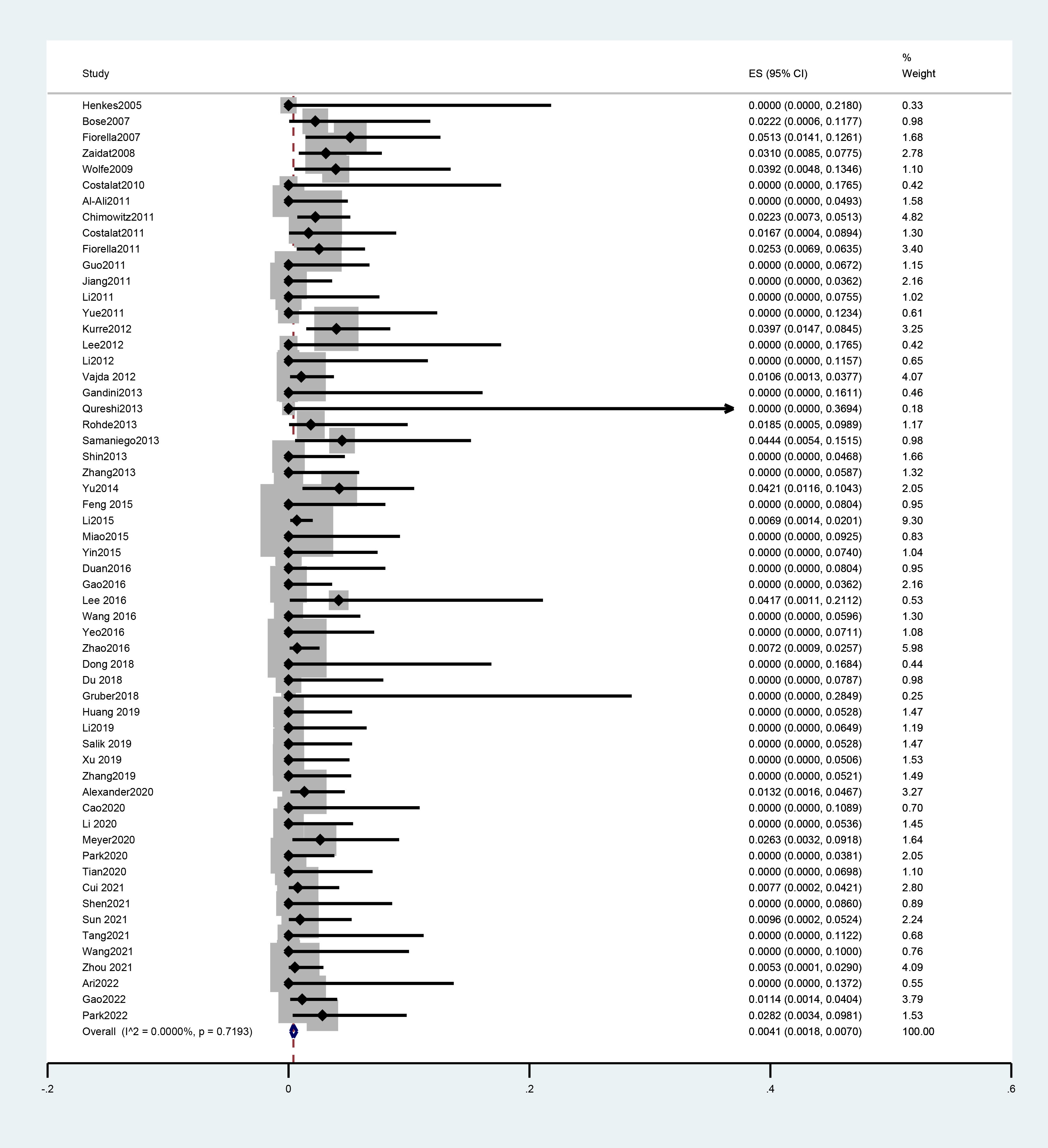

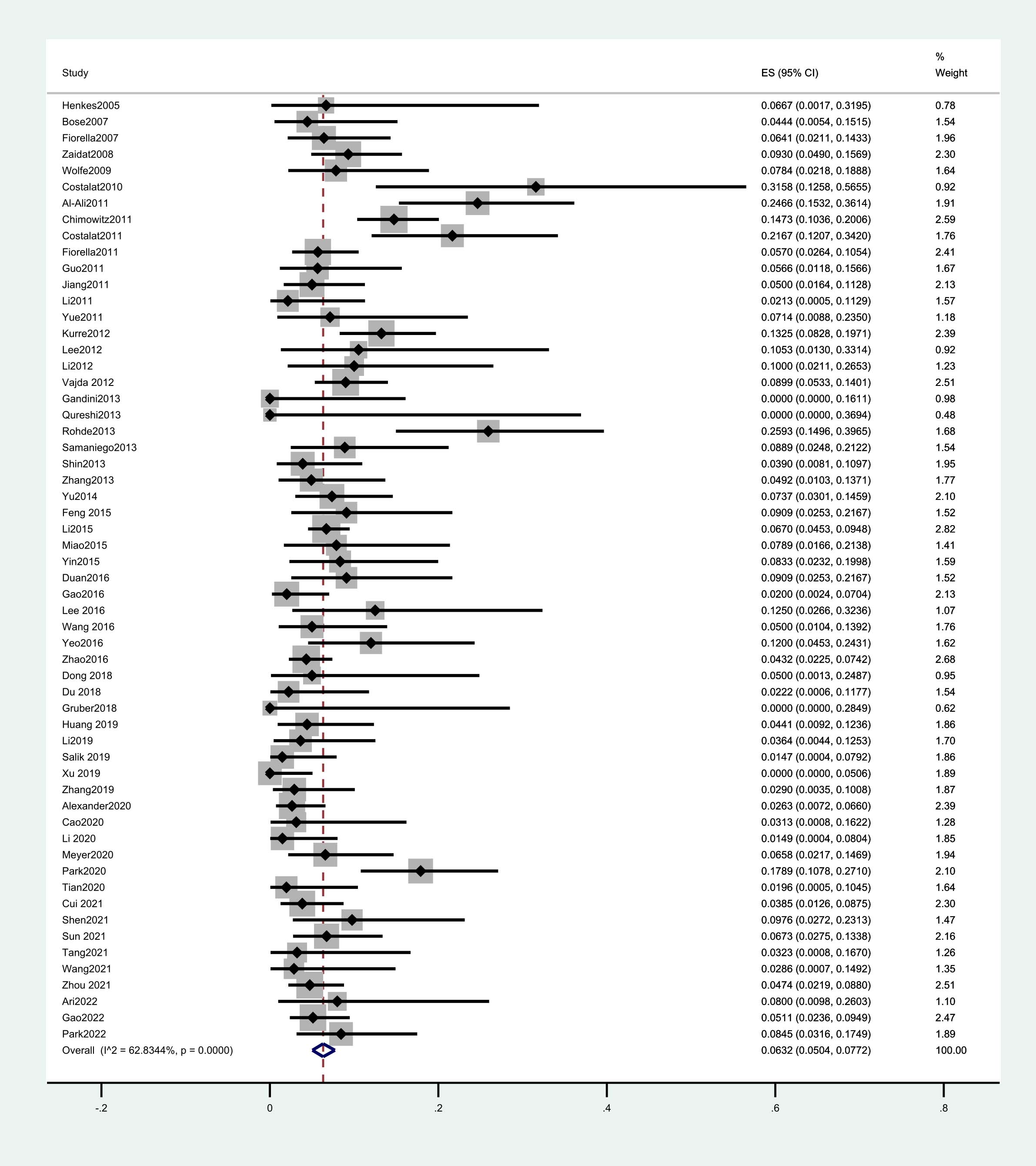
**

**Figure S2g TIA beyond 30 days Figure S2h Ischaemiac stroke beyond 30 days**

**
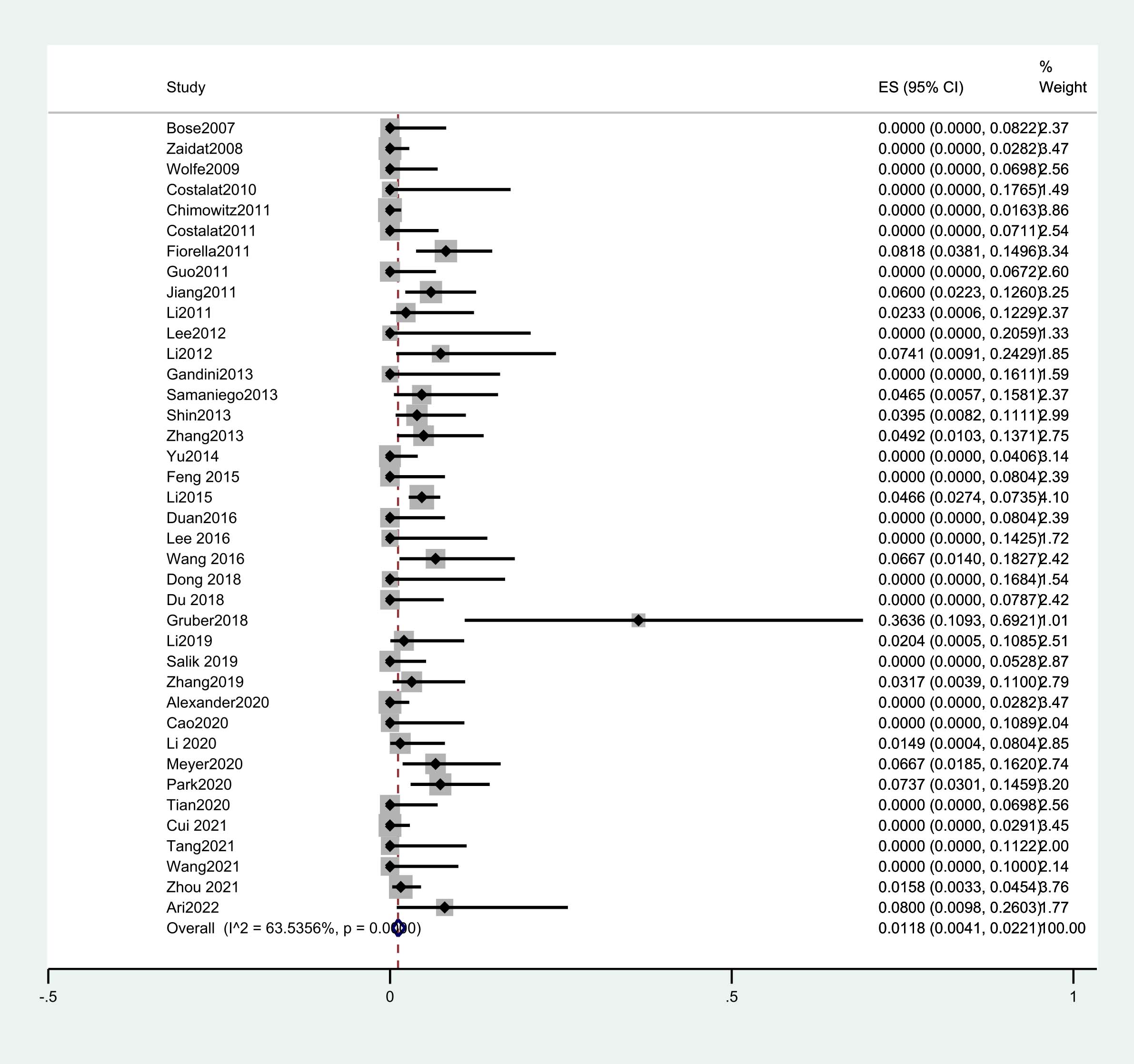

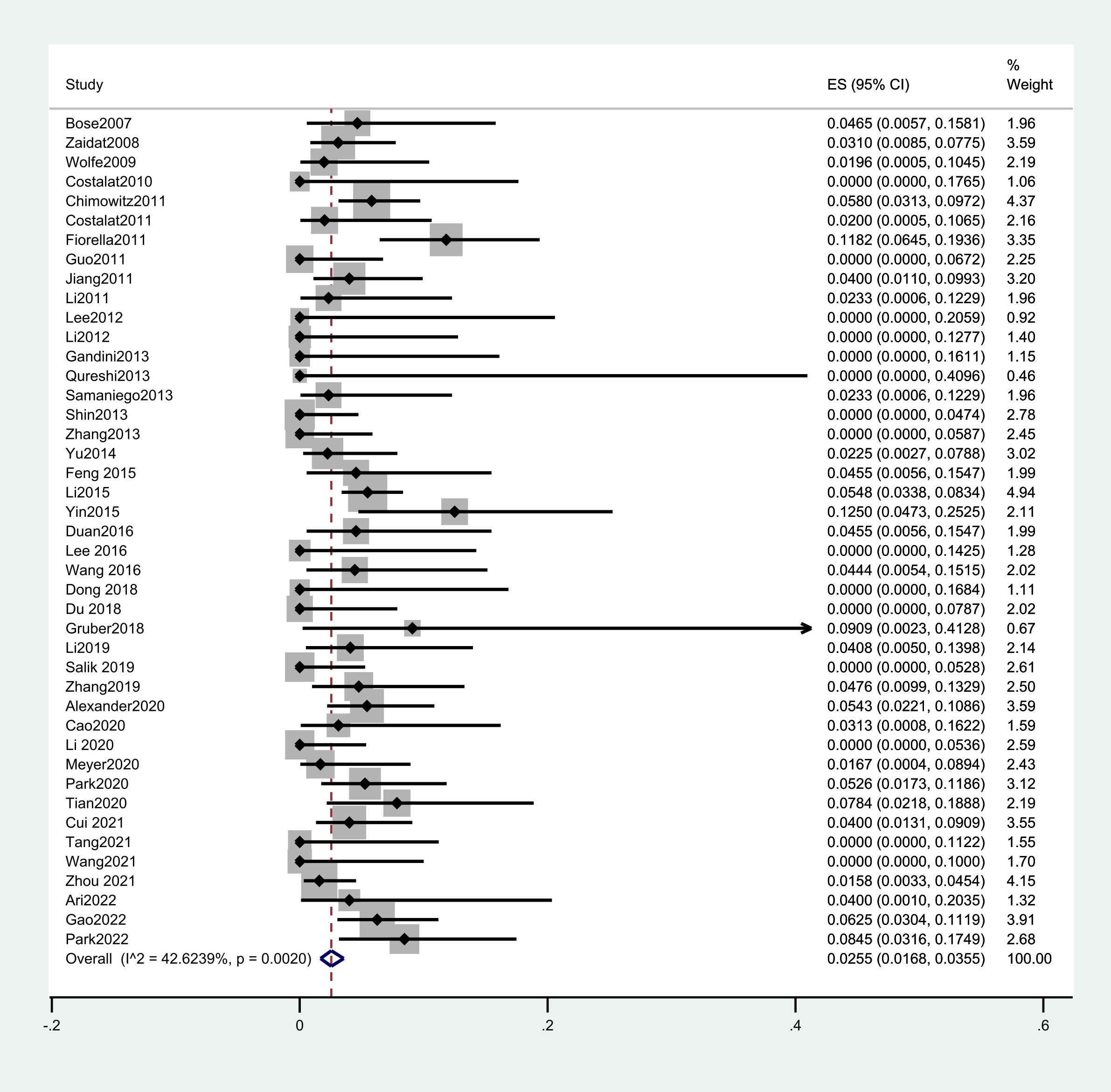
**

**Figure S2i Ischaemiac stroke or TIA beyond 30 days Figure S2j Death beyond 30 days**

**
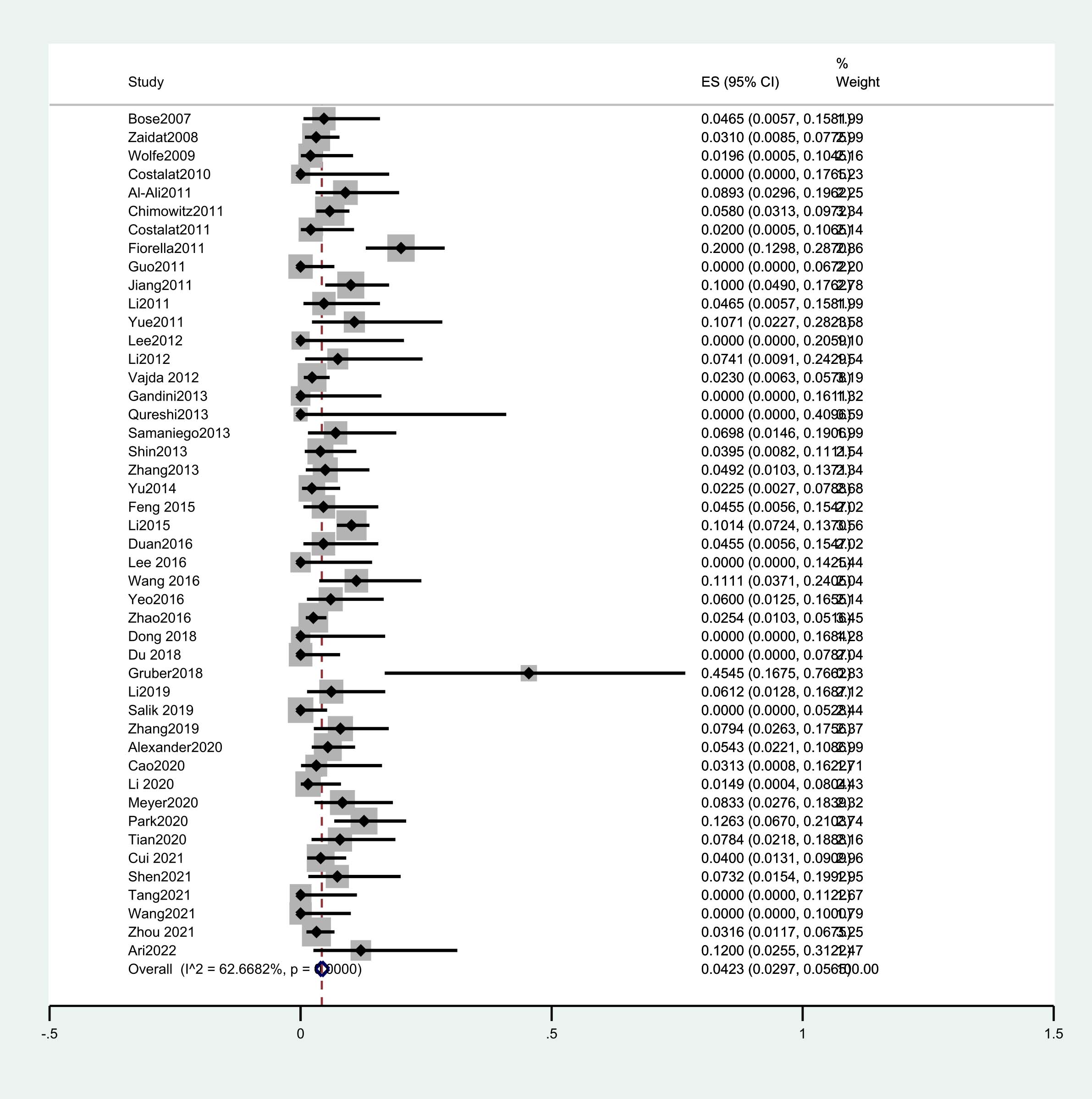

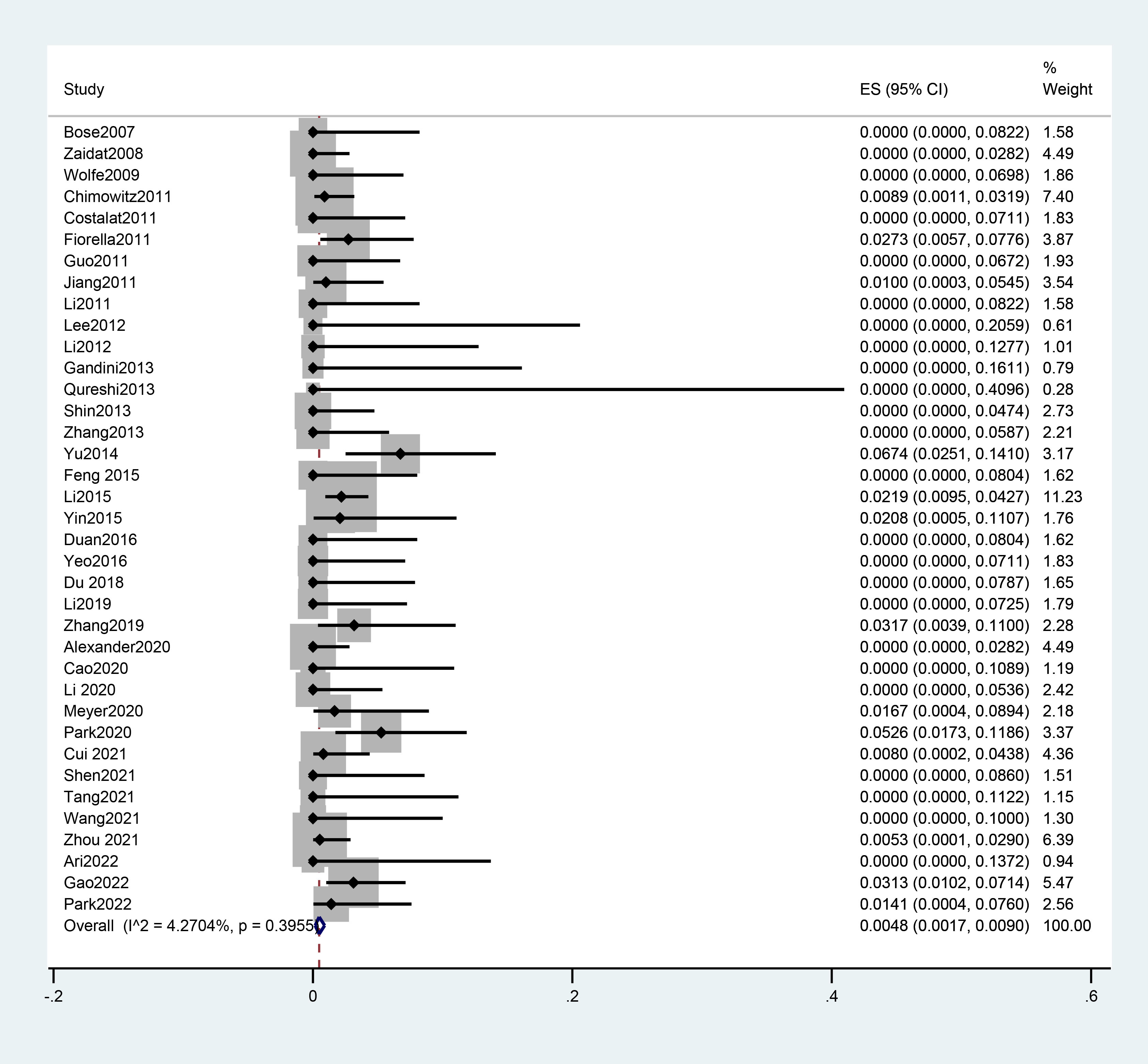
**

**Figure S2k Ischaemiac stroke or death beyond 30 days Figure S2l ISR**

**
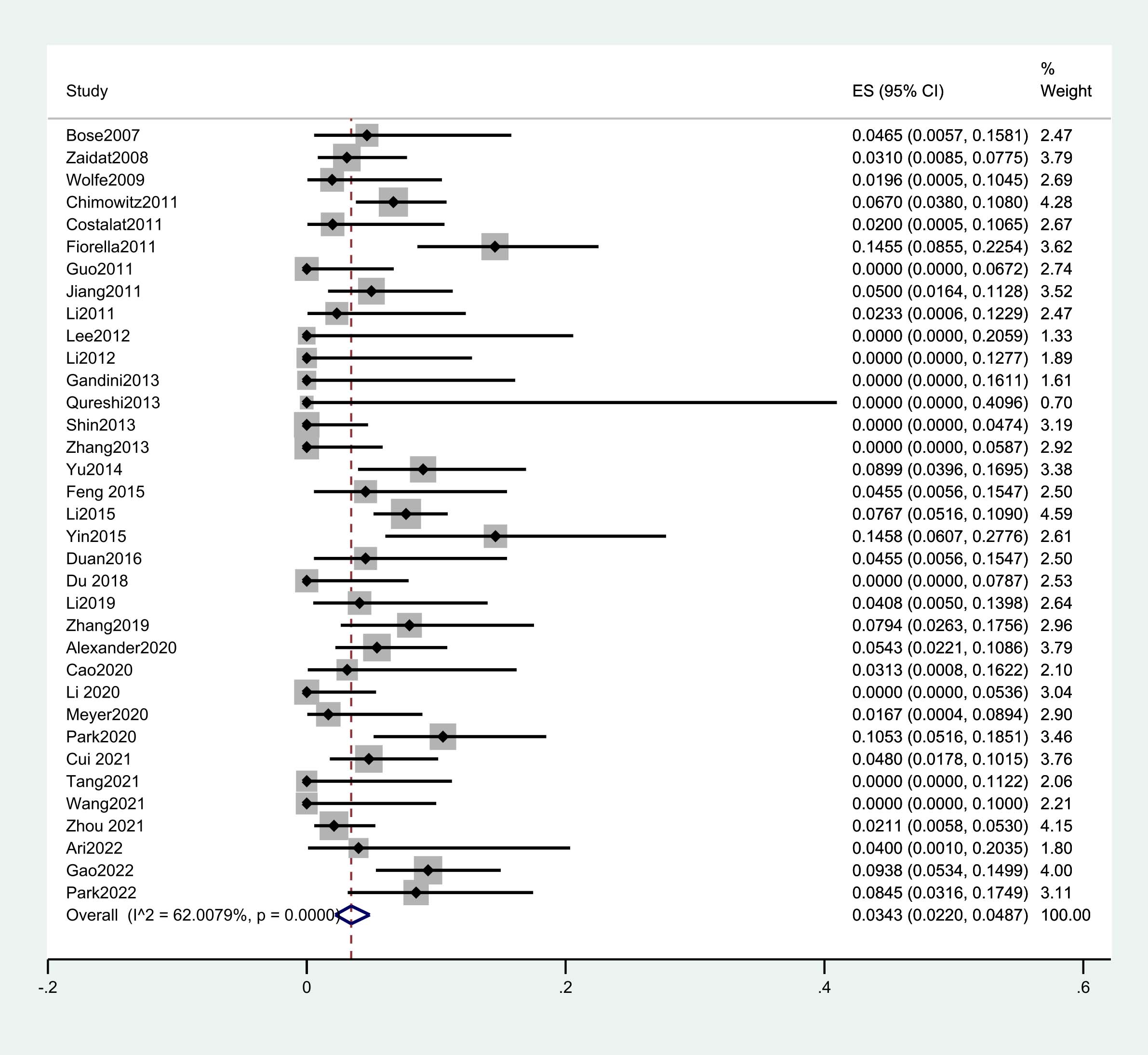

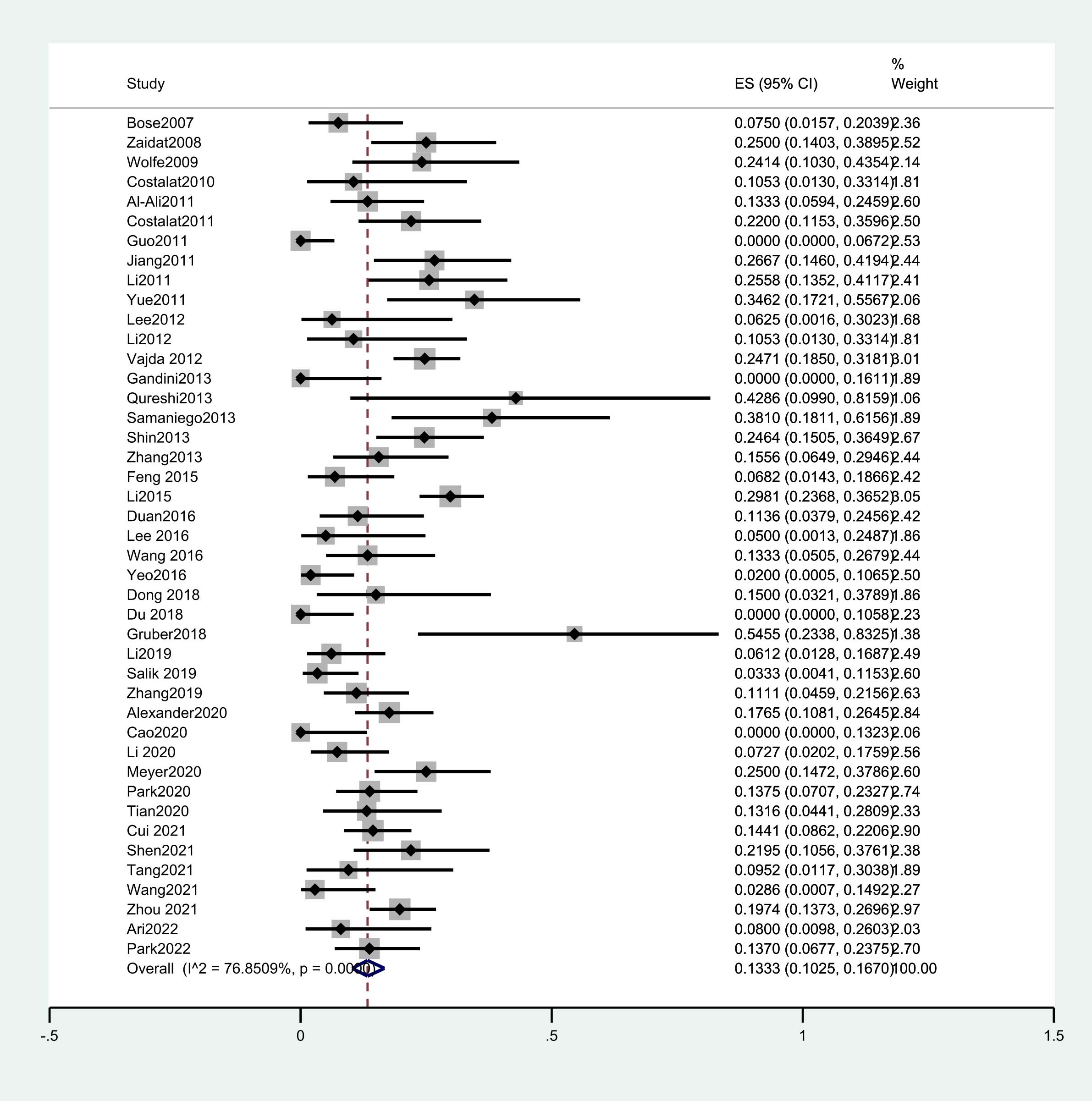
**

**Figure S2m ischemic stroke beyond 30 days through 1 year**

**
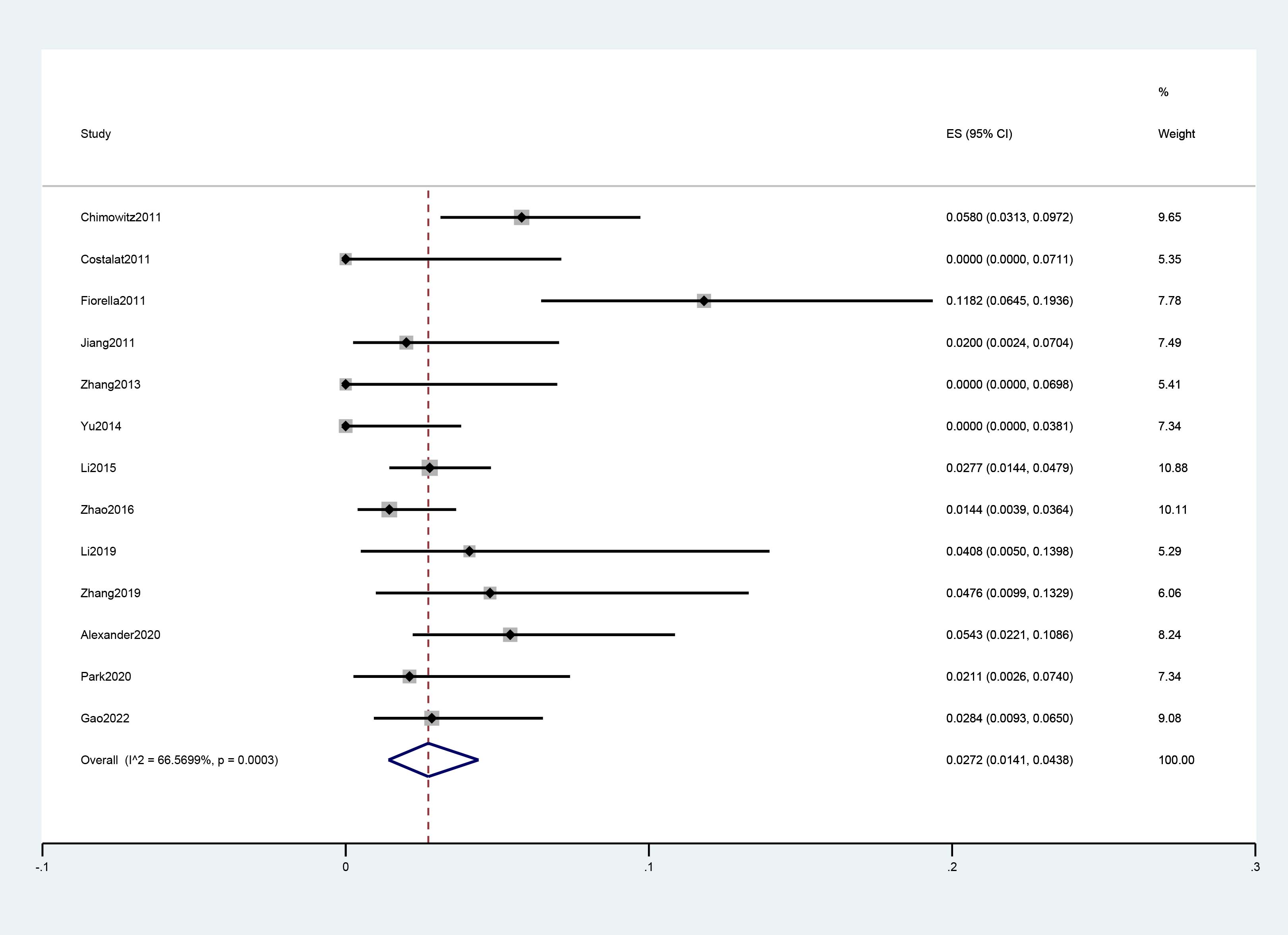
**

**Figure S3 Funnel plots of each group**

**Figure S3a Perioperative TIA Figure S3b Perioperative Haemorrhagic stroke Figure S3c Perioperative Ischaemiac stroke**

**
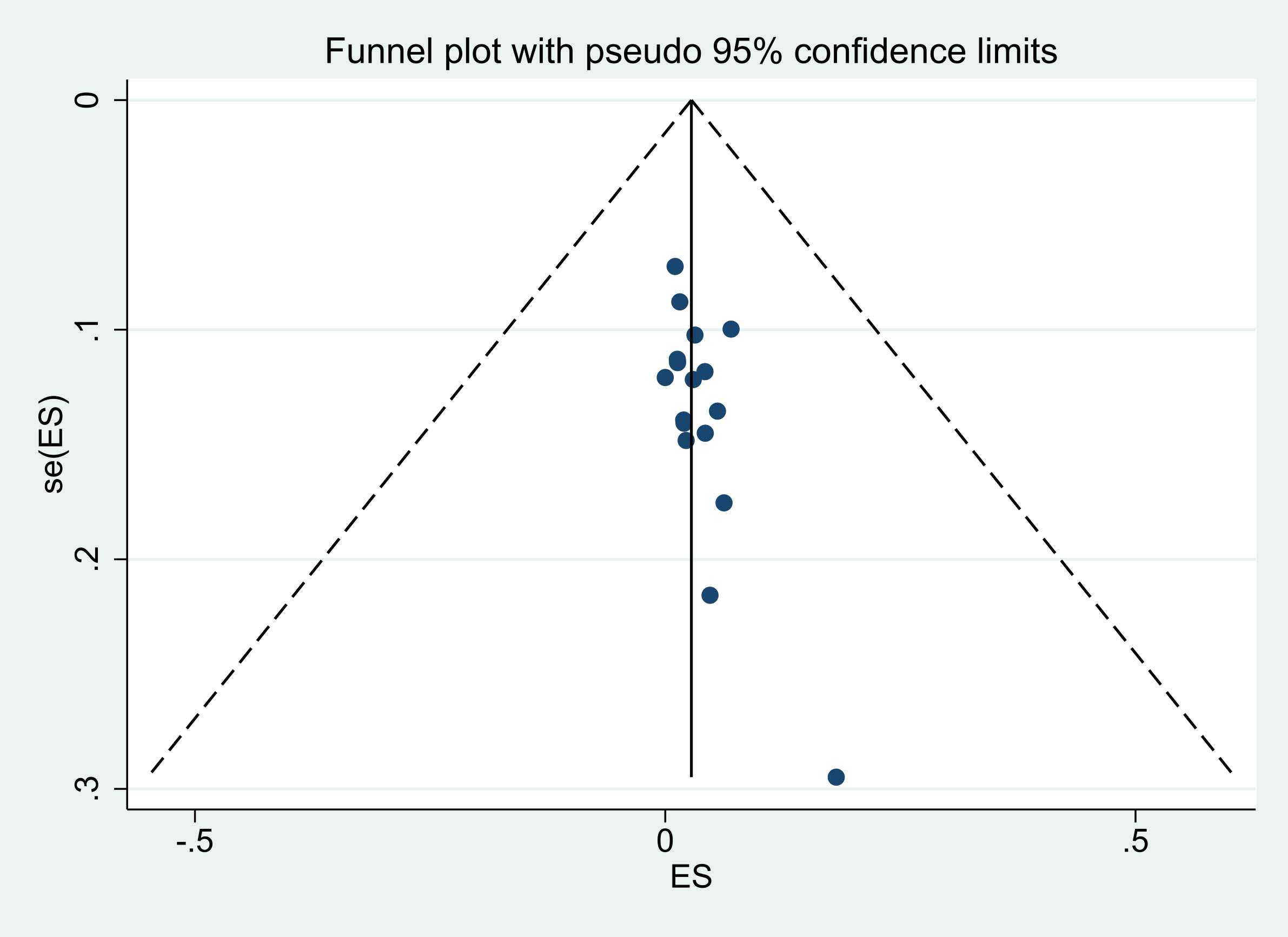

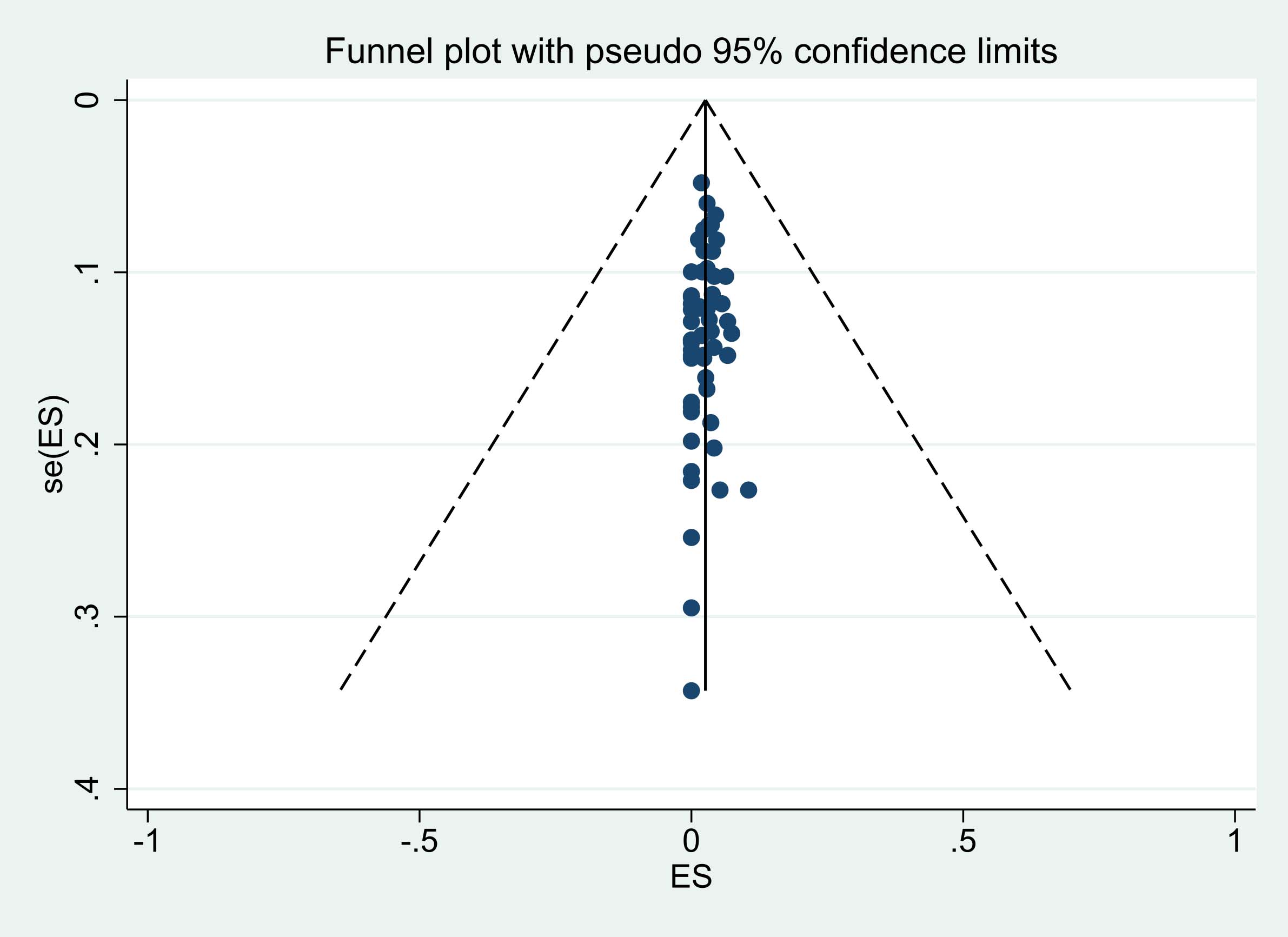

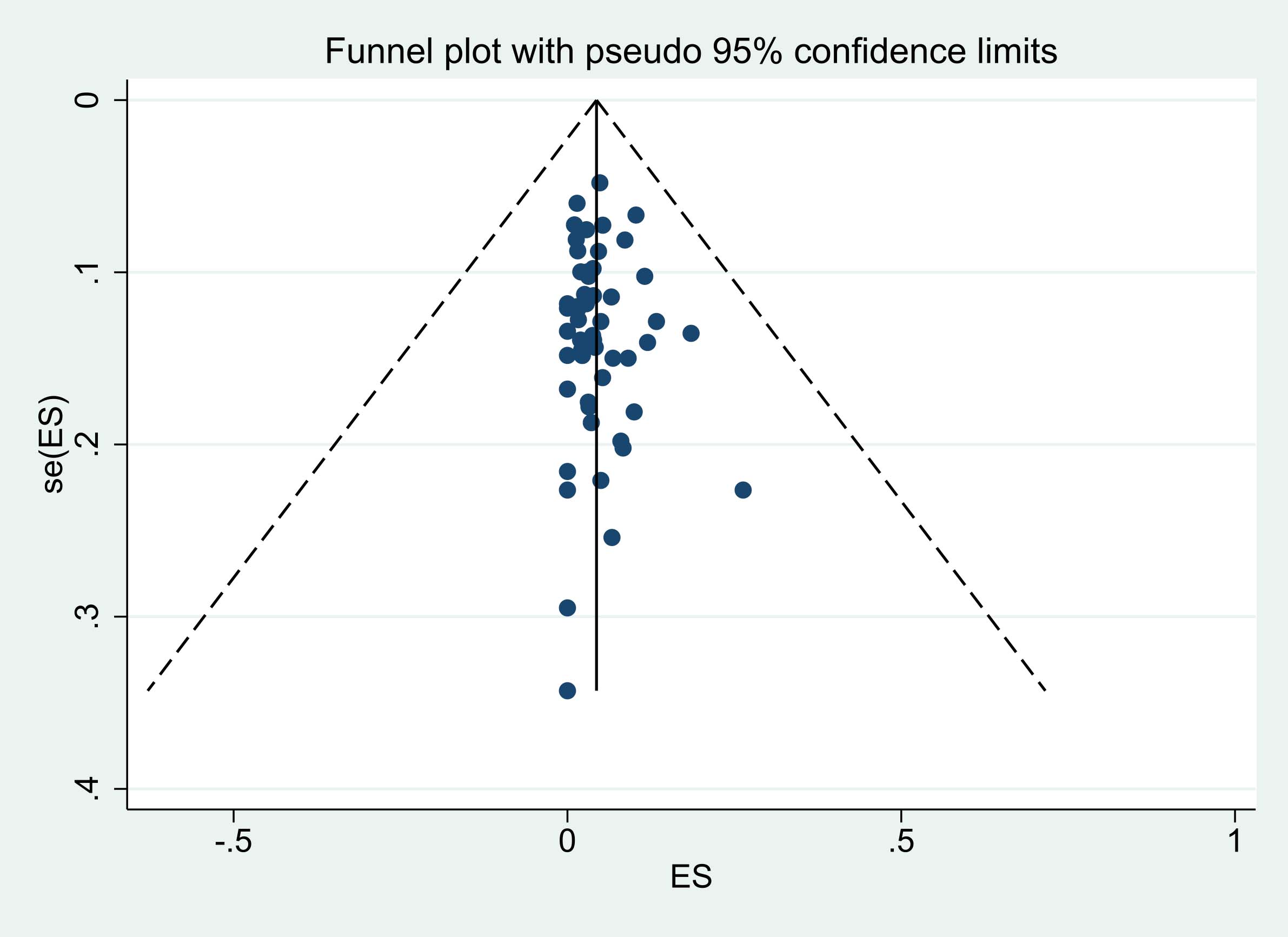
**

**Figure S3d Perioperative stroke Figure S3e Perioperative death Figure S3f Perioperative stroke or death**

**
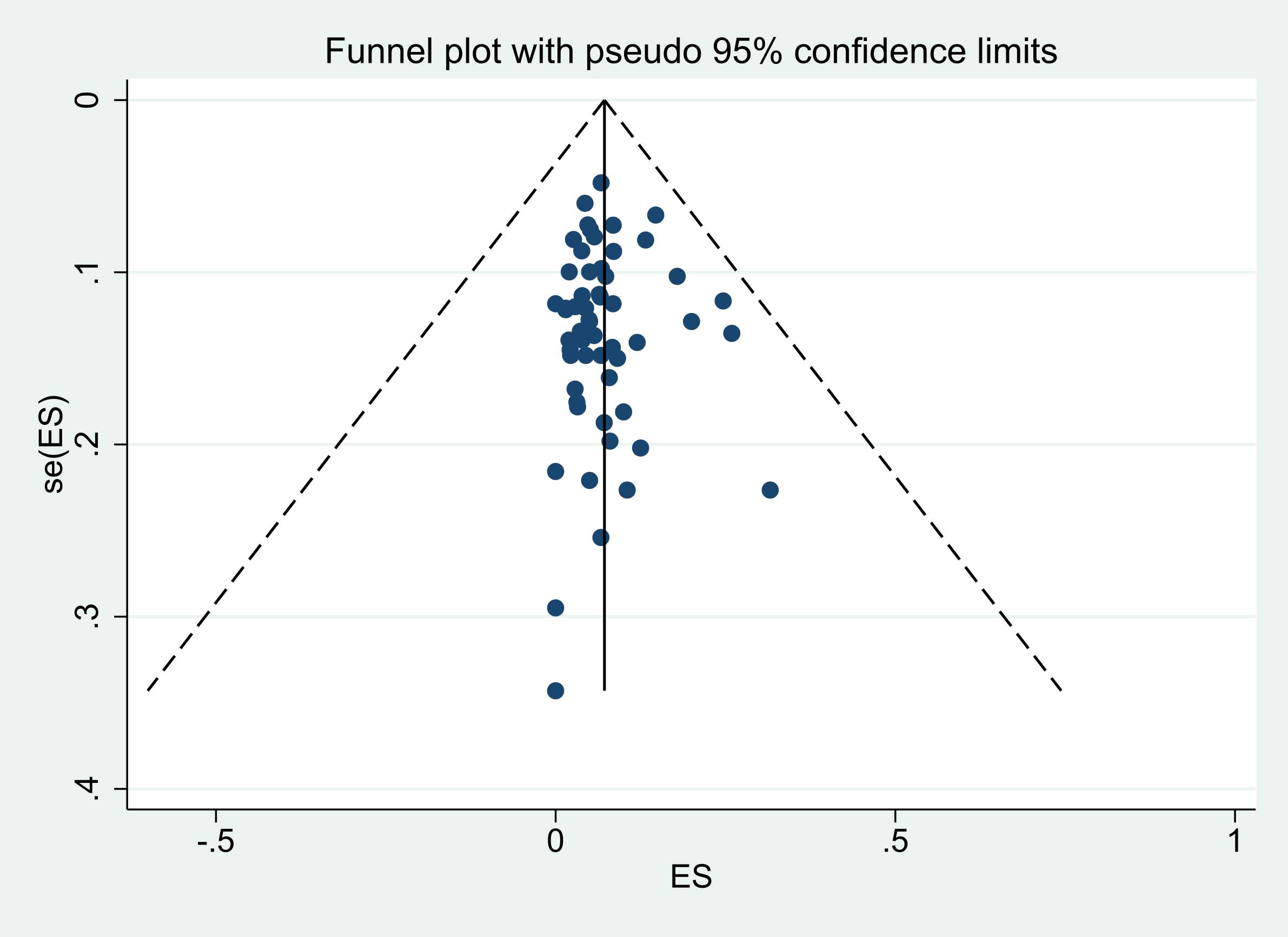

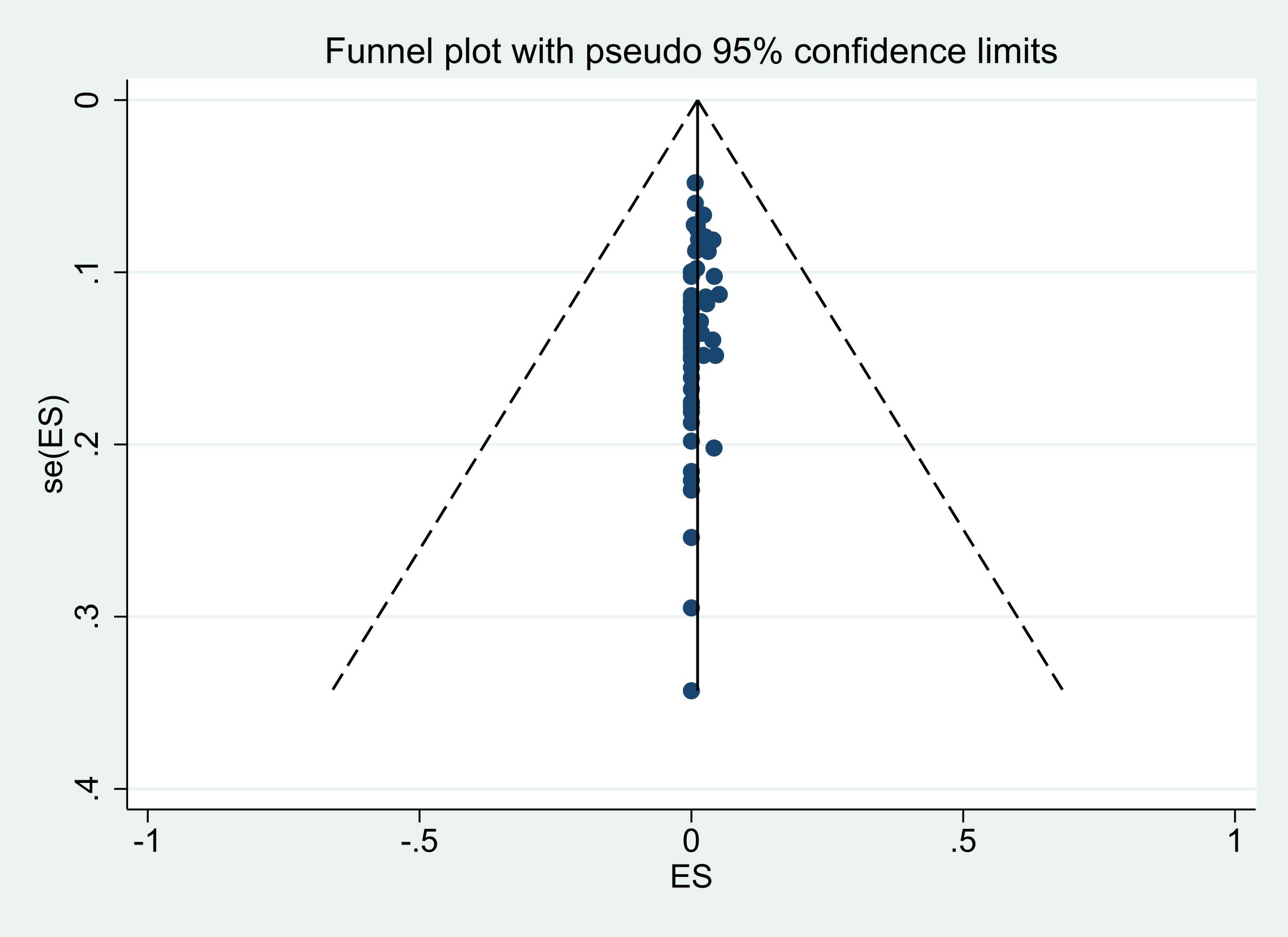

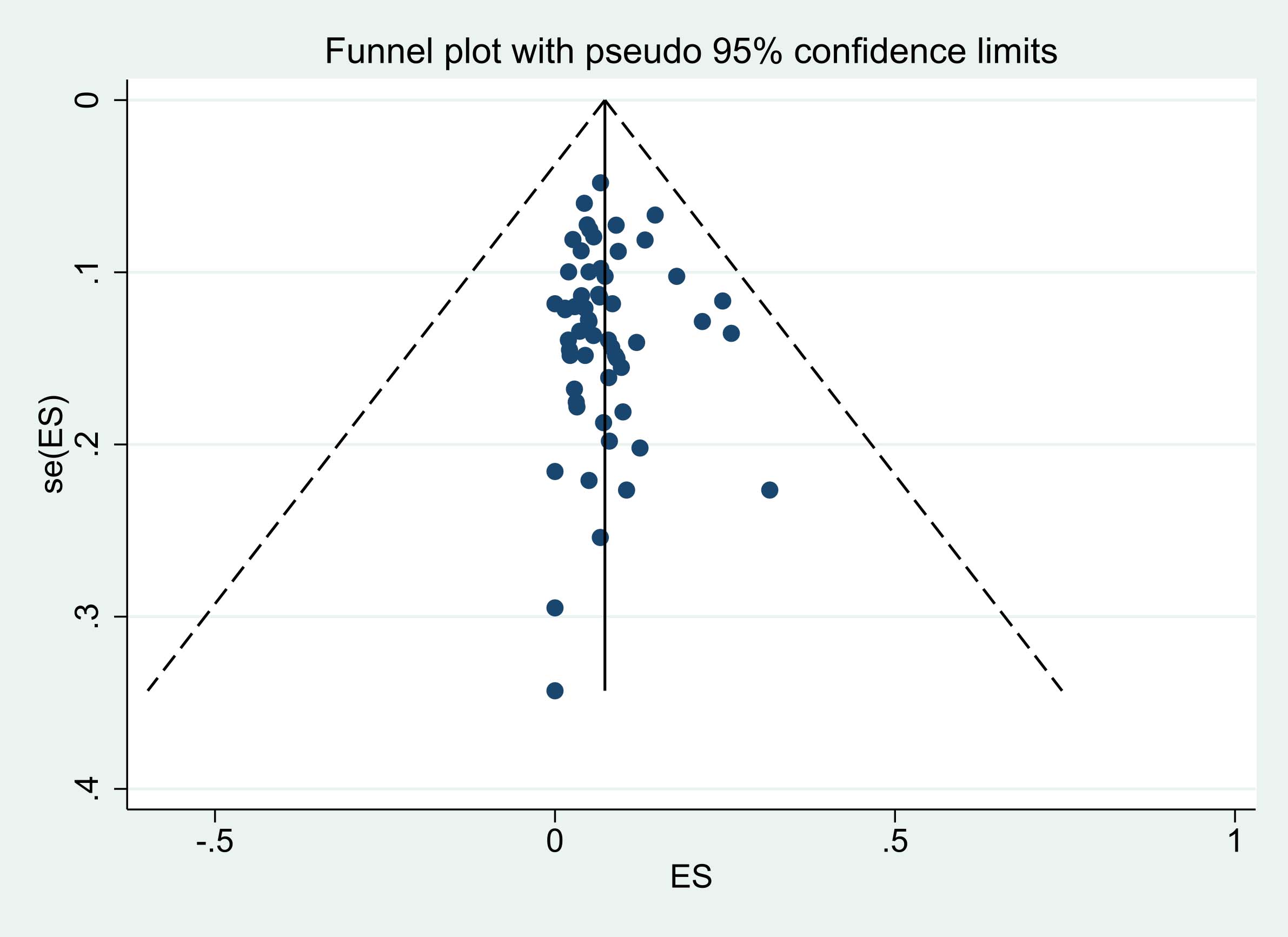
**

**Figure S3g TIA beyond 30 days Figure S3h Ischaemiac stroke beyond 30 days Figure S3i Ischaemiac stroke or TIA beyond 30 days**

**
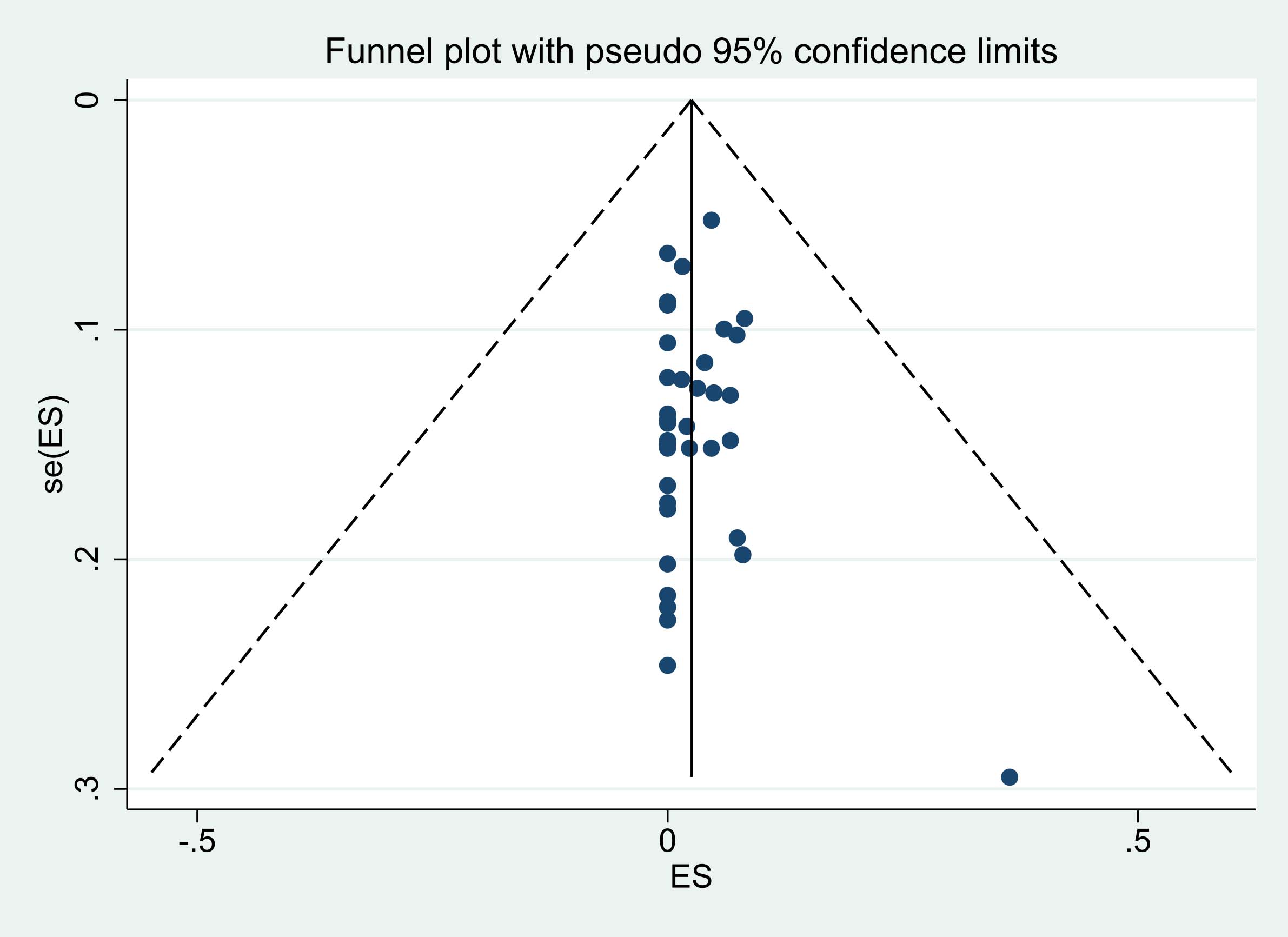

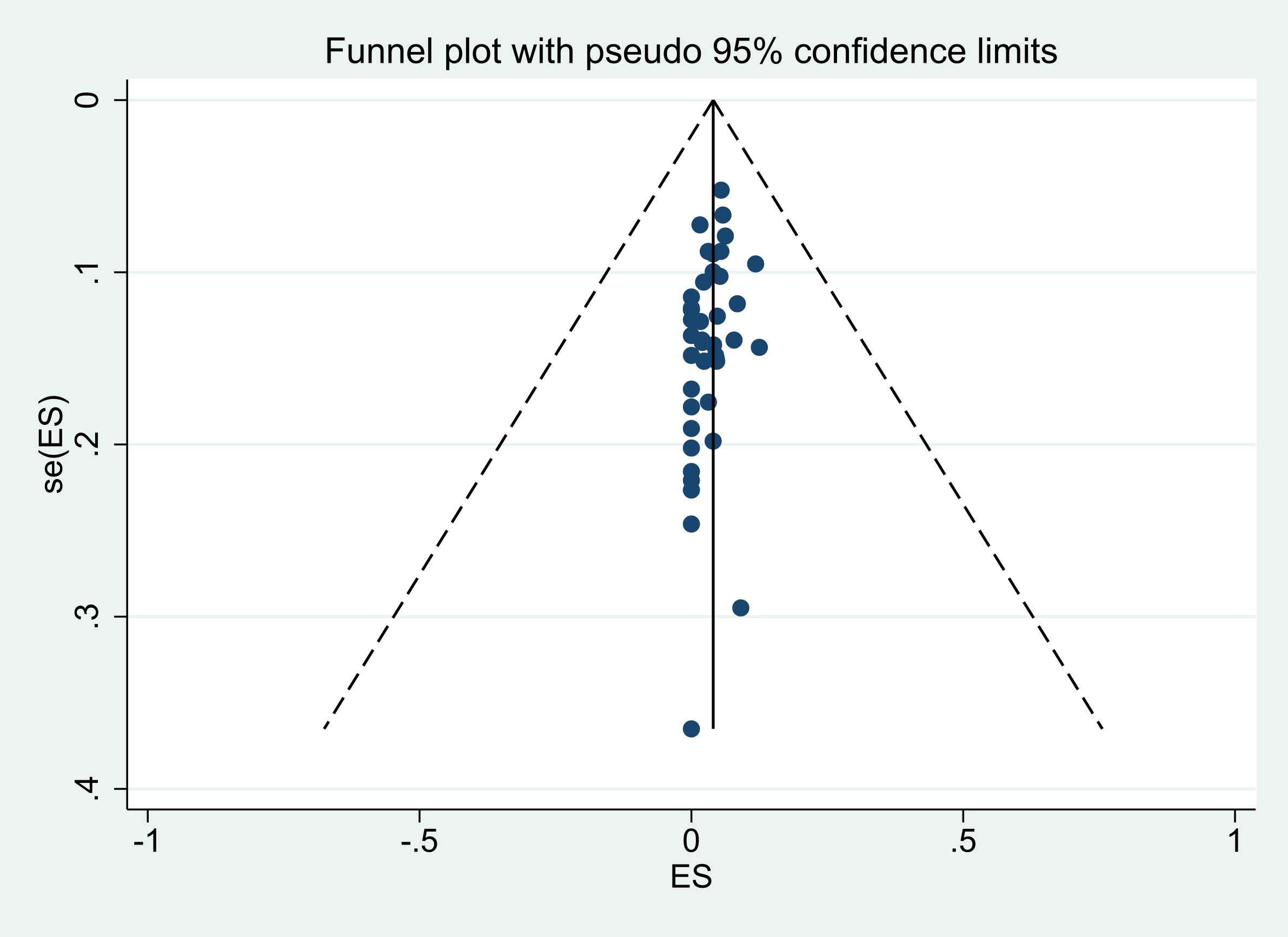

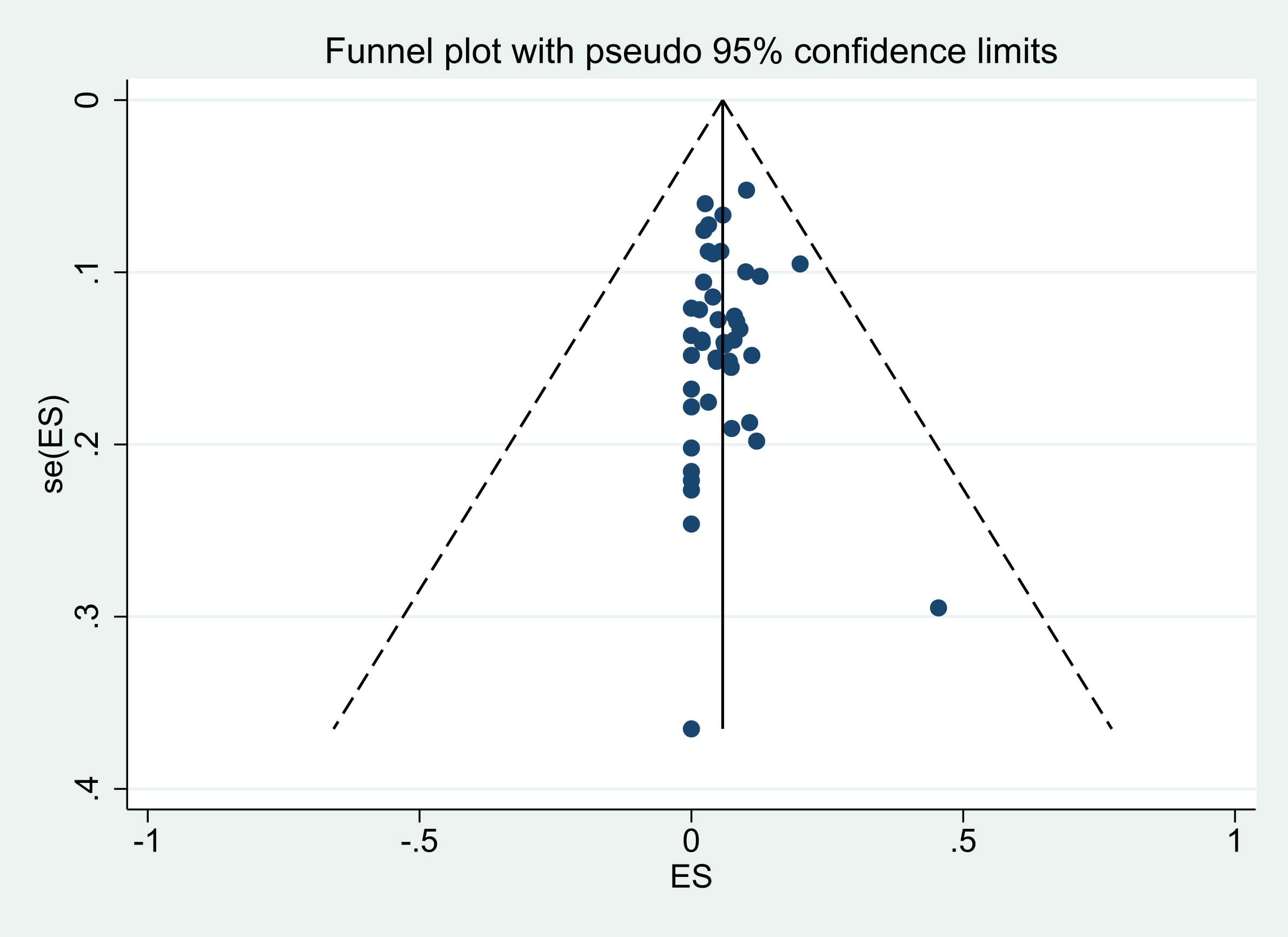
**

**Figure S3j Death beyond 30 days Figure S3k Ischaemiac stroke or death beyond 30 days Figure S3l ISR**

**
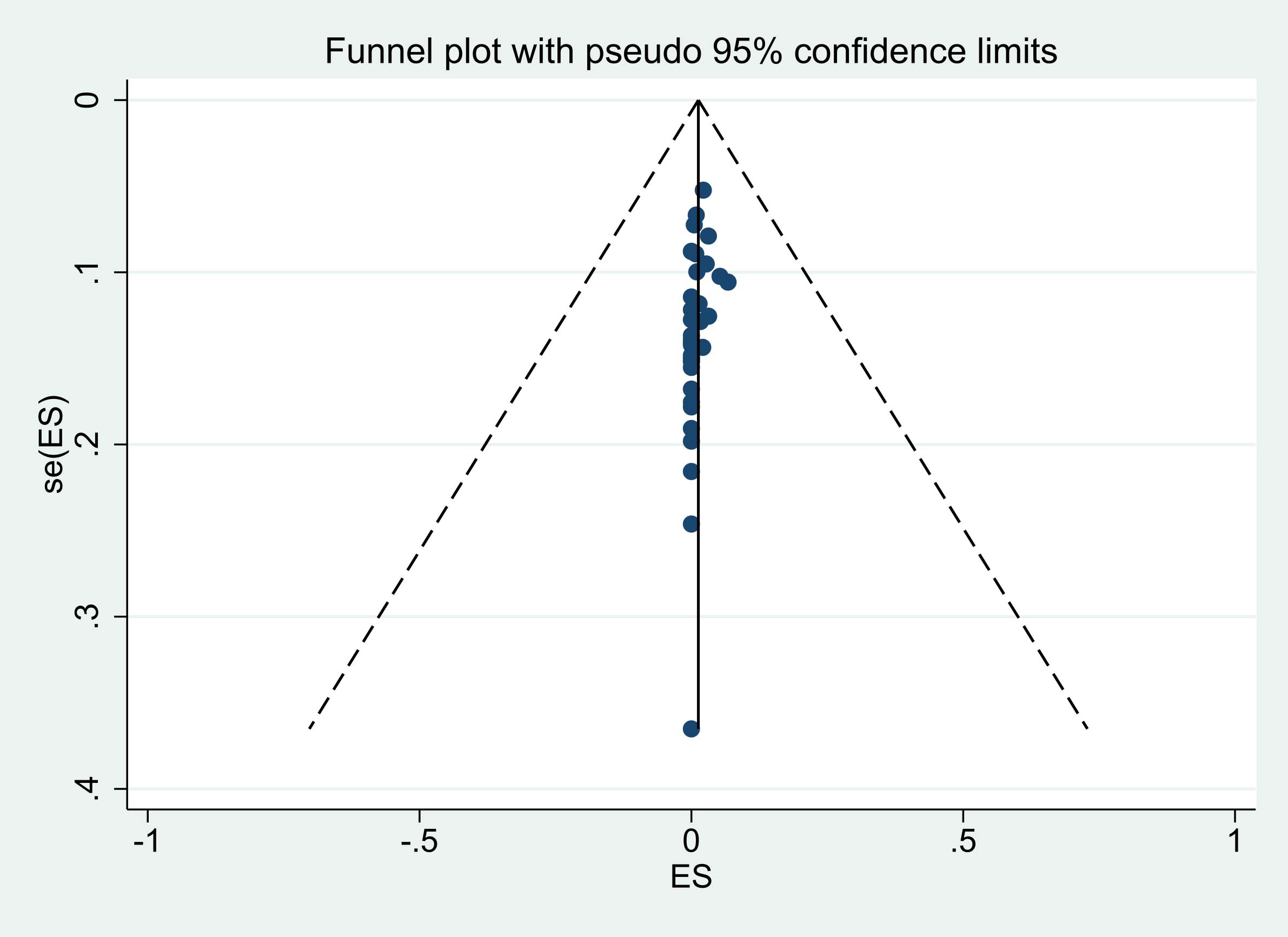

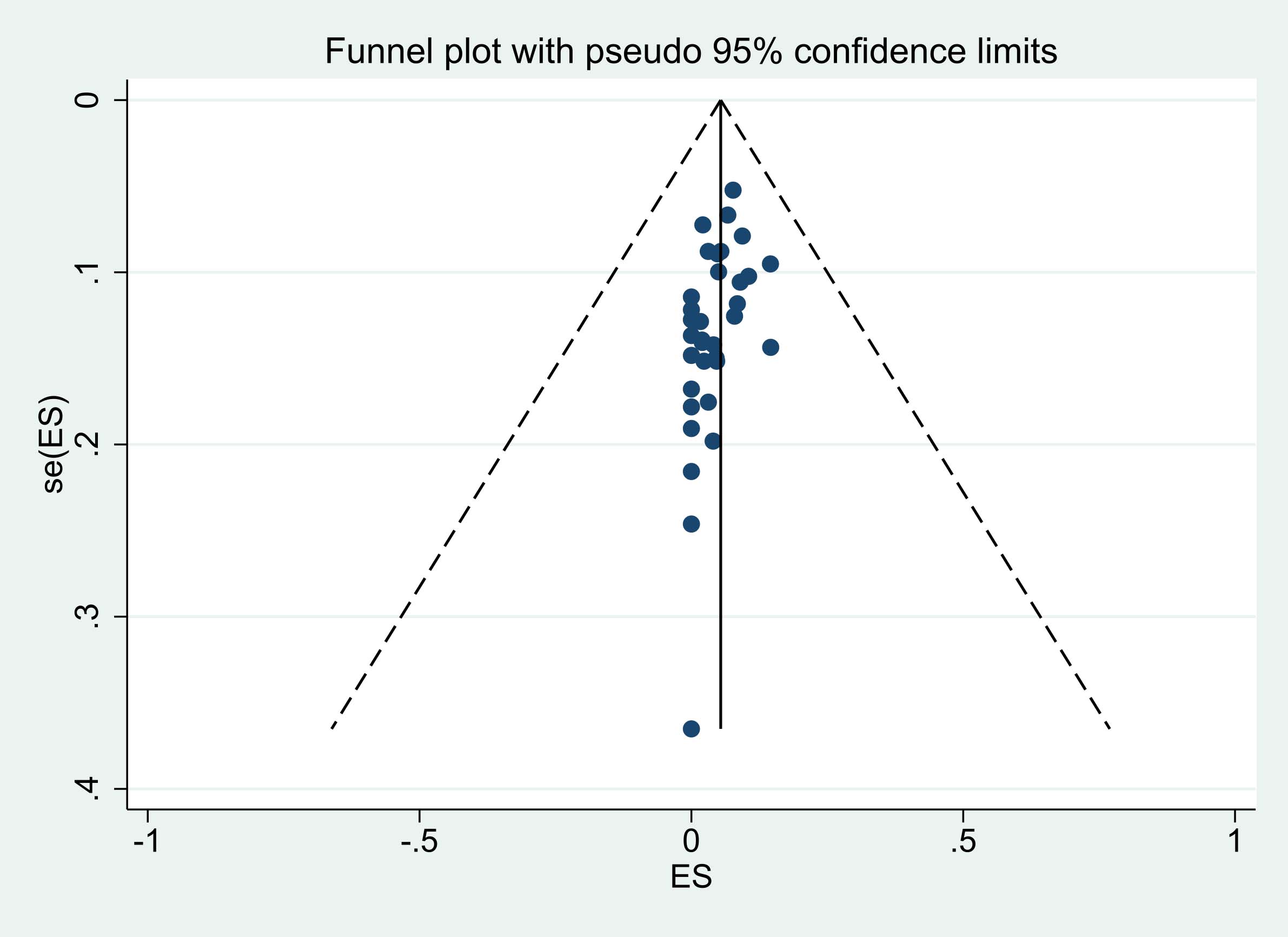

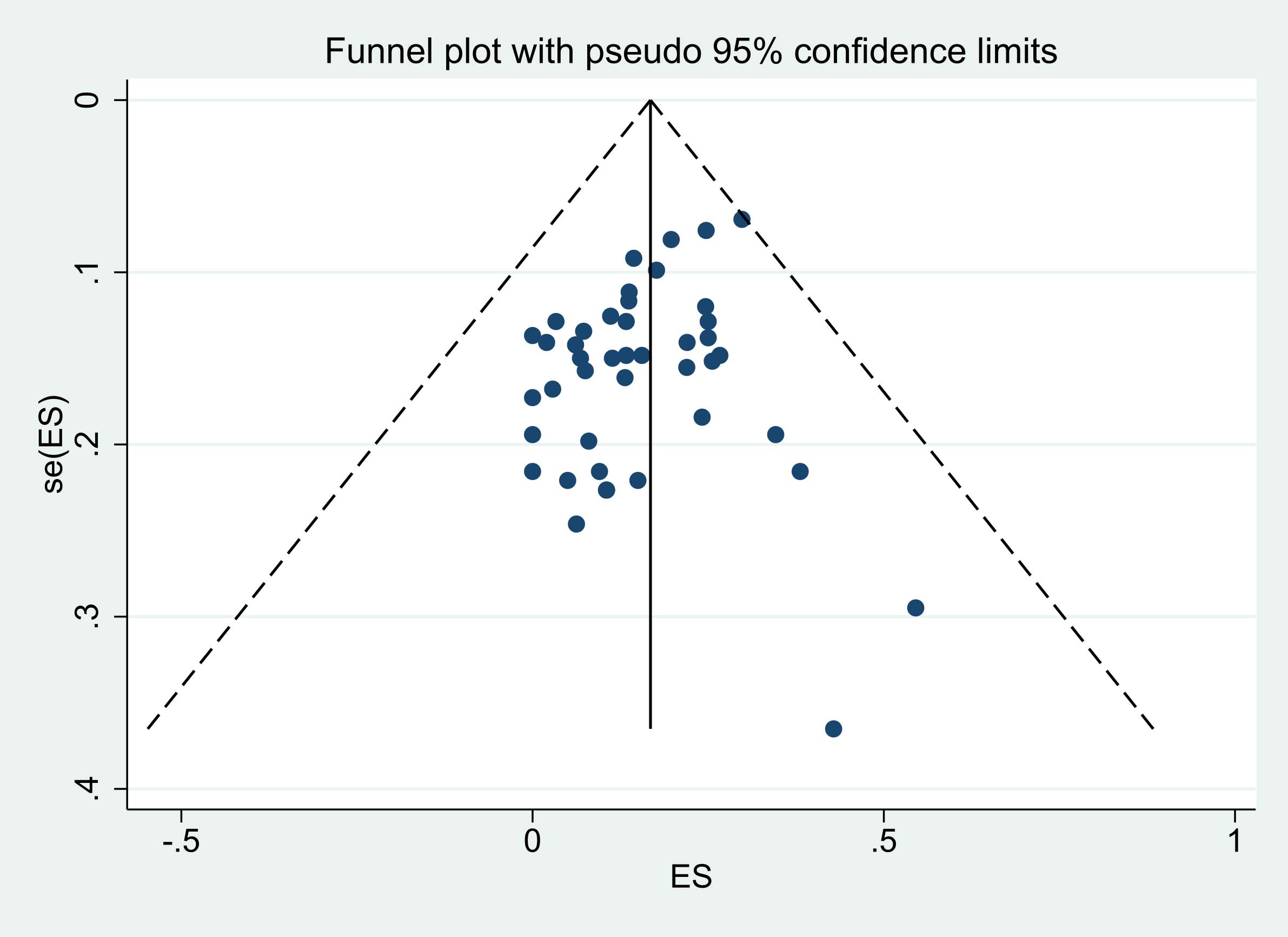
**

**Figure S3l ischemic stroke beyond 30 days through 1 year**

**
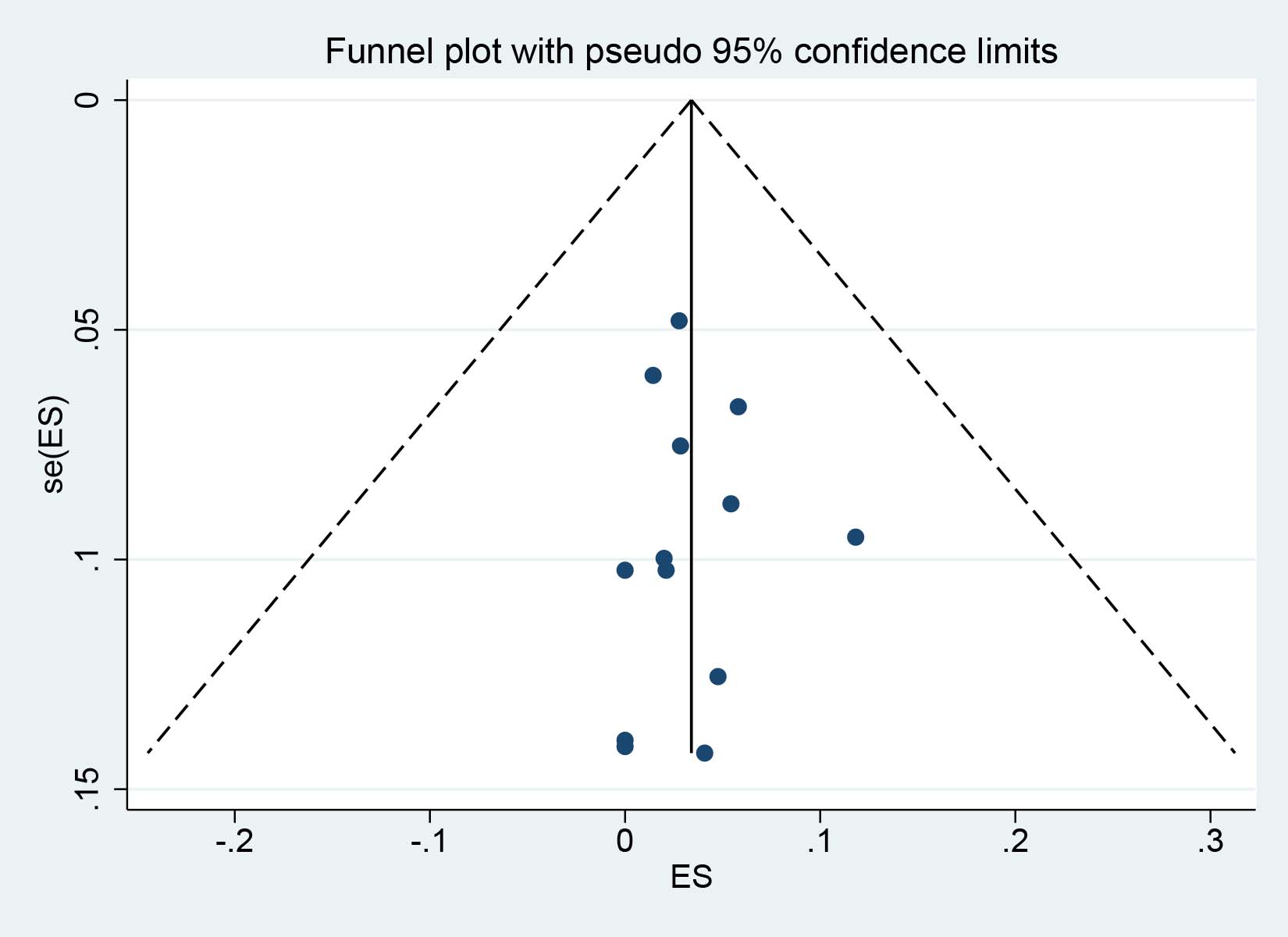
**

**Figure S4 Diagrams of sensitivity analysis**

**Figure S4a** **Perioperative Ischaemiac stroke**

**
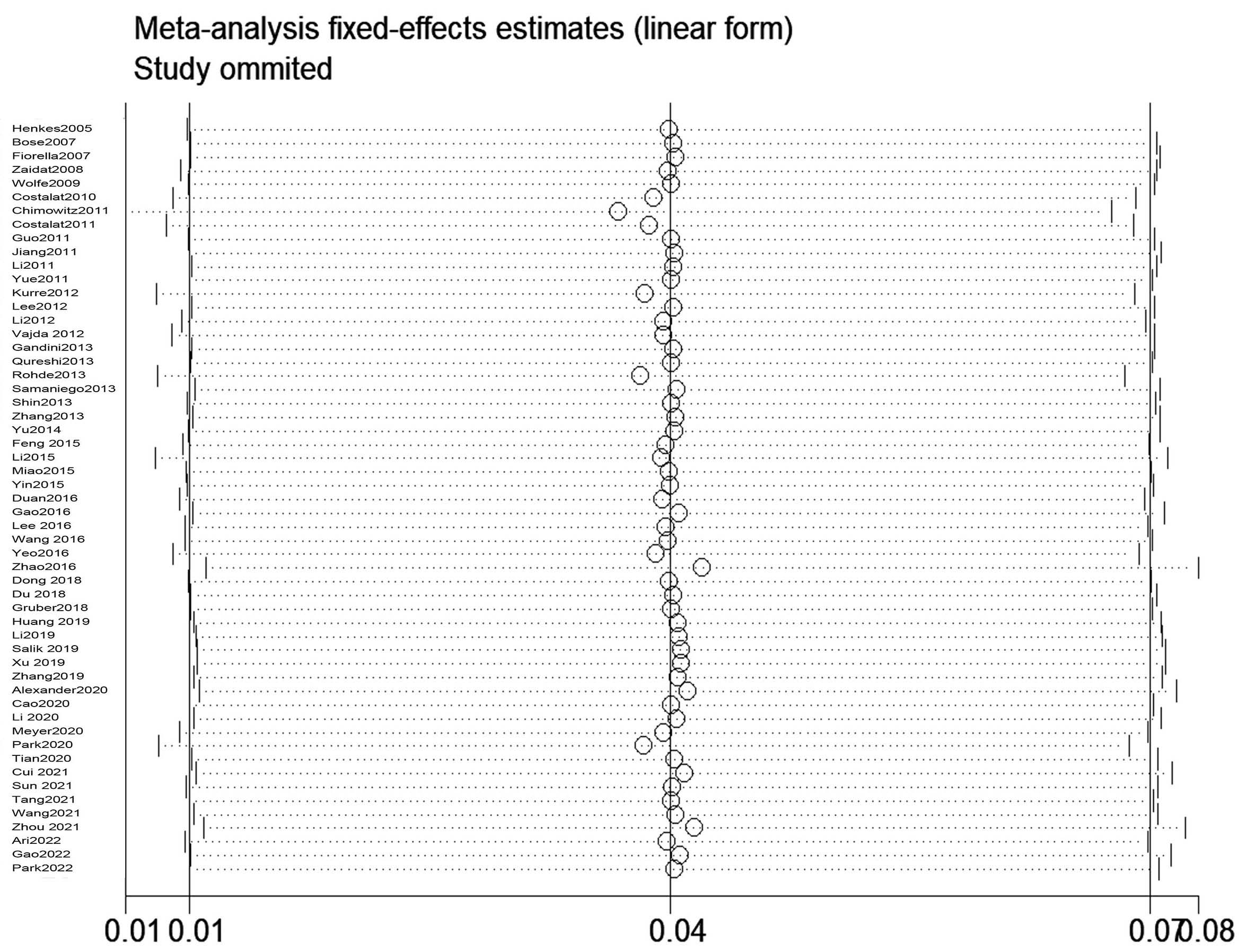
**

**Figure S4b Perioperative stroke**

**
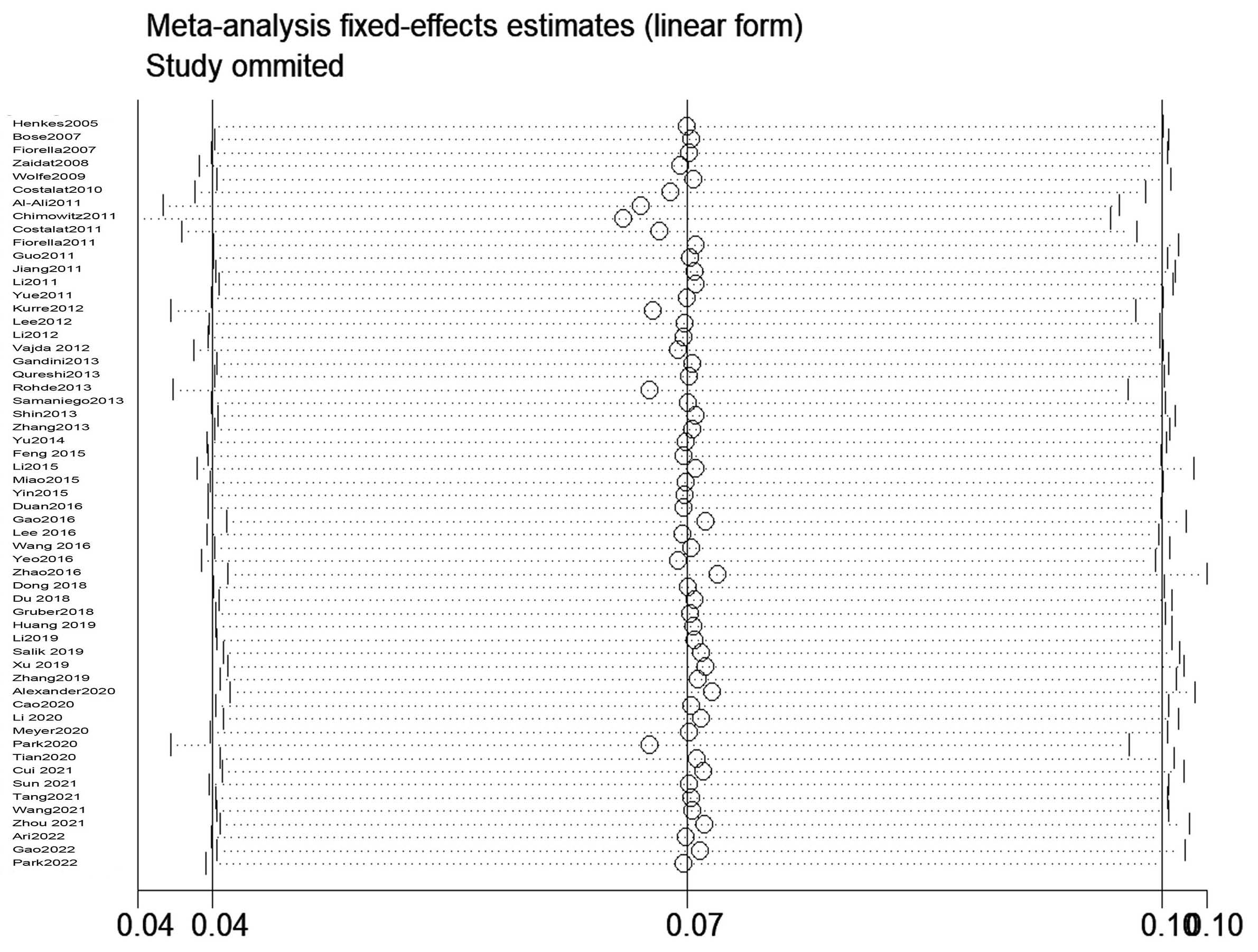
**

**Figure S4c Perioperative stroke or death**

**
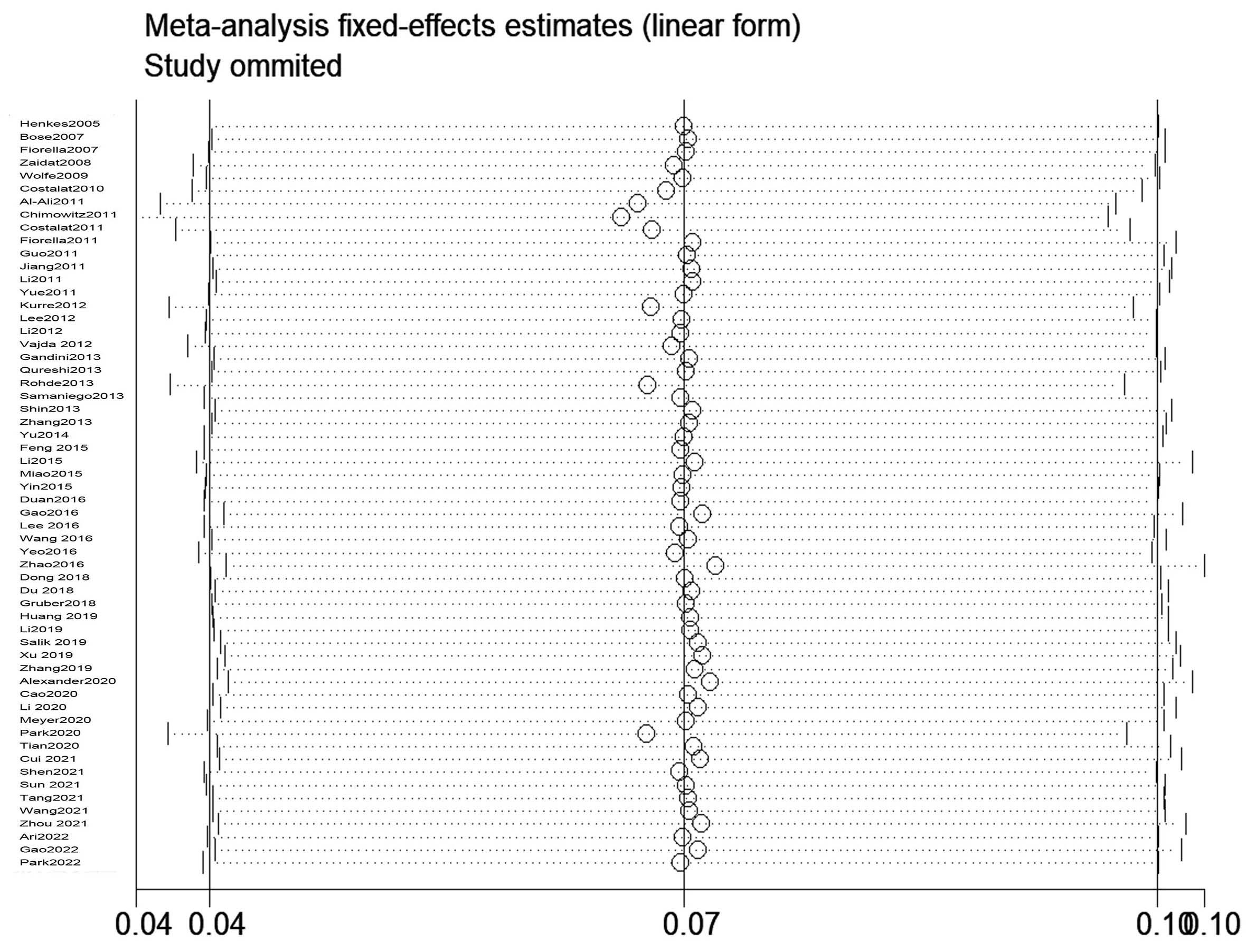
**

**Figure S4d TIA beyond 30 days**

**
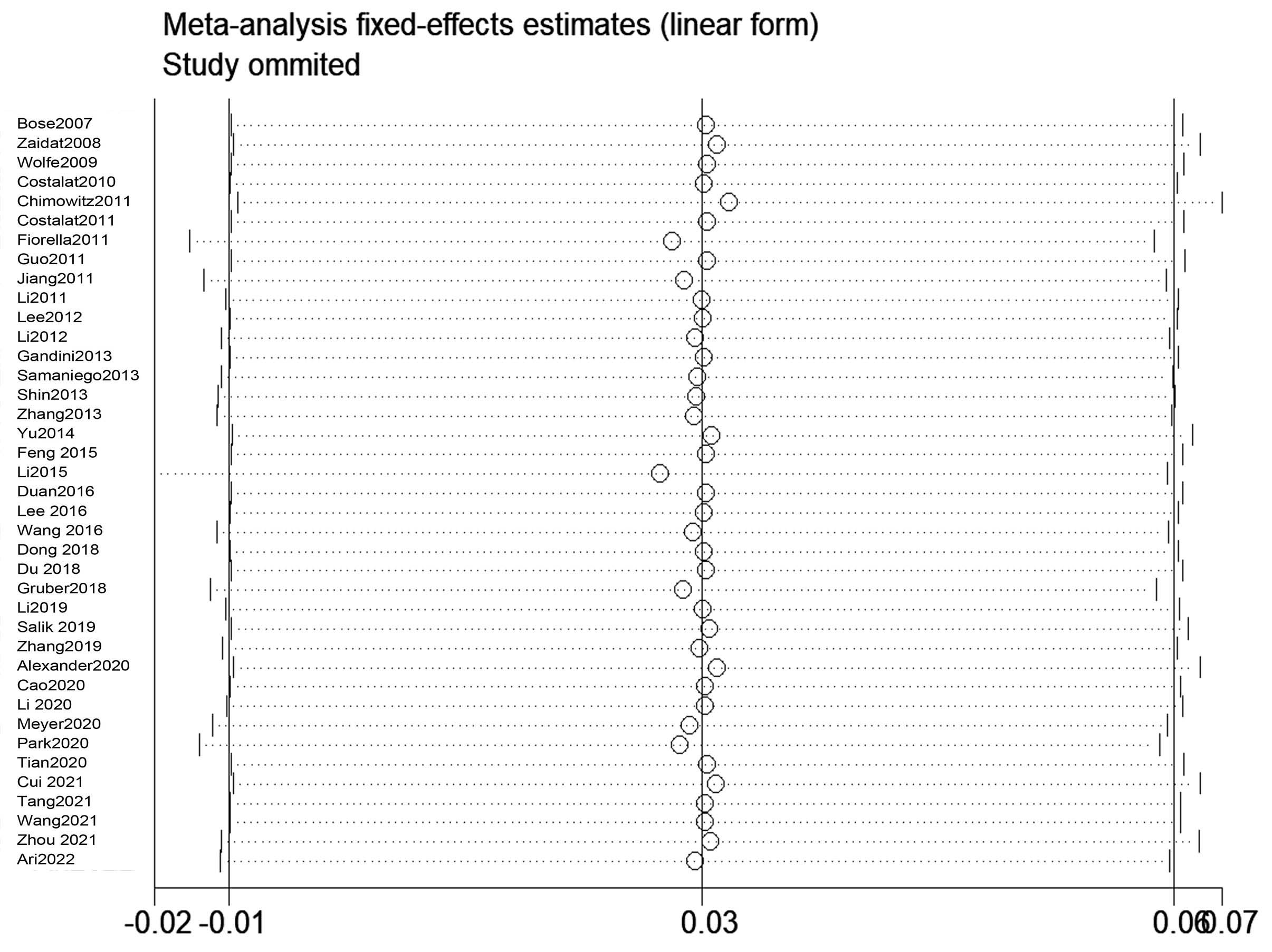
**

**Figure S4e Ischaemiac stroke or TIA beyond 30 days**

**
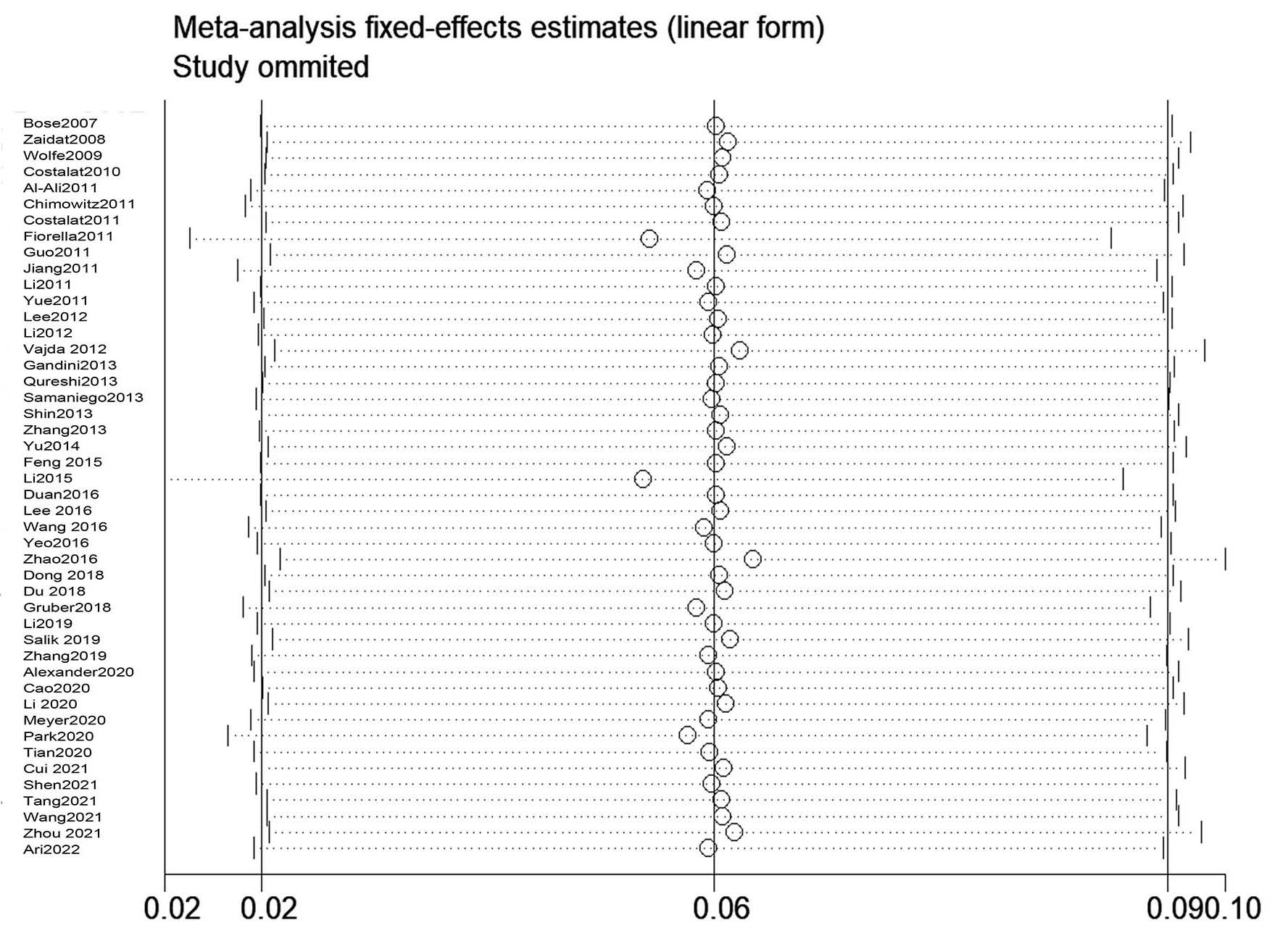
**

**Figure S4f Ischaemiac stroke or death beyond 30 days**

**
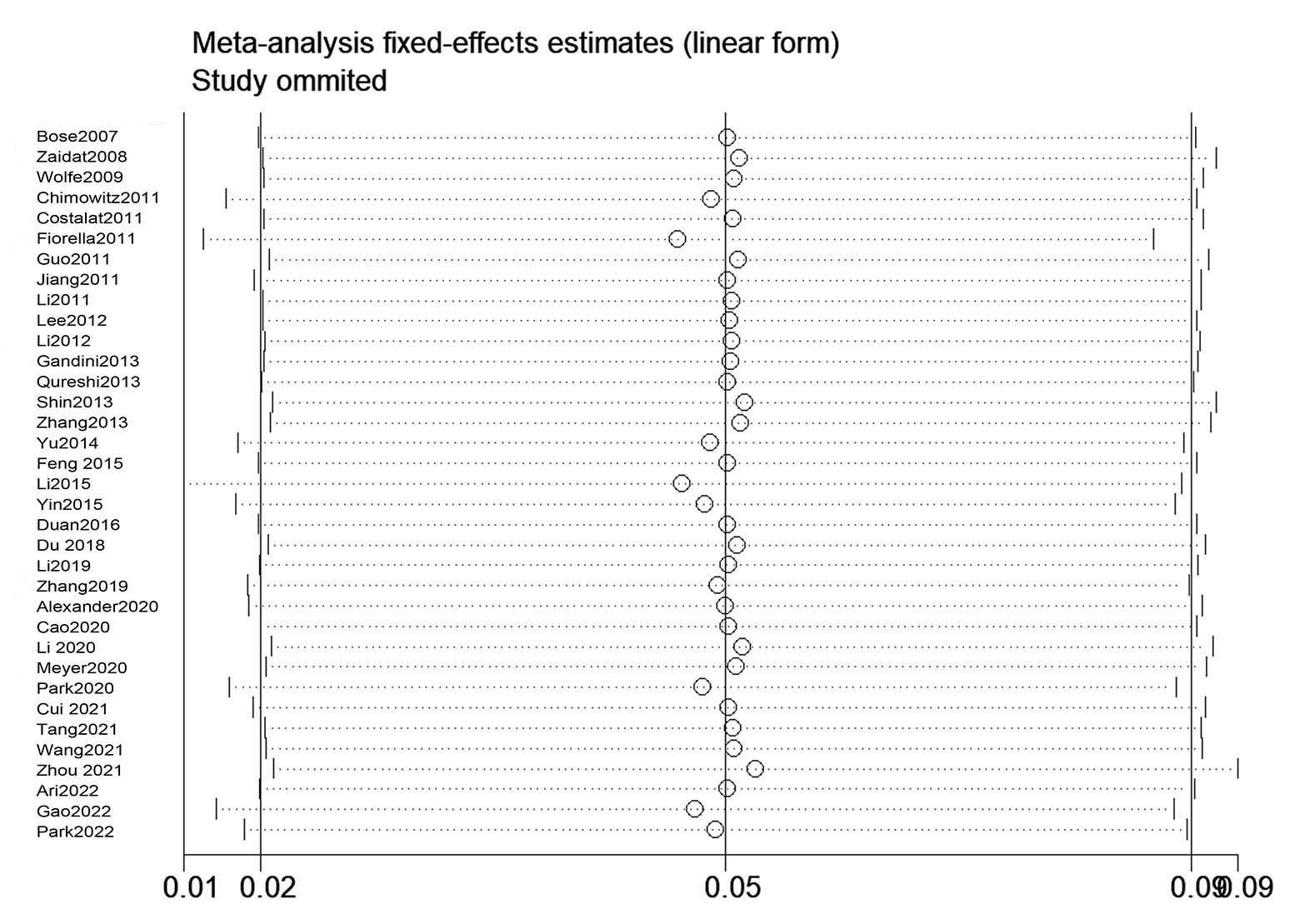
**

**Figure S4g ISR**

**
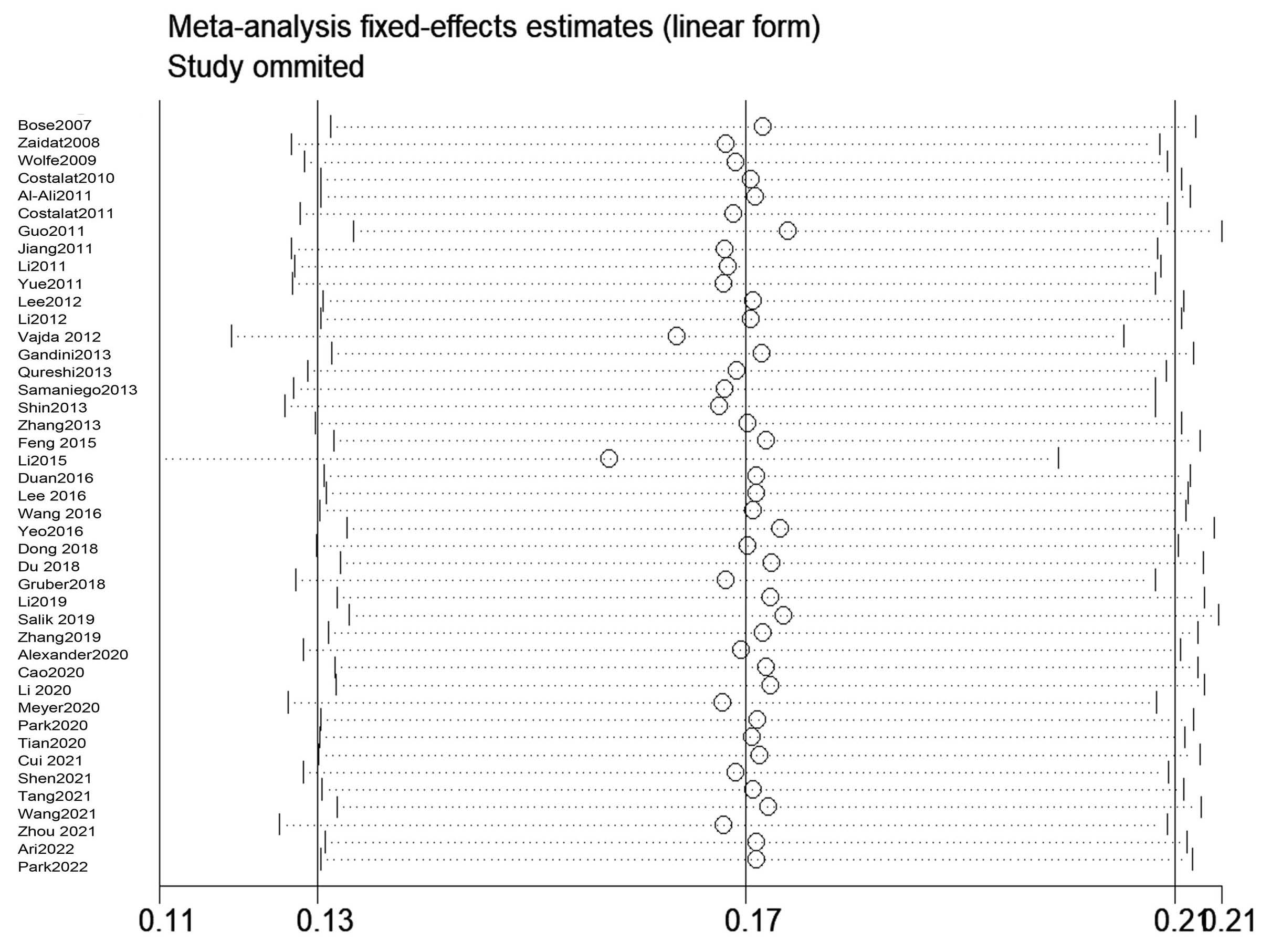
**

**Figure S4h ischemic stroke beyond 30 days through 1 year**

**
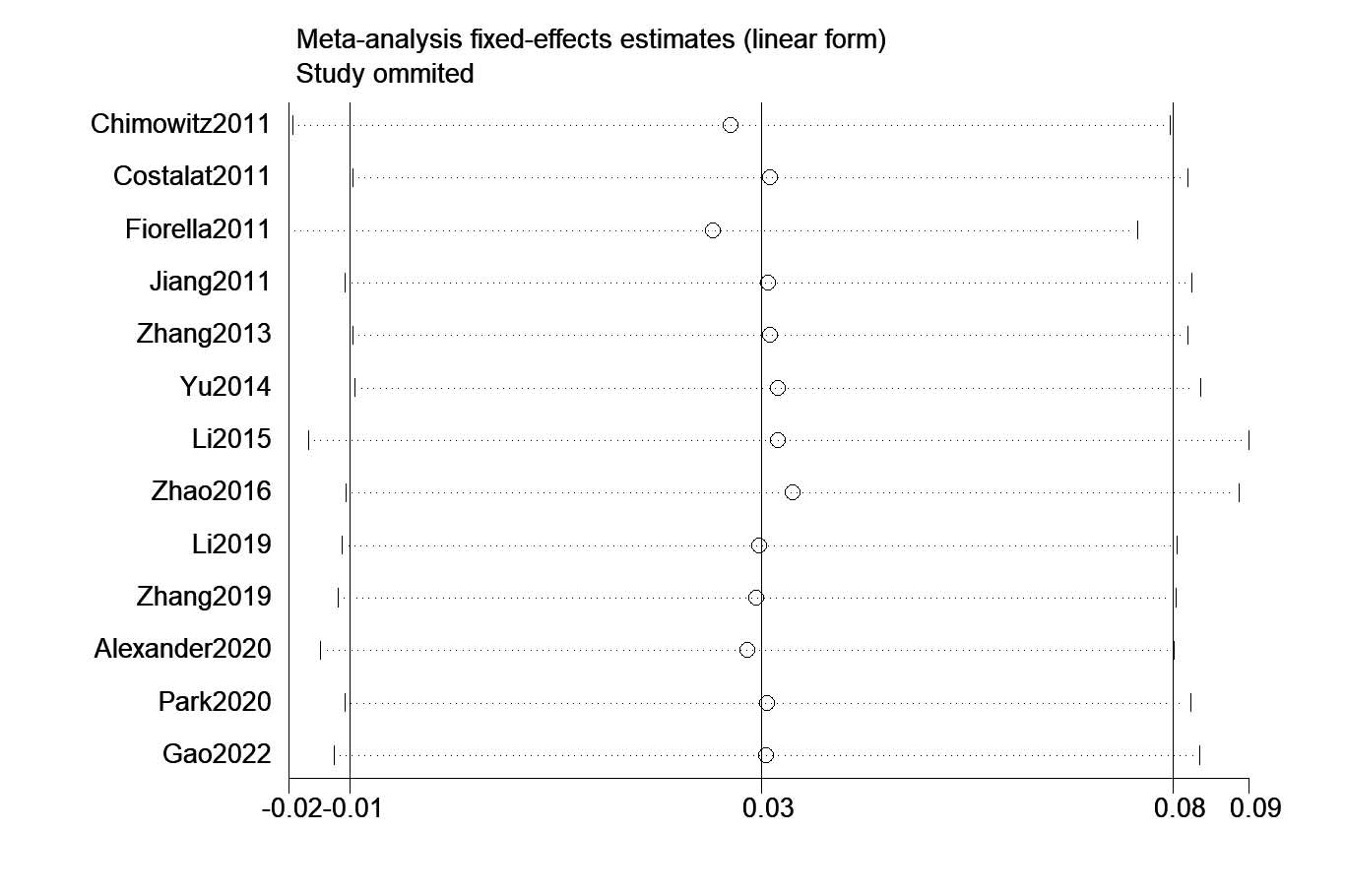
**
